# An improved method for the highly specific detection of transcription start sites

**DOI:** 10.1093/nar/gkad1116

**Published:** 2023-11-22

**Authors:** Masahide Seki, Yuta Kuze, Xiang Zhang, Ken-ichi Kurotani, Michitaka Notaguchi, Haruki Nishio, Hiroshi Kudoh, Takuya Suzaki, Satoko Yoshida, Sumio Sugano, Tomonao Matsushita, Yutaka Suzuki

**Affiliations:** Department of Computational Biology and Medical Sciences, Graduate School of Frontier Sciences, The University of Tokyo, Chiba, Japan; Department of Computational Biology and Medical Sciences, Graduate School of Frontier Sciences, The University of Tokyo, Chiba, Japan; Division of Biological Science, Graduate School of Science and Technology, Nara Institute of Science and Technology, Nara, Japan; Bioscience and Biotechnology Center, Nagoya University, Aichi, Japan; Bioscience and Biotechnology Center, Nagoya University, Aichi, Japan; Department of Botany, Graduate School of Science, Kyoto University, Kyoto, Japan; Graduate School of Bioagricultural Sciences, Nagoya University, Aichi, Nagoya, Japan; Data Science and AI Innovation Research Promotion Center, Shiga University, Shiga, Japan; Center for Ecological Research, Kyoto University, Shiga, Japan; Faculty of Life and Environmental Sciences, University of Tsukuba, Ibaraki, Japan; Tsukuba Plant-Innovation Research Center, University of Tsukuba, Ibaraki, Japan; Division of Biological Science, Graduate School of Science and Technology, Nara Institute of Science and Technology, Nara, Japan; Institute of Kashiwa-no-ha Omics Gate, Chiba, Japan; Future Medicine Education and Research Organization, Chiba University, Chiba, Japan; Department of Botany, Graduate School of Science, Kyoto University, Kyoto, Japan; Department of Computational Biology and Medical Sciences, Graduate School of Frontier Sciences, The University of Tokyo, Chiba, Japan

## Abstract

Precise detection of the transcriptional start site (TSS) is a key for characterizing transcriptional regulation of genes and for annotation of newly sequenced genomes. Here, we describe the development of an improved method, designated ‘TSS-seq2.’ This method is an iterative improvement of TSS-seq, a previously published enzymatic cap-structure conversion method to detect TSSs in base sequences. By modifying the original procedure, including by introducing split ligation at the key cap-selection step, the yield and the accuracy of the reaction has been substantially improved. For example, TSS-seq2 can be conducted using as little as 5 ng of total RNA with an overall accuracy of 96%; this yield a less-biased and more precise detection of TSS. We then applied TSS-seq2 for TSS analysis of four plant species that had not yet been analyzed by any previous TSS method.

## Graphical Abstract

**Graphical Abstract:**
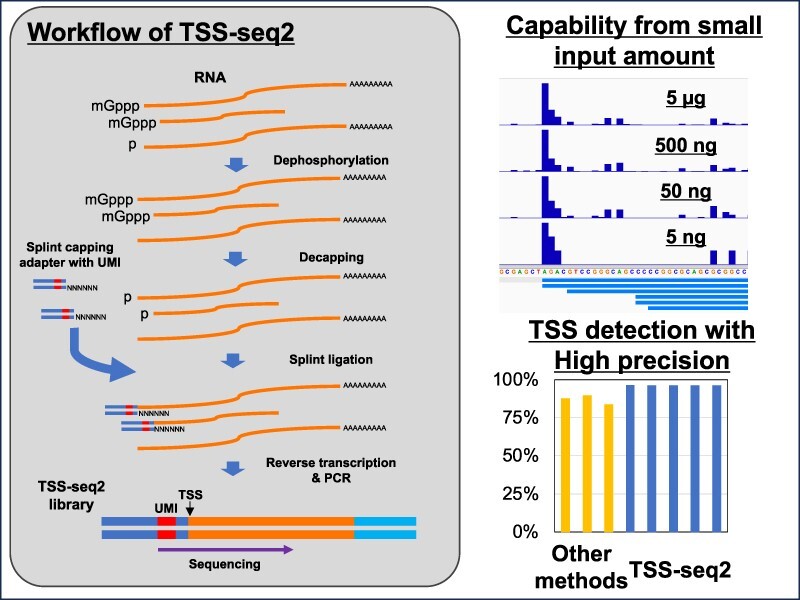

## Introduction

Identification of transcriptional start sites (TSSs) is a key step for studying the transcriptional regulation of genes. Many transcriptional *cis-*regulatory elements, such as the binding sites of transcriptional regulatory factors (TFBSs), are found in the proximal or overlapping regions of TSSs. This region is therefore collectively called the promoter (1,2). In conjunction with the TFBS, which resides at the distal region of the promoter and is called the enhancer, promoters serve as docking platforms for the basic cellular transcriptional machinery, including complexes containing RNA polymerase. It is also known that promoters and enhancers are subjected to intensive epigenomic regulation via histone modification, DNA methylation, and other mechanisms (2,3). Therefore, for the precise annotations of genes, including non-coding genes and elements of the transcriptional regulatory network, it is essential to identify TSSs and their harbored promoters within a given genome.

The genomic coordination of a TSS and its transcriptional usage are important factors for identifying the mRNAs that are transcribed from specific TSSs. For transcripts that are transcribed by RNA polymerase II, there is a specific structure called the cap at the 5′ end. Therefore, one method for the identification of TSSs can focus on selecting the cap sites of full-length mRNAs, since noncapped mRNAs are generally degraded (4).

Several methods have been developed for genome-wide cap detection, and these are generally based on unique principles (4). The most representative method is CAGE, such as nAnTi-CAGE (5). For the CAGE method, the cap structure is chemically labeled with biotin and selected for further cDNA synthesis. The CAGE method has been employed for genome-wide studies of many organisms, including humans and mice. The FANTOM consortium was organized for the annotation of TSS by collecting and storing large datasets, and its database remains pivotal for the study of transcriptional regulation.

From a technology viewpoint, recent forms of TSS analysis using small amounts of tissue or very specific cell types have been attempted by modifying the original CAGE method. These include new protocols such as SLIC-CAGE (6) and LQ-ssCAGE (7). Template switch-based methods, which use the terminal deoxynucleotidyl transferase activity of reverse transcriptase, have also been employed for related methods, including nanoCAGE (8), STRIPE-seq (9) and Smar2C2 (10). SLIC-CAGE can be performed using as little as >1 ng of total RNA by employing carrier RNA. Moreover, by using sample indexing by reverse transcription (RT), LQ-ssCAGE can use samples >25 ng of total RNA by pooling multiple samples, although it requires a high degree of multiplexing—i.e. up to several micrograms of the collective starting material for complementing a small input amount in each sample (7). Despite the modifications that have been made to the original CAGE, the core components of the CAGE procedure, which involve cap-labeling, take a long time to chemically react and require delicate handling of source material. These drawbacks have hampered its application in genome biology, especially for non-experts or for those for annotating newly sequenced nonmodel organisms. In plants, intensive CAGE analysis has been conducted only for Arabidopsis (11) and Maize (12), and therefore most atypical plants have uncharacterized TSSs.

Our group also developed a method, designated ‘TSS-seq,’ which is based on a totally different principle. This method employs a series of enzymatic reactions, named ‘oligo-capping,’ to label the cap structure (13,14). For this oligo-capping method, enzymatic reactions proceed in far milder reaction conditions. Despite its potential, the generally poor reaction efficacy has prevented its wide use for small tissue samples. For example, the original TSS-seq procedure requires 50 μg of total RNA as starting material. In addition, it does not include any means by which to discriminate against PCR duplicates. The substantial loss of material occurs mainly in two steps: low reaction efficiency during RNA ligation and further loss during subsequent purification steps. For the oligo-capping step, cap selection is achieved via the selective ligation of a synthetic oligo to the 5′ end of mRNAs where the cap originally resided. At this step, the RNA ligase should orientate both templates, i.e. the synthetic oligo and a full-length mRNA, which are freely distributed in the reaction.

In this study, we report the development of TSS-seq2, an oligo-capping-based method for the detection of the TSS sites that employs splint ligation and unique molecular identifiers (UMIs) (15). We found that TSS-seq2 can be performed from a sample of total RNA of only 5 ng. We then compared the performance of TSS-seq2 to other TSS detection methods. We found that TSS-seq2 showed equivalent or better performance with respect to sensitivity and specificity, relative to other methods. In addition, to further demonstrate the performance of TSS-seq2, we constructed several TSS catalogs from atypical model plant species. Four species of atypical model plants, i.e. *Nicotiana benthamiana*, *Lotus japonicus*, *Phtheirospermum japonicum* and *Arabidopsis halleri* subsp. *gemmifera* (*A. halleri*, hereafter), were subjected to this analysis. Here, we describe the development and the application of TSS-seq2 as an improved, more precise, and more robust method for TSS analysis.

## Materials and methods

### Cultivation of a human cell line

A human lung cancer cell line A549 (16) was cultivated in a DMEM medium (Thermo Fisher Scientific) containing 10% FBS (Corning), 1 × l-glutamine (Thermo Fisher Scientific), 1 × nonessential amino acids (Sigma-Aldrich) and 1 × Antibiotic-Antimycotic (Thermo Fisher Scientific) in a 5% CO_2_ incubator.

### RNA extraction from cultured human cell line

Total RNA was prepared from the cultured cells using an RNeasy Mini Kit (Qiagen). Total RNA was quantified using an RNA 6000 Nano Kit and a 2100 Bioanalyzer (Agilent Technologies). The RIN value of the RNA samples extracted from A549 was ∼10.

### Fragmentation of A549 total RNA

Total RNA extracted from A549 was fragment using NEBNext Magnesium RNA Fragmentation Module (New England Biolabs). 2 μl of fragmentation buffer was added to 18 μl of RNA. After incubation at 94°C for 1 or 2 min, 2 μl of fragmentation stop solution was immediately added to the reaction. The fragmented RNA was purified using RNA Clean & Concentrator-5 (Zymo Research) following the manufacturer's instructions. The purified RNA was quantified using a Qubit RNA HS Assay Kit and a Qubit 4 Fluorometer (Thermo Fisher Scientific). DV200 value of the RNAs was measured using an RNA 6000 Nano Kit and a 2100 Bioanalyzer.

### Preparation of capped ERCC RNA

ERCC RNA Spike-In Mix (Thermo Fisher Scientific) were ‘capped’ using the Vaccinia Capping System (New England Biolabs) following a protocol released from the ENCODE project (https://www.encodeproject.org/documents/e909542d-44c0-4bee-9aac-4d41a0b768db/@@download/attachment/ENCODE_Protocol_Spikeins_capping_v1.pdf). 45.75 of Nuclease-free water and 1.35 μl of RNasin Plus Ribonuclease Inhibitor (Promega) were added to 5 μl of ERCC RNA. For denaturation of RNA, this mixture was incubated at 65°C for 5min, then on ice for 5 min. 7 μl of 10 × capping buffer, 3.5 μl of 10 mM GTP, 3.5 μl of 2 mM SAM, and 3.5 μl of vaccina capping enzyme were added to the denaturated RNA, and the mixture was incubated at 37°C for 2 h. The capped RNA was purified using 126 μl of RNA Clean XP (Beckman Coulter) before being eluted in 25 μl of nuclease-free water. The purified RNA was quantified using a Qubit RNA HS Assay Kit and a Qubit 4 Fluorometer (Thermo Fisher Scientific).

### Preparation of plant RNA

RNA samples from *N. benthamiana, L. japonicus, P. japonicum* and *A. halleri* were prepared in duplicate (Supplementary Table S1). Details of the conditions for growth and preparation of RNA are described in the Supplementary Method section. Extracted RNA was quantified using an RNA 6000 Nano Kit and a 2100 Bioanalyzer.

### Preparation of splint adapters for oligo-capping

The sequences of the custom oligos used in this study are shown in Supplementary Table S2. Splint adapters were prepared as per the method described by a previous study with some modifications (17). Briefly, 100 μM splint oligo and 100 μM capping oligo were prepared by suspension in an annealing buffer (10 mM Tris–HCl (pH7.5), 50 mM NaCl and 0.1 mM EDTA (pH 8.0)), respectively. For the 5′ splint adapters, 10 μl of 100 μM 5′ splint oligo and 5 μl of 100 μM 5′ capping oligo were mixed. After denaturation at 82°C for 2 min, the oligos were annealed by decreasing the temperature by 0.1°C/s to 4°C, as per the ramp rate options of the T100 or C1000 thermal cyclers (Bio-Rad).

### Library preparation of TSS-seq2

5 ng–5 μg of total RNA or 5 ng of capped ERCC RNA was used for TSS-seq2 library preparation. For denaturation, total RNA was incubated at 65°C for 3 min, then on ice for 2 min. To removal the phosphate group from RNA including the truncated RNA without damaging the 5′ cap structure, 2.5 μl of alkaline phosphatase (*E. coli* C75) (Takara Bio), 1.35 μl of RNasin Plus Ribonuclease Inhibitor (Promega), and 10 μl of 5 × BAP buffer (500 mM Tris–HCl (pH 7.0) and 0.36% 2-mercaptoethanol) were added to 36.15 μl of total RNA, and the mixture was incubated at 37°C for 1 h. The dephosphorylated RNA was then purified using 90 μl of RNA Clean XP before being eluted in 42.65 μl of nuclease-free water. Next, 1 μl of mRNA Decapping Enzyme, 5 μl of 10 × mRNA Decapping Enzyme Reaction Buffer (New England Biolabs), and 1.35 μl of RNasin Plus Ribonuclease Inhibitor were then added to the purified RNA, and the resulting mixture was then incubated at 37°C for 1 h. The decapped RNA was purified using 90 μl of RNA Clean XP before being eluted in 38.15 μl of nuclease-free water. Subsequently, 2.5 μl of T4 RNA Ligase 2 (New England Biolabs), 5 μl of 50% PEG8000 (New England Biolabs), 5 μl of 10 × T4 RNA Ligase 2 Reaction Buffer (New England Biolabs), 1.35 μl of RNasin Plus Ribonuclease Inhibitor, and 3 μl of splint adapter with UMI were added to the purified RNA. This mixture was incubated at 37°C for 1 h, after which the ligated RNA was purified using 90 μl of RNA Clean XP and was eluted in 10 μl of nuclease-free water. For primer annealing, 7.5 μl of 3.3 M sorbitol / 0.66 M trehalose (Fujifilm Wako Chemicals), 2.5 μl of 10 mM dNTPs (New England Biolabs), and 5 μl of 100 μM TSS-seq2 RT primer (see: Supplementary Table S2), and 1.35 μl of RNasin Plus Ribonuclease Inhibitor were added to purified RNA samples. This mixture was incubated at 65°C for 5 min, then at 4°C for 2 min in a thermal cycler. For reverse transcription, 2 μl of Maxima H Minus Reverse Transcriptase (Thermo Fisher Scientific), 10 μl of 5 M Betaine (Sigma-Aldrich), 10 μl of 5 × RT Buffer for Maxima H Minus Reverse Transcriptase, and 2.5 μl of 0.1 M DTT (Thermo Fisher Scientific or Fujifilm Wako Chemicals) were added to primer-annealed samples. They were then incubated in a thermocycler for 10 min at 25°C, for 30 min at 50°C, and held thereafter at 4°C. 40 μl of RNA Clean XP was then added before elution in 22 μl of nuclease-free water. In this step, it was important to remove the supernatant completely to reduce the amount of primer that was carried over. For PCR amplification, 25 μl of KAPA HiFi HotStart ReadyMix (Roche) and 3 μl of unique dual index primer mix (5 μM each; Supplementary Table S2) were added to the purified cDNA. The reaction was then amplified in a thermal cycler under the following PCR conditions: denaturation at 95°C for 3 min; 12 (for 5 μg of total RNA input), 13 (for 2.5 μg), 15 (for 1 μg), 16 (for 500 ng) or 19 cycles (for 50 ng) of 98°C for 20 s, 63°C for 15 s and 72°C for 45 s; final extension at 72°C for 2 min; and hold thereafter at 4°C. As for 5 ng of the capped ERCC RNA, the TSS-seq2 libraries in duplicate were constructed with 15 cycles of PCR. The amplified library was then purified using 40 μl of SPRI select (Beckman Coulter) and was eluted in 11 μl of nuclease-free water. In addition, in this step it was important to remove the supernatant completely to reducing the amount of primer dimers. The purified library was then quantified using a DNA 7500 Kit or a High Sensitivity DNA Kit (Agilent Technologies) and a 2100 Bioanalyzer. When the primer dimer was retained, repurification was performed by adding 8 μl of SPRI select, and the library was eluted in 10 μl of nuclease-free water. Finally, 100 single-read or 150 bp pair-end sequencing of the prepared libraries was then performed on a NovaSeq 6000 (Illumina).

### Library preparation for TSS-seq1

50 ng–5 μg of total RNA was used for TSS-seq1 library preparation. Except for the ligation and rRNA removal steps, TSS-seq1 library preparation was performed in the same way as for TSS-seq2. Single strand ligation of capping followed the same procedure as reported for TSS-seq (13,18). Briefly, the decapped RNA was eluted in 12.9 μl of nuclease-free water. For ligation of the capping oligo, 1.35 μl of RNasin Plus Ribonuclease Inhibitor, 5 μl of 10 × Ligation Buffer (0.5 M Tris–HCl (pH7.0), 0.1 M 2-mercaptoethanol), 25 μl of 50% PEG8000, 1 μl of 100 μM capping oligo, 0.5 μl of 1 M MgCl_2_, 0.25 μl of 100 mM ATP and 4 μl of T4 RNA Ligase (Takara Bio) were added to the purified RNA. This mixture was then incubated at 37°C for 1 h. Ligated RNA was then purified using 90 μl of RNA Clean XP and was eluted in 9 μL of nuclease-free water. For rRNA depletion, 1 μl of QIAseq FastSelect–rRNA HMR Kit (Qiagen) was added to the purified RNA, and the mixture was incubated in a thermal cycle at 75°C for 2 min, 70°C for 2 min, 65°C for 2 min, 60°C for 2 min, 55°C for 2 min, 37°C for 2 min and 25°C for 2 min, before being held at 4°C thereafter. rRNA-depleted samples were used directly in the RT step.

### Library preparation of 5′ RACE

The 5′ RACE libraries of *THSD7A* and *UNC13D* genes were prepared by amplifying the 5′ end of each transcript from the TSS-seq1 library (5 μg input) using gene-specific primers and the primer hybridizing capping oligo sequence (Supplementary Table S2). For the first PCR amplification, 25 μl of KAPA HiFi HotStart ReadyMix and 3 μl of a mix of a gene-specific primer and i5 primer (5 μM each; Supplementary Table S2) were added to 1 ng of the TSS-seq1 library (5 μg input), and the volume of the reaction was brought to 50 μl by nuclease-free water. The reaction was then amplified in a thermal cycler under the following PCR conditions: denaturation at 95°C for 3 min; 20 cycles of 98°C for 20 s, 63°C for 15 s, and 72°C for 45 s; then, perform the final extension at 72°C for 2 min and hold thereafter at 4°C. The amplified DNA was then purified using 40 μl of SPRI select and was eluted in 22μl of nuclease-free water. For the second PCR amplification, 25 μl of KAPA HiFi HotStart ReadyMix and 3 μl of a unique dual index primer mix (5 μM each; Supplementary Table S2) were added to the purified sample. The reaction was then amplified in a thermal cycler under the following PCR conditions: denaturation at 95°C for 3 min; 6 cycles of 98°C for 20 s, 63°C for 15 s, and 72°C for 45 s; then, perform the final extension at 72°C for 2 min and hold thereafter at 4°C. The amplified library was then purified using 40 μl of SPRI select and was eluted in 21 μl of nuclease-free water. The purified library was then quantified using a DNA 7500 Kit and a 2100 Bioanalyzer. Finally, 150 bp pair-end sequencing of the prepared libraries was then performed on an iSeq100 (Illumina).

### Data processing for TSS-seq1 and TSS-seq2

For the removal and counting of reads derived from rRNA, obtained reads were first aligned to rRNA sequences using Bowtie2 version 2.3.5.1 (19) to remove reads derived from rRNA, except for the datasets of ERCC RNA that does not contain rRNA. Using the regex method of UMItools version 0.5.5 (20), the 5′ end sequences of each read containing 10 bp of UMI and 4 bp of linker sequence were trimmed, and the information of the extracted UMI sequence was appended to each read. Trimmed reads were aligned to human reference genome hg38, to the sequences of ERCC transcripts obtained from the manufacturer's website (https://assets.thermofisher.com/TFS-Assets/LSG/manuals/cms_095047.txt), or to the respective plant genome (i.e. *N. benthamiana* Nbe.v1 (21), *L. japonicus* MG20 v3.0 (22), *P. japonicum* PjScaffold_ver1 (23), or the modified *A. halleri* Ahal v2.2 (24)) using STAR version 2.6.0a. For the *A. halleri* genome, *VIN3*, *FLC*, and *FT* sequences were replaced by those from the sampled population reported previously (25). Alignment used a mismatch allowance of 2 bp. Using both UMI and mapped positions, the deduplication of reads was performed by Umi-Grinder version 0.2.0 with a mismatch allowance of 1 bp for the UMI sequence.

### Data processing of the original TSS-seq, ReCappable-seq and nAnti-CAGE

Raw reads for TSS-seq, ReCappable-seq, and nAnti-CAGE of A549 that were generated by previous studies (26,27) were obtained from the Sequence Read Archive (TSS-seq: DRR095989; ReCappable-seq: SRR9291131 and SRR9291132; and CAGE: SRR9291139 and SRR9291140). After removal of linker sequences from each dataset, the obtained reads were then aligned to rRNA sequences using Bowtie2 version 2.3.5.1 to remove reads derived from rRNA (19). The retained reads were then aligned to the human reference genome hg38 using STAR version 2.6.0a (28) with a mismatch allowance of 2 bp.

### Data processing of 5′ RACE

First, the obtained raw reads were processed by the same pipeline with TSS-seq1 and 2. Then, to remove reads derived from non-specific amplicons, the reads harboring sequence of gene-specific primer were extracted from the reads after deduplication, and 5′ end of the extracted reads was counted.

### TSS clustering from TSS data

Clustering of TSS and the annotation of TSS clusters (TSCs) were performed using TSSr version 0.99.6 (29). Using the getTSS function of TSSr and the default parameters, processed bam files were loaded and TSS counts were estimated. After the normalization of the TSS count as cpm (i.e. Counts Per Million mapped tags), low-support TSSs were filtered out using the Poisson method. From the resulting filtered TSS data, TSCs were called using the clusterTSS function of TSSr with the default parameters. To remove low-confidence TSCs, those TSCs composed of only one read were removed.

### Data analysis of human RNA-seq

Next, we analyzed A549 RNA-seq data obtained in our previous study (26). First, all reads were trimmed using Trim Galore version 0.6.4. The trimmed reads were aligned to the human reference genome hg38 using STAR version 2.6.0a (28) with the ‘–outSAMstrandField intronMotif’ option. The expression levels of each Gencode transcript were then calculated using the expression estimation mode of StringTie version 2.1.5 (30). Expression levels of each Gencode gene were calculated using featureCounts version 1.6.4 (31).

### Performance assessments: TSC detection and threshold determination

To assess the performance of each method with respect to TSC detection and to determine threshold cpm values, we performed accuracy assessments in a similar way as in previous studies (32). Briefly, we classified TSCs into true positives (TPs), false positives (FPs), and false negatives (FNs). TPs were defined as the number of TSCs that overlapped with the TSSs of the Gencode transcript model. FPs were defined as the number of TSCs that do not overlap with the TSSs and ATAC peaks for A549 in a dataset downloaded from the ENCODE website (https://www.encodeproject.org/files/ENCFF876UEM/) (33). FNs were defined as the number of TSSs with expression levels >1 tpm (Transcript Per Million) from the RNA-seq dataset that did not overlap with TSCs but did overlap with ATAC peaks. From these values, we calculated the F1 score for each TSC cpm threshold. The threshold that showed the highest F1 score was used for subsequent analyses.

### Library preparation for plant RNA-seq

Plant RNA-seq libraries were prepared using a TruSeq Stranded RNA Library preparation kit (Illumina) as per the manufacturer's protocol. Prepared libraries were quantified using a DNA 7500 kit and a 2100 Bioanalyzer (Agilent Technologies). 150 bp paired-end sequencing of these libraries was performed on a NovaSeq 6000 (Illumina).

### Analysis of plant RNA-seq data

The RNA-seq reads for the plant samples were first trimmed using fastp version 0.20.0 (34). To remove rRNA reads, trimmed reads were aligned to reference rRNA sequences for each species with Bowtie2 version 2.3.5.1 (19). The retained reads were then aligned to plant genomes (i.e. *N. benthamiana* Nbe.v1 (21), *L. japonicus* MG20 v3.0 (22), *P. japonicum* PjScaffold_ver1 (23) and the modified *A halleri* Ahal v2.2 (24)) using STAR version 2.6.0a (28) with the ‘–outSAMstrandField intronMotif,’ ‘–outFilterMultimapNmax 1,’ and ‘–outFilterMismatchNmax 4″ options. Next, we used StringTie version 2.1.5 (30) to perform transcript annotation for each species. Briefly, aligned reads were assembled into transcripts for each plant species using a guide of the transcript models. For each species, all assembled transcripts and existing transcript models of the same species were merged. Expression levels of each gene were estimated using featureCounts version 1.6.4 (31) and the merged transcript models. Finally, tpm values were calculated using the read counts for each gene.

### Data analysis of plant TSS-seq2

Analysis of TSS clustering was performed using TSSr as mentioned above. After removing low confidence TSCs whose expression levels were below 0.42 cpm, TSCs detected in both duplicates were extracted, and the extracted TSCs were merged using bedtools version 2.29.0 (35).

For the comparison of expression levels with RNA-seq data, the TSS reads aligned to assembled genes from the RNA-seq dataset were counted using featureCounts version 1.6.4. Read counts were then normalized to cpm values.

Next, differential expression analysis between the control and induction of prehaustoria in *P. japonicum* was performed using edgeR version 4.2.1 (36) with classic quantile-adjusted conditional maximum likelihood methods.

## Results and discussion

### Development of TSS-seq2

**Figure 1.**
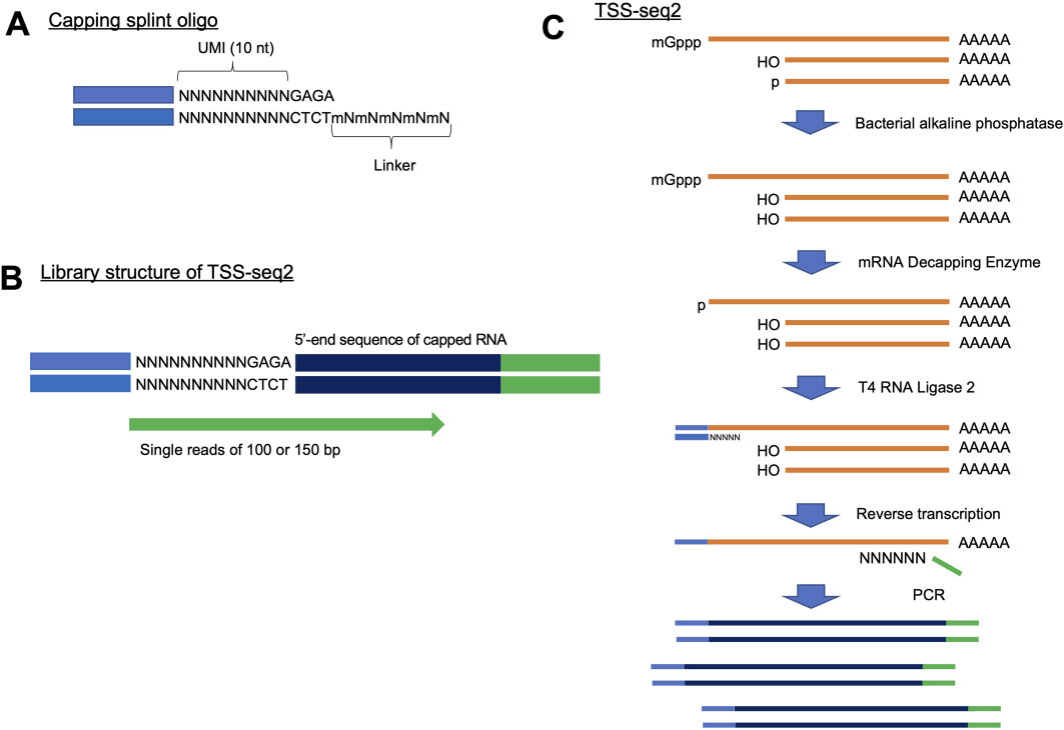
Schematic view of TSS-seq2. Based on an oligo-capping method (13), after dephosphorylation of the 5′ end of RNA with bacterial phosphatase, the cap structures of RNA were removed **(C)**. Capping splint oligos harboring 10 nt of UMI (**A**) were specifically ligated to decapped RNA. After ligation, reverse transcription was performed using a random primer. Sequencing libraries were amplified by primers with a partial sequence of the capping oligo and a random primer. 10 bp of UMIs, 4 bp of linker and 5′ ends of capped RNA were sequenced using single-read sequencing (**B**).

In the previous TSS-seq method, RNA ligation was conducted as a single-strand ligation between a synthetic RNA oligonucleotide and the mRNA. T4 RNA ligase was employed for this ligation (13,14). However, it was noted that the efficiency of single ligation among RNA molecules is poor. It is also been suggested that ligation efficacy is biased between nucleotides at the first base of the mRNA (37,38). To overcome these drawbacks, we employed splint ligation. Recent reports have demonstrated that splint ligation has higher ligation efficacy and lower bias compared to single-strand ligation (17). In the present reaction scheme (Figure 1), the RNA oligo is double-stranded and contains a randomized linker sequence with overhanging oligos (Figure 1A). In the overhanging part, the nucleotides are modified with 2′-*O*-methyl for efficient hybridization with the 5′ end of the mRNA and subsequent ligation. For the ligation, nick ligation implemented by T4 RNA ligase 2 is employed. In addition, to discriminate among PCR duplicates, a UMI sequence was introduced into the RNA oligo sequence (Figure 1A and B). The reaction conditions used for RT were also modified based on other methods for similar TSS detection (9) (see Materials and Methods for details). For example, as for the RTase, we used Maxima H Minus Reverse Transcriptase, since this enzyme has been shown by previous studies to have the highest performance among commercially available enzymes (39,40). Figure 1C shows an overview of TSS-seq2.

### Evaluation of TSS-seq2

Next, to evaluate the performance of TSS-seq2, we constructed a series of libraries using this method. For comparison, we constructed libraries from the same starting material as was used for the original TSS-seq method, which used single-strand ligation. The libraries were constructed from varying amounts of starting material—i.e. 50 ng, 500 ng and 5 μg of total RNA. For evaluation purposes, RNA extracted from the human lung cancer cell line A549 was used for all libraries (Supplementary Figures S1 and S2). Moreover, to achieve a fair comparison, some modifications were made to the method of the original TSS-seq. First, UMI, which was not used there, was introduced to all RNA oligos used. Second, to determine the minimum recruitment of starting material for the original TSS-seq, the ribosomal RNA (rRNA) removal was employed; we termed this procedure ‘TSS-seq1’; see Materials and Methods for details). We found that TSS-seq2 showed an even higher yield of libraries than TSS-seq1 (Supplementary Figures S1 and S2). As the TSS-seq1 uses a single-strand RNA adapter, if reverse transcription may be inhibited (to some extent) by a long RNA–RNA duplex in splint adapter in TSS-seq2, its influence may not be disastrous, at least. Also note that the step of the denaturation of RNA is included for the primer annealing (for 5 min at 65°C, for 2 min at 4°C). From the libraries constructed using the TSS-seq1 and TSS-seq2 methods, 44467513–60959915 and 24172048–35852924 reads were obtained from the Illumina short read sequencer (Supplementary Table S3). It is known that optimal length of library for Illumina sequencer is shorter than 1 kb containing ∼800 bp of insert sequence (41). The library size of TSS-seq2 ranged roughly from 250 bp to 5 kb (Supplementary Figure S2). To assess the effect of the large insert size on the sequencing quality, we verified the sequencing quality and yield of TSS-seq2. We removed the long fragments (over 1 kb) from the TSS-seq2 library (in the case of A549) (Supplementary Figure S3A). The same mean values (35.6) of the base quality score (on the NovaSeq6000) were observed both with and without the upper size removal (Supplementary Figure S3B). Therefore, the long insert size of TSS-seq2 may have a limited (if any) negative effect on the sequencing quality. The yields of reads were almost same with the library without the upper size removal (30 million and 32 million reads for the libraries without and with the upper size selection, respectively, in the case of the 500 ng input library) (Supplementary Figure S3C). Therefore, we decided not to include the step for the upper size selection to the library preparation protocol of TSS-seq2. To remove the bias of sequencing depth, we randomly sampled 20 000 000 raw reads for each dataset. The same numbers of reads were then used for the following analyses (Supplementary Table S4).

We first validated the rate of possible non-specific (i.e. cap-independent) ligation. We assessed the mapping ratio of reads to rRNA, which does not have a cap (42). Without any cap selection procedure, it has been reported that a significant percentage of reads would be assigned to rRNA (9,27,43). While no explicit rRNA depletion method, such as the hybridization-depletion method, was included in the TSS-seq2 procedure, the rRNA reads were almost completely removed from both the TSS-seq1 libraries (i.e. 0.02–0.03% of raw reads after rRNA depletion) and the TSS-seq2 library (i.e. 0.2–0.6% of raw reads) (Supplementary Figure S4 and Supplementary Table S4).

Second, we evaluated the proportion of remaining effective reads after mapping and removing PCR duplicates. This ranged from 8.5%–64% and 23%–60% of raw reads, depending on the amount of the starting material, for the TSS-seq1 and the TSS-seq2 libraries, respectively (for more details, see Supplementary Figure S4 and Supplementary Table S4). Although the RNA sources and the sequencing depth were different, the number of valid reads after the removal of PCR duplicates showed that the effective yield was higher for TSS-seq2 (i.e. 4547349–5168707 reads obtained by starting with 50 ng of total RNA) than for any other TSS detection method, including LQ-ssCAGE (i.e. 575008 reads starting from 50 ng of total RNA) and STRIPE-seq (i.e. 686981–806174 reads starting from 100 ng of total RNA), as published in the respective papers (7,9). The obtained data of TSS-seq2 using large inputs (5 μg–500 ng) contained ∼20% of the PCR duplicates for most of the cases sampled (Supplementary Figure S4 and Supplementary Table S4). However, the PCR duplicates could be discriminated by utilizing the UMIs. Furthermore, note that the PCR duplicates can also form during the sequencing step (44). The patterned flow cells were used for sequencing the libraries with Illumina NovaSeq6000. It is known that some of the DNA fragments captured in a well of the patterned flow cell can be, by chance, copied to adjacent wells during cluster generation, though usually at a low frequency. However, the rate is increased when the input amount of the library becomes low. (Those duplicates are called ‘optical duplicates’ or ‘exclusion amplification’ (ExAmp) duplicates). Although duplicates of this type should also have been generated even via a PCR-free method such as CAGE, it is often ignored when UMI separation is not possible. In more detail, when we evaluated the rate of optical duplicates for the TSS-seq2 data of *P. japonicum* (which is a pair-end dataset; for more details, also see the Application of TSS-seq2 for TSC detection in four plant species section) using MarkDuplicates, the rate of the optical duplicates was 8% (Supplementary Figure S5A). To verify the duplicates that do not rely on PCR, we checked the alignment statistics of a demo dataset, consisting of 16 PCR-free whole-genome sequencing (WGS) libraries. These libraries were prepared from human DNA sample NA12878 using the Illumina DNA PCR-Free Prep Kit and sequenced by NovaSeq6000 sequencer. In this dataset, the proportion of optical duplicates was ∼9%, which is similar to that in the TSS-seq2 dataset (∼8%) (Supplementary Figure S5B).

**Figure 2.**
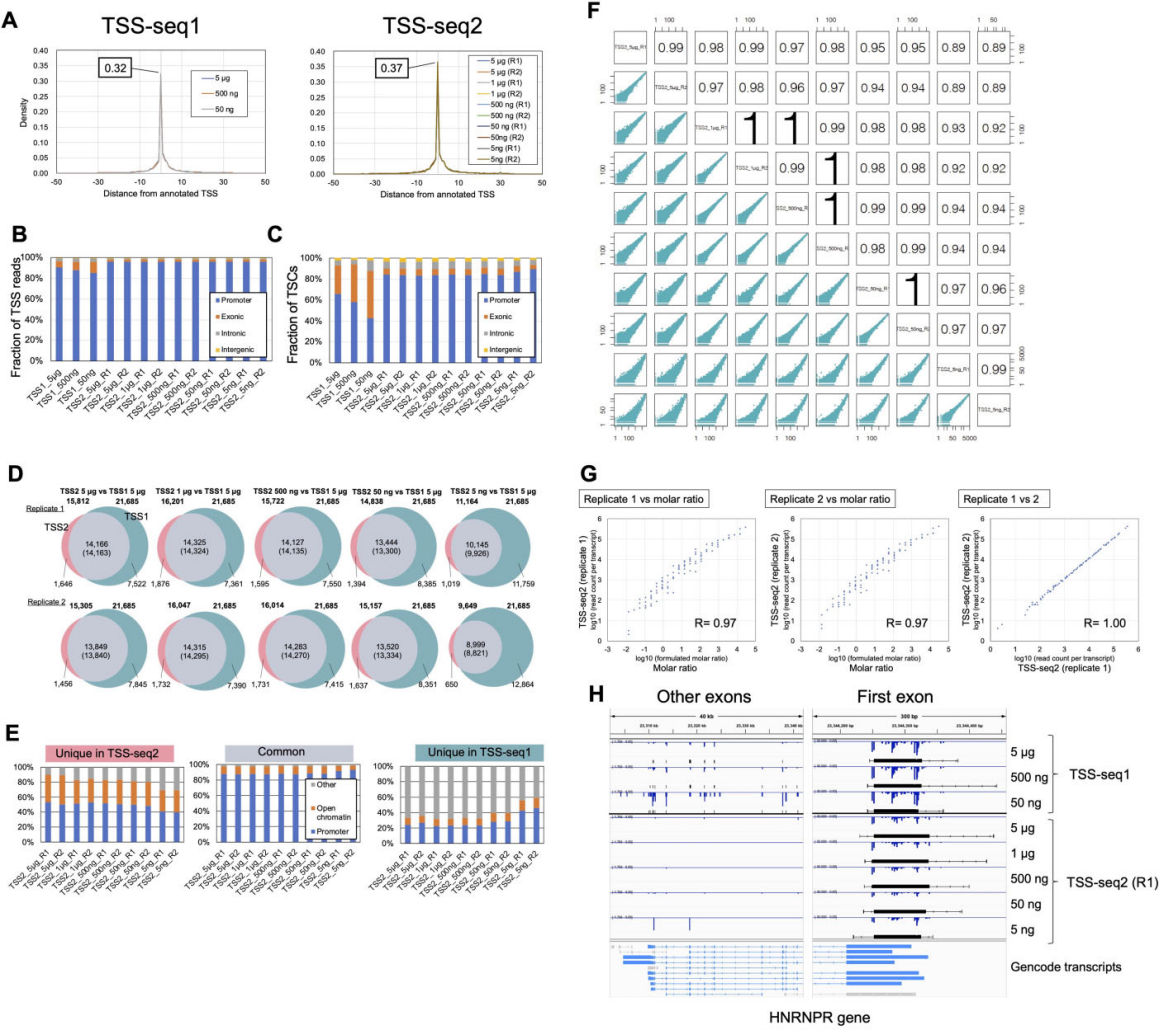
Comparison of TSS-seq1 and TSS-seq2. (**A**) Densities of 5′ ends of mapped positions of TSS-seq1 and TSS-seq2 reads for the TSS of Gencode v40. (B and C) Breakdown of 5′ ends of mapped positions of TSS reads (**B**) and TSS clusters (TSCs) detected by TSSr (29) with ≥1 count per million (cpm) and ≥2 reads (**C**) overlapping with the promoter (i.e. ±100 bp of TSSs), exon, intron, and intergenic regions of Gencode transcripts. (**D**) Overlap between TSCs detected by each replicate of TSS-seq2 prepared from 5 μg, 1 μg, 500, 50 and 5 ng total RNA of A549 (left pink circles) and those detected by TSS-seq1 prepared from 5 μg of the same RNA (right dark cyan circles). Numbers of TSCs uniquely detected by TSS-seq2 or TSS-seq1 and those of TSS-seq2 overlapping with those of TSS-seq1 are shown, respectively. The numbers of TSCs detected with TSS-seq1 overlapping with those of TSS-seq2 are shown in parentheses. Each group of TSCs was extracted using the intersect function of bedtools (35). The total number of TSCs for each dataset is shown above each Venn diagram. Overlap was defined as overlapping by ≥1 bp. Most TSCs span tens to hundreds of base pairs. Therefore, when TSCs were compared between different datasets, a TSC of a given dataset occasionally includes several TSCs of other datasets. The numbers of ‘common’ TSCs between TSS-seq1 and 2 differ from each other. **(E)** Breakdown of TSCs uniquely detected in TSS-seq2 (5 ng–5 μg) or TSS-seq1 (5 μg) and those commonly detected by both methods overlapping with promoters (i.e. ±100 bp of Gencode TSS) and open chromatin regions (i.e. ATAC peaks of A549 downloaded from ENCODE) that specifically exclude promoters. TSCs commonly detected by both methods based on the TSCs of TSS-seq2. Overlap was defined as overlapping by ≥1 bp. (**F**) Scatterplots of counts of TSS reads (TSS counts) for TSS-seq2 datasets. Pearson correlations are shown for all graphs. These plots were prepared using the plotCorrelation function of TSSr. We first counted the first base positions (their genomic coordinates) of the TSS reads (note that the count numbers were not summed to the gene level but were counted at each base). Then, Pearson's correlation coefficient for the counts at a given TSS position between two datasets was calculated. More precisely, we employed ‘TSSr,’ a public tool, for the analysis of TSS data. Following the standard procedure of TSSr, we extracted the count of the 1st bp position of the TSS reads from BAM files using the getTSS function with default parameters, and the counts were used as input data using the plotCorrelation functions with default parameters per the instructions written on the website of TSSr (https://github.com/Linlab-slu/TSSr/tree/master/R). When the reads were loaded by the getTSS function with the default parameter, low-quality reads were removed by thresholds for average quality of sequencing (≥10 in the Phred score) and mapping (≥20 in the MAPQ score). In the plotCorrelation function, Pearson's correlation coefficients between datasets for the counts of the 1^st^ base position of TSS reads without any filtering, except for the read qualities and normalization, and the estimated coefficients are rounded to two digits. As for the plot, data points with zero values are not displayed because the logarithmic axis is used for the function. (**G**) Scatter plots of the read count of TSS-seq2 and the molar ratio of ERCC RNA Spike-In Mix (see also the Materials and methods section for details of preparation and data processing). After the ERCC RNAs were ‘capped’ using the Vaccinia Capping System, using the obtained 5 ng of the capped ERCC RNA, the TSS-seq2 libraries in duplicate were constructed. After the detection and extraction of UMIs with UMItools, the reads were aligned to the sequences of ERCC transcripts. After deduplication with the UMI grinder, the TSS reads that were aligned to the plus strand of each RNA species were counted. The read counts and molar rates obtained from the manufacturer's website (https://assets.thermofisher.com/TFS-Assets/LSG/manuals/cms_095046.txt) were normalized to log_10_. Pearson's correlation coefficients are shown in the plots. (**H**) Typical view of the distribution of 5′ ends of mapped positions of TSS reads and TSCs around the *HNRNPR* gene. Blue bars indicate expression values (cpm) of TSS per position, and cpm of the minus strand is shown as a negative value. TSCs detected by TSSr with ≥1 cpm and ≥2 reads shown as black boxes.

Third, to obtain precise positional information for all TSSs, the genomic coordinates of the mapped position of each read were examined. In this study, we defined transcription start site (TSS) as the mapped 5′ end position of an individual TSS read. Their genome-wide distribution patterns were compared with the annotated TSS positions obtained from Gencode. The 5′ ends of TSS reads obtained by both TSS-seq1 and TSS-seq2 were predominantly located around annotated TSSs (Figure 2A and Supplementary Figure S6). In Figure 2A, TSS-seq2 showed a higher density of 5′ ends of the TSS reads (∼0.37) exactly overpaying (at the 1 bp position of) the annotated TSSs rather than the case of TSS-seq1 (∼0.32). A significant majority of the 5′ ends of TSS-seq1 reads (i.e. 85%–91%) resided within the core promoter region (±100 bp around the TSSs of Gencode transcripts), and even higher rate of the 5′ ends of TSS-seq2 reads (96%) resided within the region (Figure 2B). To further validate the detection of TSSs with greater precision, and for the sake of promoter detection in an *ab initio* manner, the detected TSSs were first clustered using TSSr software (29). Transcriptional initiation can occur from a certain range of genomic regions, rather than strictly from a single base (4,45). TSS cluster (TSC) is a region consisting of several TSSs defined by the clustering of individual TSS. With the standard data analysis for TSS detection, TSCs are filtered by tag-counts or/and normalized expression level for each to remove technical errors or sporadic transcription noise (4). The clustering was made when the TSS should not be separated by >100 bp. We have filtered TSCs with a relatively conservative threshold of ≥ 1 cpm and ≥ 2 reads per TSC. A total of 21685–33465 and 14838–16201 TSCs were detected in the TSS-seq1 and TSS-seq2 datasets, respectively (Supplementary Table S4). These TSCs were then compared to the positions of the annotated promoters. As a result, 83–85% of TSCs that were detected by TSS-seq2 overlapped the core promoter regions. This frequency was higher than that of the TSCs of TSS-seq1 (43%–66%) (Figure 2C). In fact, 89%–91% of the TSCs detected by TSS-seq2 were included among those detected by TSS-seq1 using a 5 μg input (Figure 2D). On the other hand, larger number of the TSCs that were uniquely detected by TSS-seq1 (7361–12864) were detected than those uniquely detected by TSS-seq2 (1394–1876). The TSCs uniquely detected by each methods showed lower expression levels than the common TSCs (Supplementary Figure S7). Although there is the remaining possibility that minor TSCs may also represent genuine TSCs, it is more likely that they represent errors. In Figure 2E, the TSCs uniquely or commonly detected by either method were considered to determine whether they overlapped the promoter regions (i.e. ±100 bp of TSSs) of Gencode transcripts or open chromatin regions (i.e. ATAC peaks of A549, downloaded from ENCODE). In comparison of TSS-seq2 prepared from 50 ng–5 μg total RNA with TSS-seq1 from 5 μg total RNA, while only 32–40% of TSS-seq1-unique TSCs overlapped with the promoter regions or open chromatin regions of Gencode/ENCODE, 80%–90% of TSS-seq2-unique TSCs overlapped them. Even starting from 5 ng of total RNA, TSS-seq2 shows a higher overlapping rate with these regions (56%–59% in TSS-seq1-unique TSCs and 69% in TSS-seq2-unique TSCs; for more details, also see next section).

Fourth, to evaluate the reproducibility of TSS-seq2, we calculated Pearson's correlations for counts of TSS per position (TSS counts) among replicates or using different input amounts. We obtained high correlations (*R* = 0.94–1.00) using TSS-seq2 for preparations from different conditions (50 ng–5 μg) (Figure 2F). In particular, we observed that the correlations between the technical replicates of TSS-seq2 prepared from the same RNA samples were extremely high (*R* = 0.99–1.00) (see also Supplementary Figure S8 for the results of TSS-seq1). With respect to precise assessments of expression levels, TSS-seq2 results were compared with RNA-seq data and were found to have a positive correlation (*R* = 0.82–0.84) (Supplementary Figure S9). Furthermore, to evaluate quantitative measurements of TSS-seq2, we performed the TSS-seq2 analysis using the ERCC RNA Spike-In Mix containing preformulated 92 transcripts. Before its use, the ERCC RNAs were ‘capped’ using the Vaccinia Capping System. Using the obtained 5 ng of the capped ERCC RNA, the TSS-seq2 libraries in duplicate were constructed. The TSS reads which were aligned to each ERCC RNA species were counted. The observed read counts and expected read counts based on the molar rates obtained from the manufacturer's website were compared (Figure 2G). A very strong correlation (*R* = 0.97) was observed. The correlation coefficient between the replicates was R = 1.00, indicating a highly quantitative measurement and the reproducibility of TSS-seq2.

**Figure 3.**
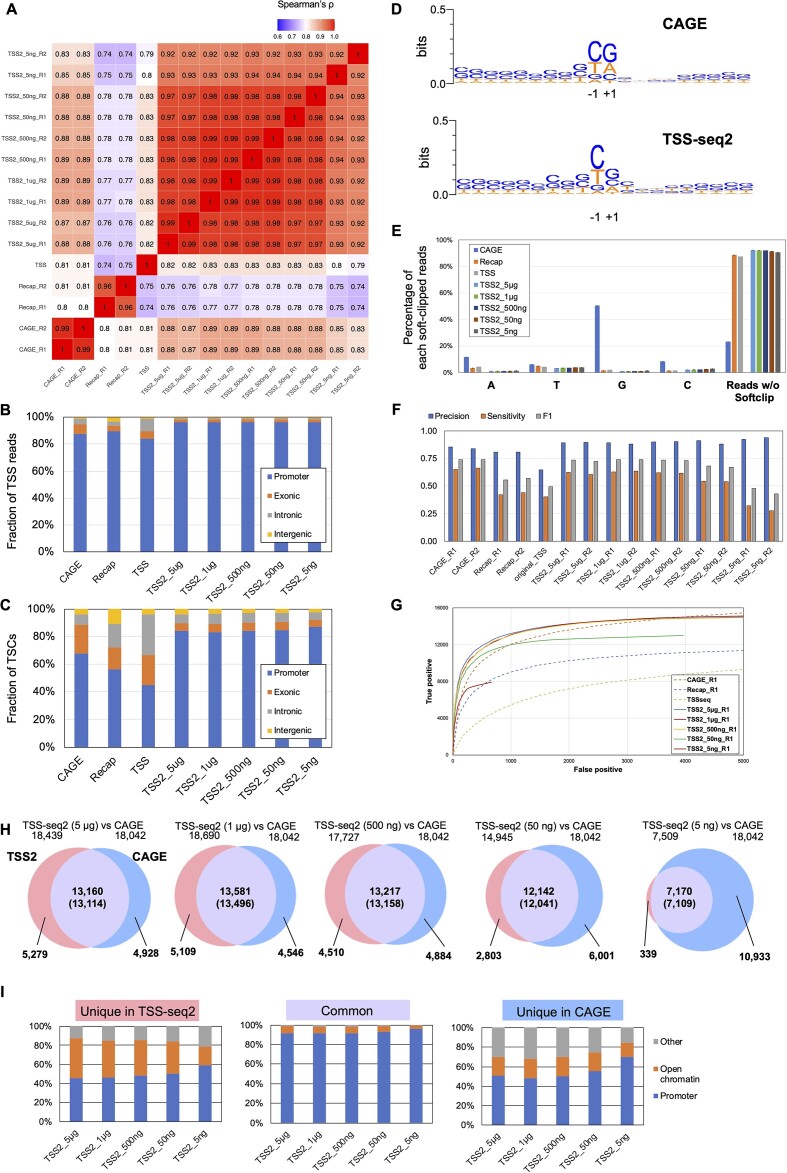
Comparison with existing methods. (**A**) Heatmap of Spearman correlations among nAnTi-CAGE (CAGE) (5,27), ReCappable-seq (Recap) (27), the original TSS-seq (TSS) (26) and different input amounts in TSS-seq2 (TSS2). Estimation of TSS counts within the promoter (i.e. ±100 bp of TSSs) of Genocode transcripts. After normalization using ‘log_10_ (cpm + 1),’ Spearman rank correlation coefficients were calculated using all combinations. More specifically, for comparing TSCs between different datasets, practically, some datasets from noisy methods or noisy conditions gave a large number of false-positive TSCs if constructed by their own, which has made the comparison difficult. Therefore, we estimated the sums of TSS counts that fell within each Gencode promoter (±100 bp from TSS). Then, the counts were normalized to log_10_ (cpm + 1). Spearman's rank correlation coefficients were calculated using the normalized values for all combinations. (B and C) Breakdown of 5′ ends of mapped positions of TSS reads (**B**) and TSCs detected by TSSr (29) with ≥1 cpm and ≥2 reads (**C**) overlapping ≥1 bp with the promoter (±100 bp of TSSs), exon, intron and intergenic regions of Gencode transcripts. Average rates of replicates are shown except for the original TSS-seq, for which no replicate is available. Note: (B) and (C) represent a read-based feature, rather than a gene-based feature. As far as the read-based features were concerned, all TSS detection methods were similar to each other, reflecting the emphasized influence of highly expressed genes. As the expression levels of TSCs varied widely, a large fraction of the reads accounted for the TSCs of the highly expressed genes. Among the TSCs of low expression levels, miscalled TSCs, which were derived from technical errors or noise level transcriptions, are more prominent. Also, this population is larger when all the TSCs are counted without any filtration. To clearly show this tendency, we have shown the expression levels of TSCs detected with promoter regions and non-promoter regions (Supplementary Figure S18). (**D**) Sequence consensus within ±10 bp from mapped positions of 5′ ends of TSS reads of CAGE (replicate 1) and TSS-seq2 (5 μg input, Replicate 1). Sequence logos were generated using weblogo version 3.7.9 (62). (**E**) Composition of soft-clipped bases adjacent to the 5′ end of the mapped portion of the read. Reads without soft-clipping and average rates of replicates are shown in the plot. Error bars represent standard errors between replicates. (F and G) Performance assessment of TSC detection (see the Materials and methods section for details). (**F**) Precision, sensitivity and F1 scores for each method for TSC detection. (**G**) ROC curves for each method. (**H**) Overlap between TSCs detected in both replicates of TSS-seq2 prepared from 5 μg, 1 μg, 500, 50 and 5 ng and those detected in both CAGE replicates (27). From TSCs are detected by TSSr (29) with the parameter showing the highest F1 score (Supplementary Table S5), those detected in both replicates were extracted and merged using the intersect and the merge function of bedtools (35) (Supplementary Figure S23). Numbers of TSCs uniquely detected by TSS-seq2 or CAGE, and those of TSS-seq2 that overlapped with those of CAGE are shown, respectively. The numbers of TSCs of CAGE overlapping with those of TSS-seq2 are shown in parentheses. The total numbers of TSCs for each dataset are also shown above each Venn diagram. Overlap was defined as overlapping by ≥1 bp. Most of TSCs span tens to hundreds of base pairs. Therefore, when the TSCs were compared between different datasets, a TSC of a given dataset occasionally includes several TSCs of other datasets. The numbers of ‘common’ TSCs between TSS-seq2 and CAGE differ from each other. The TSCs used were filtered using a different threshold from those in Figure 2D, Figure 3C, and so on. The optimal threshold for filtering depends on method or data. For the first evaluation, we employed a ‘common’ threshold for all datasets. For example, ∼25 000 and ∼16 000 the TSCs were detected by TSS-seq1 and TSS-seq2 (50 ng–5 μg) using a threshold of ≥1 cpm and ≥2 read per TSC (Supplementary Table S4). With this threshold, 68% and 84% of CAGE and TSS-seq2 TSCs overlapped with the promoters (Figure 3C), suggesting that CAGE data is ‘noisier’ than TSS-seq2 at this (common) threshold. Then, we set the threshold in a more statistically relevant manner, the one giving the highest F1 score (Supplementary Table S5) to apply the new method to the analysis of hitherto uncharacterized plants species. At this threshold, the CAGE data, which was ‘noisier’ at the ‘common’ threshold became less so. Moreover, the overlapping rate of TSCs between CAGE and the TSS-seq2 datasets became higher than that when filtering at ≥1 cpm and ≥2 read per TSC (Supplementary Figure S24). (**I**) Breakdown of TSCs uniquely detected by CAGE or TSS-seq2 and those commonly detected by both methods (**H**) by overlapping with promoters (i.e. ±100 bp of Gencode TSS) and open chromatin regions (i.e. ATAC peaks of A549 downloaded from ENCODE) specifically excluding promoters. TSCs commonly detected by both methods based on the TSCs of TSS-seq2. Overlap was defined as overlapping by ≥1 bp. ‘Common’ TSCs were mostly those of canonical Gencode promoters. Both ‘CAGE-unique’ or ‘TSS-seq2-unique’ populations include a significant population of transcripts from ‘open chromatin regions,’ suggesting that they represent non-protein coding transcripts. Further, note the larger population of ‘others’ (transcripts of non-open chromatin regions; gray parts) in the ‘unique in CAGE’ population.

Finally, to demonstrate the high specificity of TSS-seq2 more directly, we prepared the TSS-seq2 libraries also from the total RNAs that were fragmented using the NEBNext Magnesium RNA Fragmentation Module under two treatment conditions (1 or 2 minutes) (Supplementary Figure S10A and B). Although the obtained data from TSS-seq2 of the fragmented RNA (DV200: 82% and 65%, respectively) showed to some extent a lower rate of the 5′ ends of TSS-seq2 reads (∼90%) resided within the promoter region compared to that constructed from high-quality RNA (RIN = ∼10) (Supplementary Figure S10C and Figure 2B). Nevertheless, these rates were still comparable with the ones prepared from the intact RNA using other methods (see also the Comparison with other methods section and Figure 3B). Moreover, those libraries showed high Pearson's correlations (*R* = 0.94–0.97) with the TSS-seq2 library from high-quality RNA (Supplementary Figure S10D). The effects of UMI sequence and the NNNNNN sequence of the splint adapter were also evaluated in Supplementary Figures S11 and S12.

These results collectively indicate that TSS-seq2 outperforms the previously published TSS-seq1 method. It is also important to note that this accuracy was achieved regardless of the amount of the starting RNA; good results were obtained even with as little as 5 ng (see next section). Figure 2H exemplifies TSS detection using the *HNRNPR* gene as an example (see Supplementary Figure S13 for other examples).

### TSS-seq2 analysis from a small amount of RNA

We attempted to perform TSS-seq2 from 5 ng of total RNA, which is the smallest input amount of total RNA that has been used to analyze mammalian genomes using the TSS detection method, with the notable exception of template switch-based methods such as STRT-seq and Smar2C2 (6,10,46). We prepared TSS-seq2 libraries from 5 ng of A549 total RNA twice and randomly sampled 20000000 reads from 71097269–83705632 total raw reads (Supplementary Figure S2 and Supplementary Table S3). The number of valid reads itself in 5 ng input (426414–609002) was significantly smaller than that obtained with 50 ng–5 μg inputs (4547349–11963596) (Supplementary Figure S4 and Supplementary Table S4). This is because the 5 ng input showed a higher PCR duplicate rate among the mapped reads (70%) than other inputs (Supplementary Figure S14). The valid sequencing ratio almost reached saturation in the 5 ng input. The number of valid reads depends on the number of RNA molecules for which the 5′-cap structure is successfully converted. Therefore, due to the limitation in the number of valid RNA molecules, the number of the detected TSSs may have started to decrease at this input. Although the number of valid reads in TSS-seq2 (5 ng) may be smaller than with higher starting amounts (50 ng–5 μg), those numbers were comparable to those obtained with ssCAGE (575008 reads) and STRIPE-seq (686981–806174 reads) using 50 and 100 ng, respectively (7,9). However, even in 5ng input for the TSS-seq2 data, 96% of these reads aligned to core promoter regions (Figure 2A and B). Therefore, we confirmed that the accuracy of TSS detection per read level was almost the same regardless of input amount. 9650–11165 of the TSCs were called by TSSr, and 87%–89% of these were found to overlap with core promoter regions (Supplementary Table S4 and Figure 2C). However, the sensitivity of TSC detection in the 5 ng input was lower than with higher inputs (Supplementary Table S4 and Supplementary Figure S15A). In Figure 2C and D, we filtered TSCs with a relatively conservative threshold of ≥ 1 cpm and ≥ 2 reads per TSC. Accordingly, the number of TSCs remaining after filtering in the 5 ng input dataset (9649–11164) was lower than that obtained when starting from a higher input (15157–16201). In fact, the TSCs not detected in the 5 ng input but in the 5 μg input were those of weekly expressed TSCs (Supplementary Figure S15B), which could be rescued by loosening the threshold. Moreover, TSS counts of the 5 ng input showed a strong correlation with those of higher input amounts (i.e. *R* = 0.89–0.97), and the correlations between replicates prepared from 5 ng input material was very high (*R* = 0.99) (Figure 2F). To quantitatively assess TSS-seq2 detection without relying on clustering, we estimated the expression levels of each annotated TSS in Figure 3A. We observed a high correlation between the wide-range input amounts, including the 5 ng input. Therefore, TSS-seq2 can serve for precise TSS quantification. Even for the 5 ng input, the precision was maintained, even if the detection, judging from the PCR duplicate reads, is reaching a plateau (Supplementary Figure S14). Therefore, due to the lower input amount, the number of the valid reads and the number of called TSCs were lower than the numbers produced by analyses of larger input amounts. However, the specificity and reproducibility of TSS-seq2 was maintained even when using only 5 ng of input.

The portion of mapped reads and valid reads from total reads was low in libraries prepared from small input amounts (5 and 50 ng) (Supplementary Figure S4). In libraries produced from 5 and 50 ng input material, DNA fragments shorter than 200 bp were less abundant than they were in libraries produced from 500 ng–5 μg input material (Supplementary Figure S2). By removing fragments without inserting RNA sequences carefully, the portion of mappable reads should increase. We attempted to reduce the rate of ‘invalid’ reads. We noticed that most of the invalid reads (except for rRNA reads and PCR duplicates) were products of the reverse-transcribed capping oligo, which does not have any insert sequence. In fact, short DNA fragments (100–250 bp), which are suspected to be these by-products, were already detected at quality checks of the library using Bioanalyzer (Supplementary Figure S16). To remove this fraction, we performed the size-selection step and re-sequenced the TSS-seq2 libraries of 5 ng and 50 ng inputs. The rate of invalid reads was significantly reduced by this procedure (Supplementary Figure S4 and Supplementary Table S4). Consequently, the number of valid reads, starting from a total of 20 million raw reads for each, increased from 5 million to 7 million and from 0.5 million to 1 million for the 50 ng and 5 ng input libraries, respectively (Supplementary Figure S17). When all raw reads were used (51980436 for the 50 ng input and 65272849 for the 5 ng input), the number of valid reads increased to 10 million and to 1.4 million, respectively. Even though we could successfully improve the yield, we also noticed that the sequencing is already saturated at 20 million reads for the 5 ng input library. As the 5 ng of material RNA roughly correspond to 500 cells, the complexity of the mRNAs within that cellular population, which could be represented by this method, may have already come to the plateau. Because TSS-seq2 does not use a carrier substance, which is used to reduce loss during the library preparation process, these numbers could be increased somewhat by employing a carrier (6). The datasets of 50 and 5 ng without removal of short fragments with replicates were used for the following analyses.

### Comparison with other methods

To further evaluate the performance of TSS-seq2 against other TSS detection methods, we obtained public data for A549 cells prepared by nAnti-CAGE, one of the most frequently used forms of CAGE (27). Although the CAGE method was the most popular method used for TSS detection, we also considered data from the original TSS-seq (26) and ReCappable-seq (27) methods, as they are similar methods, both consisting of a series of enzymatic reactions. In the original version, TSS-seq, the reaction started from 50 μg of total RNA and old chemistry such as single-strand ligation of capping oligo without UMI was employed (13). Precisely, for comparison, we employed the data obtained in our previous study (26). From the sequence datasets, 20 million raw reads were randomly selected for each library, and these reads were used for the following analyses (Supplementary Table S4).

As for the above comparison with TSS-seq1, we first evaluated the proportion of rRNA reads. As indicated previously (27,32), CAGE showed a high proportion (33%) of reads derived from rRNA. As a result, the rates of valid reads became comparable (59%) with those of TSS-seq2 (54%–60% for 500 ng–5 μg input) after removing PCR duplicates, even though the CAGE method does not include any PCR step (Supplementary Figure S4). Estimated TSS expression levels obtained from the Genocode annotation were also compared between TSS-seq2 and CAGE. Regardless of the input amount of total RNA, expression levels were highly correlated between TSS-seq2 (even with 5 ng) and CAGE (Spearman's ρ = 0.83–0.88), compared with other methods (ρ = 0.79–0.80), suggesting that expression levels are precisely represented by both methods (Figure 3A). The accuracy of the detection of individual TSSs and deduced TSCs was also compared against the Genocode annotation (Figure 3B, C, and Supplementary Figure S18). For the TSC detection by TSSr (29), the same threshold (≥1 cpm and ≥ 2 read) was employed for both methods. We found that TSS-seq2 showed higher performance in the concordance rate of mapped positions of 5′ ends of reads and TSCs with the Gencode promoters (i.e. 96% and 84–89%, respectively) relative to the other methods: CAGE (i.e. 88% and 68%), Recap (i.e. 89%, 58%), TSS-seq (i.e. 84%, 45%). To validate the TSCs detected only in TSS-seq2, we performed the 5′ RACE regarding the TSCs of two genes (*THSD7A* and *UNC13D*), which were detected by only TSS-seq2 (Supplementary Figure S19). In previous studies, including the ReCappable-seq paper, 5′ RACE was performed by the method utilizing the oligo-capping (27,47,48). We performed the 5′ RACE by amplifying the 5′ terminal of RNA using a gene-specific primer and primer hybridizing capping oligo sequence. We found that, for both cases, the result of the TSS-seq2 were confirmed by individual 5′ RACE.

One of the concerns for TSS-seq was that base bias was introduced at the RNA ligation step. To confirm that ligation bias was improved in TSS-seq2, the sequence consensus of TSSs detected by each method was generated (Figure 3D and Supplementary Figure S20). We found that the first base was not relevantly biased to A and T, and to a low rate of G, which has been suggested to be the preferred base for the original RNA ligations (49). A comparison of the consensus sequence with CAGE results also showed that the consensus patterns were generally similar. However, CAGE showed some preference for G and A at the + 1 position of the TSS. This G and A bias by CAGE may be derived from the terminal deoxynucleotidyl transferase (TdT) activity of reverse transcriptase, which mainly adds C or T at low frequencies to the end of the first-strand cDNA (9). In fact, the overrepresentation of G and A was more often at the soft-clipped base adjacent to the mapped 5′ end; this may result in the introduction of bias (Figure 3E). The percentage of valid reads without soft clipped bases, which shows that the 5′-ends of reads are mapped to the genome from the first base, is also shown (the rightmost group of the bars in Figure 3E). TSS-seq2 shows a significantly higher rate of the reads without soft clipped bases. Thus, TSS-seq2 is not affected by artificial base addition by RTase. It is true that the true figure of the precise TSS consensus remains elusive even for the most well-annotated human genome; thus, an absolute evaluation is not possible. However, it is possible that TSS-seq2 may more precisely represent the consensus of TSS at a single base resolution. This accuracy is due to the specificity of splint ligation and the fact that it is not affected by TdT activity.

Because both TSS-seq2 and ReCappable-seq can detect TSSs at a 1 bp resolution, we compared TSSs at the resolution between TSS-seq2 (TSS2; 5 μg, replicate 1) and ReCappable-seq (Recap; 5 μg, replicate 1). A total of 93271 and 72968 individual TSS positions (1 bp positions) whose expression levels were > 1 cpm were detected in TSS-seq2 and Recappable-seq, respectively. Among them, 27344 TSSs were detected by both methods (Supplementary Figure S21). Among them, 43265 and 17992 TSSs were TSS2-unique and Recap-unique (that were not detected by the other method with even 1 read), respectively. For these ‘unique’ TSSs, firstly, the TSS positions were compared with promoters of Gencode TSSs (within 100bp from the Genocode TSSs: Supplementary Figure S21A). We found that 94% (40497) of the TSS2-unique TSSs and 98% (26890) of the ‘common’ TSSs overlapped those promoter regions. On the other hand, only 59% (10685) of the Recap-unique TSSs were found within those promoter regions. We further examined whether those TSSs are supported by at least one CAGE read (from the 5′-mapped ends of CAGE reads with < 5 bp allowance; note that the shift of the TSS position was expected by the addition of extra bases due to TdT activity of the RT enzyme used in CAGE) (Supplementary Figure S21B). We found that 100% (27320), 95% (41281), and 72% (12989) of the common, TSS2-unique, the Recap-unique TSSs, respectively, overlapped CAGE reads. Furthermore, we also found that, whereas the TSSs of TSS-seq2 and CAGE showed similar distributions, the TSSs detected by ReCappable-seq showed a different distribution, even for biologically relevant genes in cancer cells like A549, such as *KRAS* and *MYC* (Supplementary Figure S21C). We also examined the consensus motifs around the TSSs for these three groups. We found that the TSS2-unique and the common TSSs showed a strong C/T motif at the -1 base position of the TSSs (Supplementary Figure S21D). This motif is less relevant for the Recap-unique TSSs. Instead, the A/T (U in RNA) at + 1 position was observed. It is indicated that, in the ligation by T4 RNA ligase, U and A are preferred at the + 1 position (49). ReCappable-seq employs very complicated procedures, including two steps of the ‘decapping’ and ‘ligation’ reactions, and so on (27). A relevant population of the Recap-unique TSSs may reflect some bias from those reactions.

As for detection of TSSs, we have estimated the precision of each method on various criteria, similar to the ReCappable-seq paper (27). First, we extracted TSSs (positions) whose expression levels were > 1 cpm for each method. The TSSs were classified as true positives (TPs) and false positives (FPs) following various criteria, such as overlap with the open chromatin regions detected by ATAC-seq, the core promoter regions (±100 bp of Gencode TSSs), or overlap with the 5′ end mapped positions of at least one CAGE (an allowance of <5 bp was made) (Supplementary Figure S22). Among the criteria, TSS-seq2 showed the highest precision scores compared with other methods.

To more strictly evaluate the performance with respect to TSC detection, we determined the cpm threshold of the TSCs with the highest F1 scores, according to the results of a previous study (32) (see the Materials and Methods section for details). At a high input level (i.e. 500 ng–5 μg), TSS-seq2 data showed the best performance for all scores, including precision (0.89–0.90), sensitivity (0.76–0.79) and F1 score (0.82–0.83), compared to other methods (Figure 3F and Supplementary Table S5). At a lower input level (i.e. 5–50 ng), TSS-seq2 data showed the highest precision (0.91–0.96), but its sensitivity (0.42–0.48 and 0.70–0.71 in 5 and 50 ng, respectively) was reduced. For further evaluation of the sensitivity and the selectivity, we drew an ROC (Receiver Operating Characteristic) curve for each method (Figure 3G). The ROC curve analysis also confirmed the overall high precision of TSS-seq2. To identify the detected TSCs with the highest F1 scores in the TSS-seq2 dataset, we compared the TSCs commonly detected among the replicates of TSS-seq2 (Supplementary Figure S23) with those of CAGE (Figure 3H). The majority of the TSCs of TSS-seq2 (69–96%) overlapped with the TSCs identified by CAGE. ∼70% of the TSCs detected by CAGE (5 μg) were covered by the TSCs of TSS-seq2 from higher inputs (50 ng–5 μg), except for those of the 5-ng input (<50%). As for the 5-ng input, considering the quite high overlapping rate with CAGE (96%), highly confident TSCs were detected in the 5-ng input with a high probability, though the number of TSCs is smaller because of the lower sensitivity caused by the lower number of valid reads (Supplementary Table S4 and Figure 3H). The TSCs of TSS-seq2 showed a high degree of overlap between replicates, except for 5 ng (Supplementary Figure S23), similar to the comparison between TSS-seq2 and CAGE (Figure 3H). A fewer number of TSCs in 5 ng derived from a fewer number of valid reads after removing PCR duplicates. The minor TSCs with lower expression levels may have been filtered out in the 5 ng dataset at the given threshold (Supplementary Table S4 and Supplementary Figure S25). As for the TSCs uniquely detected by TSS-seq2, 77%–87% of these overlapped the promoter regions or open chromatin regions (50), suggesting they may represent genuine uncharacterized TSCs (Figure 3I).

One of our aims in developing TSS-seq2 was to establish a method that will be more user-friendly than previous ones. The library preparation of TSS-seq2 takes less than 7–8.25 h (Supplementary Figure S26), which is the shortest duration among all the TSS detection methods to the best of our knowledge, except for a template switching-based method STRIPE-seq (∼5 h) that may not strictly be regarded as a cap replacement method (9). For instance, the original versions of TSS-seq and CAGE take 41 h and 62 h, respectively, according to previous study (32). We also estimated the cost for the library construction for TSS-seq2 based on the US price of each reagent, resulting in a total cost of 66 USD (Supplementary Table S6). The cost of TSS-seq2 was similar to or slightly cheaper than other methods, except for the simple template switch-based methods (for example, the costs of the library preparations for CAGE and the original TSS-seq are 81 and 315 USD, respectively (32). This is partly due to the simplified library preparation protocol (Supplementary Figure S26). Smar2C2 is a method based on template-switch reaction (10). The protocol is simple, and its minimum input requirement is very low (40 pg of RNA for Soybean) compared to other template switch-based methods. However, its specificity in TSS detection is also lower than the level of CAGE (thus, TSS-seq2), as indicated in Smar2C2 paper (10). In fact, the TSS position as detected by Smar2C2 is distributed over a wider region compared to CAGE. Also, because Smar2C2 depends on the TdT activity of the RT enzyme, Smar2C2 cannot determine TSS positions at a single base resolution. Conversely, as a relatively rough TSS detection method, the advantage of Smar2C2 is the time required for library preparation (10 h), which is in the same range as TSS-seq2 (7–8.25 h).

Taken together, we conclude that TSS-seq2 shows equivalent or even superior performance when compared to alternate representative TSS detection methods.

### Application of TSS-seq2 for TSC detection in four plant species

Next, we demonstrated the application of TSS-seq2 for detecting TSSs in unannotated species. For this purpose, we selected several plant species with a broad range of characteristic features: *N. benthamiana*, *L. japonicus*, *P. japonicum* and *A. halleri* (Supplementary Figure S27). *N. benthamiana* is a model plant used for various studies on phytopathogen interactions (51). *P. japonicum* is a hemiparasitic plant that has been used for studies on the mechanism of parasitism to other plants (52). *L. japonicus* is a legume and has been used for studies on nodulation (53). *A. halleri* is a model of metal-accumulating plants and a closely related species of *A. thaliana* (24,54). *A. halleri* is used for studies of comparative genomics in addition to that of metal accumulation (55). Despite the importance of these species, their TSSs have been very poorly analyzed. For each species, TSS-seq2 libraries were constructed in duplicate (Supplementary Tables S1 and S7). For *P. japonicum*, we analyzed both roots with and without induction of prehaustoria formation by syringic acids (56). We found very high correlations (*R* = 0.99) in TSS expression levels between replicates, except for *A. halleri*, for which we analyzed the leaf samples from naturally growing plants (*R* = 0.88) (Supplementary Figure S28). Because *A. halleri* is a self-incompatible outcrossing species, two replicates taken from different individuals are expected to have different allelic combinations in many loci across the genome. To verify TSS consensus motifs in these plants, we created a sequence consensus for each plant species (Supplementary Figure S29). It is known that plant TSSs (−1/ +1) have a dinucleotide motif known as the ‘YR Rule (C/T A/G)’ (57). The motifs of the ‘YR Rule’ were observed in all plant species. We also performed RNA-seq analyses of the same samples to compare the precision of the expression levels of detected TSSs (Supplementary Tables S8, S9, and Supplementary Data 1). Reasonable correlations (*R* = 0.81–0.86) were obtained between RNA-seq and TSS-seq2 (Supplementary Figure S30).

**Figure 4.**
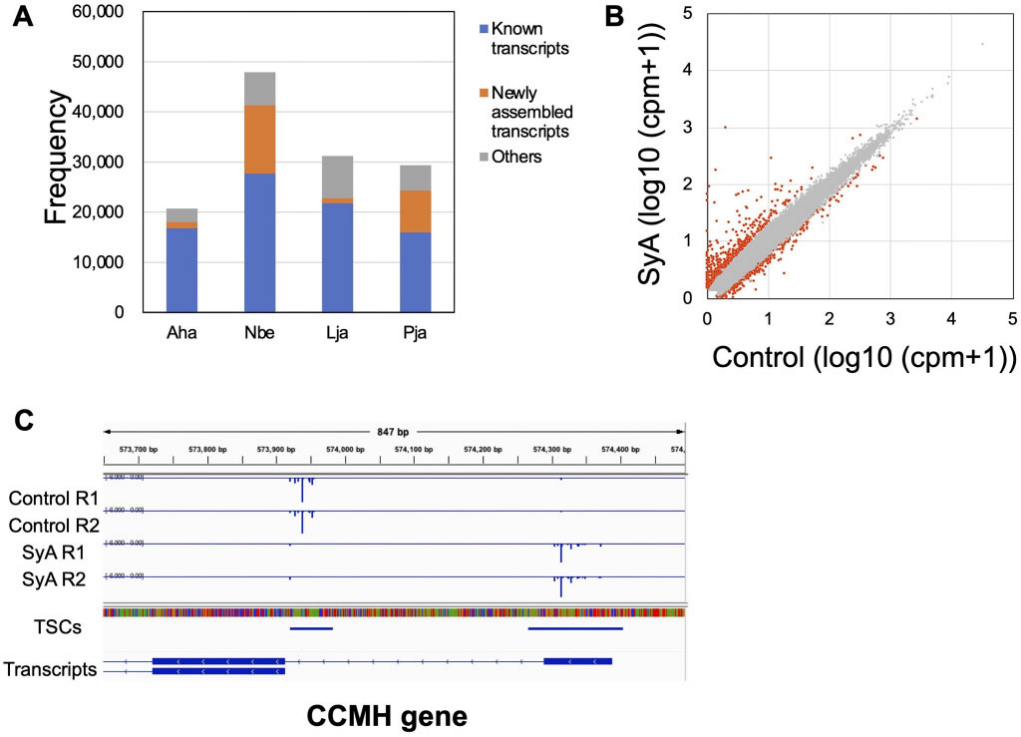
TSS catalog for four plant species. (**A**) The number of TSCs detected in both replicates of *A. halleri* (Aha), *N. benthamiana* (Nbe), *L. japonicus* (Lja), *P. japonicum* (Pja) (Supplementary Figure S31 and Supplementary Data 2). For *P. japonicum*, TSCs detected in the control or induction of prehaustoria formation by syringic acids were merged using the merge function of bedtools (35). The portion of the TSCs located within 500 bp of the TSS in the known transcripts and newly assembled transcripts from RNA-seq data (Supplementary Data 1) are shown by the blue and orange bars, respectively. (**B**) Scatterplot of expression levels (log10 (cpm + 1)) of TSCs for the control and induction of prehaustoria formation in *P. japonicum*. Differentially expressed TSCs as detected by edgeR (36) and other genes are shown as orange and gray dots, respectively. (**C**) Examples of genes with TSS switching. Blue bars show cpm expression values of TSS per position, and the cpm of the minus strand was shown as a negative value. TSCs are shown as blue boxes. The merged assembled transcripts are shown, respectively.

The obtained TSS data were used to construct a TSC catalog for each species. To select high-confidence TSCs, we selected the TSCs (20665–47871) that were commonly detected between replicates (Supplementary Figure S31 and Supplementary Data 2). Note that 83%–94% of the TSCs detected in each experiment were also detected in replicates. The one exception was the field samples of *A. halleri* (75–77%). To evaluate the detected TSCs, we compared TSCs with TSSs present in existing gene annotation datasets, including the assembled transcripts for each species (Supplementary Table S9 and Supplementary Data 1). 53%–81% of the identified TSCs were located within ± 500 bp from the TSSs of the pre-existing annotated transcripts (Figure 4A). Furthermore, when the TSSs of transcripts assembled from the RNA-seq data were also considered, we found that 73–87% of TSCs were located within ± 500 bp from the TSSs (Figure 4A and Supplementary Figure S32A). TSCs more than 500 bp away from the annotated TSSs of the transcripts are shown in Supplementary Figure S32B. These TSSs may provide important information to enrich the current transcript annotation of the respective genomes. Most methods for TSS detection are affected by RNA quality and/or contaminants, such as DNA and polysaccharide. For the samples from four plants, genomic DNA removal by DNase treatment was not performed and RNA quality was not always high. Especially, the leaf samples from naturally growing plants of *A. halleri* showed lower RNA quality than others (Supplementary Figure S27). Although we did not test it ourselves, other alternative methods are relatively vulnerable by these adverse features. For TSS-seq2, even starting from samples from potentially problematic materials, the TSCs called from *A. halleri* showed a high rate of overlap with previously annotated TSS (Figure 4A). We believe that this high-quality data was obtained owing to the high selectivity of 5′ cap of improved TSS-seq2, thus enabling TSS analysis of samples difficult to be analyzed by other methods.

For *P. japonicus*, we evaluated the usage change in TSCs by inducing prehaustoria formation. We found that 1131 TSCs showed differential expression between the control and prehaustoria samples (Figure 4B and Supplementary Table S10). The TSCs that showed gene expression changes included known key prehaustoria-related genes, including quinone reductase 2 QR2 (58), indole-3-pyruvate monooxygenase YUCCA3 (59), auxin efflux carrier component PIN3 (60), and 1-aminocyclopropane-1-carboxylate oxidase 5 ACO (23) (Supplementary Figure S33). In addition to changes in the expression level of a single TSC, changes in TSS usage between two independent possible alternative promoters was also observed for the cytochrome C-type biogenesis protein CCMH gene. Thus, TSS-seq2 analysis can also be used for understanding promoter switching, which has been shown to play an important role in plants (61).

In this study, we demonstrated the application of this developed improved method for the precise detection of hitherto-unannotated plants, for which such an analysis would be difficult by other alternative methods. However, we did not perform further in-depth analysis regarding how the newly detected TSCs and their expression levels may provide a novel insight for these plant species, because we did not want to overextend our report. More detailed analyses of the plants data will be published elsewhere. At least, we reported that, from a similar analysis for the model plant Arabidopsis, alternative promoter selection is the most important, novel mechanism for phytochrome-mediated gene expression (61). Therefore, we believe that TSS-seq2 could increase the potential for novel discoveries in other plants with more difficult features. In addition, the lower requirement of input amount with even low integrity and/or presence of contaminants should be advantageous for other purposes. For example, TSS-seq2 would enable TSS analysis of a very small tissue section obtained by laser-microdissection and rare cell types from which a sufficiently large amount of high-quality RNA cannot be obtained.

## Conclusions

In this paper, we describe the development of an improved method for TSS detection, TSS-seq2. A detailed evaluation showed that TSS-seq2 outperforms its originating method, TSS-seq, by overcoming drawbacks found in the original method. Moreover, TSS-seq2 shows an equivalent or even superior performance when compared to other representative methods, such as CAGE and ReCappable-seq. Compared to these methods, TSS-seq2 reactions proceed under relatively mild conditions and can therefore be easily conducted even by nonexpert researchers. In addition, we demonstrate that TSS-seq2 analysis of several nonmodel plants, including environmentally isolated wild plants, yielded unique results. Taking advantage of its robust performance, further modification of the TSS-seq2 method should also be envisioned. Further in detail characterization of TSSs in various species may reveal how their unique genomes initiate transcription of their genetic code.

## Supplementary Material

gkad1116_Supplemental_Files

## Data Availability

All raw sequencing data obtained in this study was deposited to DDBJ Sequence Read Archive (DRA) under the accession numbers DRA016093 and DRA017194.
