# Supplementary material for: An improved method for the highly specific detection of transcription start sites
Source: Nucleic Acids Res. 2023 Nov 22;52(2):e7. doi: 10.1093/nar/gkad1116 (PMC10810191; doi:10.1093/nar/gkad1116)
Supplement: gkad1116_Supplemental_Files [file gkad1116_supplemental_files.zip › Supplementary Materials add ref.pdf]

Supplementary Materials

Supplementary Table 1. Plant samples used in this study

Supplementary Table 2. Oligonucleotide sequences used in this study

Supplementary Table 3. A549 sequencing summary for TSS-seq1 and TSS-seq2

Supplementary Table 4. Summary statistics for randomly sampled reads

Supplementary Table 5. Performance evaluation for each method

Supplementary Table 6. Estimated cost for library preparation of TSS-seq2

Supplementary Table 7. Sequencing statistics for TSS−seq2 of plant samples

Supplementary Table 8. Sequencing statistics for RNA-seq of plant samples

Supplementary Table 9. Assembled transcript statistics for plant RNA-seq data

Supplementary Table 10. List of TSCs reveals differential expression between the control and induction of prehaustoria

Supplementary Figure 1. TSS-seq1 libraries
Electropherograms of the libraries of TSS-seq1 prepared from 5 μg, 500, and 50 ng of total RNA extracted from A549, respectively. The libraries were amplified with 13, 16, and 19 cycles of PCR, respectively. The concentrations of the libraries are shown under each electropherogram. The libraries were quantified with a DNA 7500 kit and a 2100 Bioanalyzer (Agilent Technologies).

Supplementary Figure 2. TSS-seq2 libraries
Electropherograms of the libraries of TSS-seq2 prepared from 5, 1 μg, 500, 50, and 5 ng of total RNA extracted from A549, respectively. The libraries were amplified with 12, 15, 16, 19, and 22 cycles of PCR, respectively. The concentrations of the libraries are shown under each electropherogram. The libraries were quantified with a DNA 7500 kit and a 2100 Bioanalyzer (Agilent Technologies).

Supplementary Figure 3. Qualities and yields of sequencing before and after the upper size removal

(**A**) Electropherograms of the libraries of TSS-seq2 after the upper size removal. The upper size removal was conducted for the TSS-seq2 libraries from 500 ng (replicate 1 and 2; **Supplementary Figure 2**) as follows: 2.5 µl of each library was brought to 20 µl by nuclease-free water, and 0.5× volume (10 µl) of AMPureXP beads (Beckman Coulter) was added to the diluted library. The supernatant containing shorter DNA fragments was recovered, and the DNA fragments were purified by 26 µl of AMPureXP beads. The libraries after the upper size removal were quantified with a DNA 7500 kit and a 2100 Bioanalyzer (Agilent Technologies). (**B** and **C**) The mean values of the base quality score (**B**) and the number of raw reads (**C**) obtained by sequencing by NovaSeq6000 (Illumina) are shown. Error bars represent standard errors between replicates.

Supplementary Figure 4. Breakdown of reads of libraries prepared by each method

The breakdown of reads of each library in A549. As for 20,000,000 raw reads randomly sampled for each dataset, the proportion of rRNA reads that aligned to rRNA sequences by Bowtie2 (20), UMI not detected reads as determined by UMItools (21), unmapped reads, PCR duplicates among reads mapped to the reference genome hg38 by STAR (29), and valid reads that passed all filtering steps are shown as a band graph.

Supplementary Figure 5. Optical duplicate rates of TSS-seq2 and the PCR-free WGS kit

(**A** and **B**) Optical duplicate rates of TSS-seq2 datasets of *P. japonicum* (**A**) and duplicate rates of PCR-free whole-genome sequencing (WGS) (**B**) are shown. As for TSS-seq2, because TSS-seq2 datasets of *P. japonicum* (control, replicate 1, and replicate 2) were used for the estimation of optical duplicate rates using MarkDuplicate of Picard v2.18.25 with the parameter "OPTICAL_DUPLICATE_PIXEL_DISTANCE=2500" for patterned flowcells employed by NovaSeq6000, the number of optical duplicate reads was estimated among mapped reads to a reference genome. The duplicate rates among total reads in 16 replicates of PCR-free WGS libraries prepared from Coriell sample NA12878 are also shown. The alignment stats of the datasets, known as "NovaSeq S4: Illumina DNA PCR-free (v1.5 chemistry)," were obtained from the website of BaseSpace Sequence Hub of Illumina (https://basespace.illumina.com/dashboard). The libraries were prepared by Illumina DNA PCR-Free Prep Kit, sequenced by NovaSeq6000 sequencer, and processed by the DRAGEN Germline BaseSpace App.

Supplementary Figure 6. Heatmap of expression levels of TSS measured by TSS-seq 1 and 2 around annotated TSSs

A line graph of the average cpm value and a heatmap of cpm for TSS-seq1 and 2 (replicate 1) are shown. Gencode TSSs with expression levels greater than 1 tpm (Transcript Per Million) estimated from the RNA-seq dataset using StringTie v2.1.5 (31) that overlapped with ATAC peaks in a dataset downloaded from the ENCODE website (https://www.encodeproject.org/files/ENCFF876UEM/) (34) were used as reference TSSs. Line graphs and heatmaps were generated using deeptools v3.4.2 (63).

Supplementary Figure 7. Expression levels of TSCs for each category of overlap patterns between TSS-seq1 and 2

Boxplots of the expression pattern of TSCs for each group classified from the overlap patterns in **Figure 2D**. Cpm value estimated by TSSr(30) was used as expression value. Box plots were prepared using the boxplot function of R.

Supplementary Figure 8. Correlation of TSS counts among TSS-seq1 datasets

Scatterplots of counts of TSS reads for TSS-seq1 datasets in A549. Pearson correlations are shown in the graphs. These plots were prepared using the plotCorrelation function of TSSr(30) (see the legend of **Figure 2F** for details).

Supplementary Figure 9. Comparison of expression levels estimated by RNA-seq and TSS-seq2

Scatterplots of gene expression levels between RNA-seq and TSS-seq2 in A549. RNA-seq data of A549 obtained in our previous study(27) was used. To estimate gene expression levels, reads of TSS-seq2 and RNA-seq mapped to each gene were counted using featureCounts version 1.6.4 with Gencode v40. Tpm (transcript per million) and cpm values were calculated from the counts of RNA-seq and TSS-seq reads, respectively. Pearson correlation coefficients between log10 (tpm+1) of RNA-seq and log10 (cpm+1) of TSS-seq2 are shown in the plots.

Supplementary Figure 10. TSS-seq2 from fragmented RNA

(**A** and **B**) Bioanalyzer traces of fragmented RNA samples (**A**) and libraries of TSS-seq2 prepared from them (**B**). The total RNA of A549 was fragmented using the NEBNext Magnesium RNA Fragmentation Module (New England Biolabs) with two treatment durations (1 min and 2 min). Using Bioanalyzer 2100 and the RNA Nano 6000 Kit

(Agilent Technologies), were quantified and their DV200 values of the fragmented RNA samples (which are percentages of RNA fragments that are longer than 200 nt) were estimated. TSS-seq2 libraries were prepared from 500 ng of fragmented RNA with 15 cycles of PCR. The prepared libraries were quantified using Bioanalyzer 2100 and the DNA7500 Kit. Note: DV200 is the percentage of RNA fragments longer than 200 nt. This value is used as an indicator of highly fragmented RNA, beyond the evaluation limit by the RIN value. (**C** and **D**) Comparison of TSS-seq2 libraries prepared from high-quality RNA (identical with TSS-seq2 from 500ng, Replicate 1) and fragmented RNA. To remove the bias of sequencing depth, 20,000,000 raw reads were randomly sampled for each dataset and were processed in the same manner as the other TSS-seq2 libraries of A549. (**C**) Breakdown of 5′ ends of mapped positions of TSS reads overlapping with the promoter (i.e., ±100 bp of TSSs), exon, intron, and intergenic regions of Gencode transcripts. (**D**) Scatterplots of counts of TSS reads. Pearson's correlations are shown on the graphs. These plots were prepared using the plotCorrelation function of TSSr(30) (see the legend of **Figure 2F** for details).

Supplementary Figure 11. Comparison of libraries prepared using splint adapters with/without UMI sequences

(**A**) Scatterplots of counts of TSS reads for the datasets of the libraries with and without UMI sequences prepared from 500 ng of total RNA of A549. Pearson's correlations are shown in the graphs. Following the standard procedure of TSSr (30), we extracted the count of the 1st bp position of TSS reads from BAM files using the getTSS function of TSSr, and the counts were used as input data for the plotCorrelation function of TSSr to prepare the plot (see also the legend of **Figure 2F** for details). (**B**) Breakdown of 5′ ends of mapped positions of TSS reads overlapping with the promoter (i.e., ±100 bp of TSSs), exon, intron, and intergenic regions of Gencode transcripts. Each fraction was estimated using the intersect function of bedtools (36). From 500 ng of total RNA of A549, the TSS-seq2 library without UMI was prepared following the standard protocol of TSS-seq2, except for the replacement of the splint adapter with that without UMI (**Supplementary Table 2**).

To validate the effects of the UMI sequence on the ligation reaction, we prepared a TSS-seq2 library using splint adapters without UMIs from 500 ng of A549 total RNA. For the comparison, we employed the data of the TSS-seq2 with UMIs which was prepared from the same amount of RNA (500 ng input, replicate 1), and used all raw reads of both libraries for the following analyses. As the library prepared without UMIs cannot be processed for the deduplication, this step was omitted also for the data with UMIs for

equal comparison. In this analysis, we observed a strong correlation between the datasets with and without UMIs (R=0.87) (**A**). To evaluate differences in specificity for TSS detection in more detail, we assessed overlapping with Gencode transcripts in read levels (**B**). In both datasets, mapped positions of 5′ ends of TSS reads showed a high overlapping rate (94% and 97% without and with UMIs, respectively) with the core promoter region (±100 bp around the TSSs of Gencode transcripts). Therefore, we considered that the inclusion of UMIs should have a limited effect (if any) on the representation of the resulting library. We consider this to be partly because, in the TSS-seq2, a high concentration of the splint adapter is included for the splint-ligation reaction; thus, the hybridization of RNA molecules may have been saturated. Also note that, as there is a fixed sequence, "GAGA," at the 3′ end of the adapter, this sequence is intended to play a role as a scaffold for splint ligation reactions.

Supplementary Figure 12. Comparison of GC-rich and AT-rich TSCs

Scatter plot of gene expression measured by RNA-seq and that of the major TSC of the gene measured by TSS-seq2. TSCs of 5 μg input of replicate 1, called by TSSr with a threshold of ≥ 1 read per TSC, were used for this analysis. The cpm value of each TSC (as the expression level) was calculated by TSSr (30). Using the closest function of bedtools (36), the closest Gencode gene on the same strand, whose annotated TSS is located within 1 kb, was associated with each TSC. For each gene, the TSC with the highest expression among TSCs associated with the gene was defined as the major TSC of the gene. The GC% of 6 bp downstream from the summit of the major TSC, which is the most frequently used TSS position within the TSC, was estimated as the GC% of the TSC. Genes were classified into two groups, with CG-rich (GC% ≥ 60%) and AT-rich (GC% ≤ 40%) TSCs. RNA-seq reads mapped to each gene were counted using featureCounts version 1.6.4 with Gencode v40. Tpm (transcript per million) values were calculated from the counts of RNA-seq reads. Pearson's correlation coefficients between log10 (tpm+1) of RNA-seq and log10 (cpm+1) of TSS-seq2 are shown in the plots.

Using a mixture of synthetic miRNA, Maguire et al. have demonstrated a lower bias of randomized splint ligation on the RNA ligation compared with single-strand ligation-based methods (18). We have also tried to evaluate whether there is any representation bias in the 5' base contents caused by the NNNNNN sequence of the splint adapter. We classified the TSCs of the genes into two groups, which are TSCs of GC% ≥60% and TSCs of GC% ≤40% (**Supplementary Figure 12**; the GC% at 6 bp downstream from the peak of TSC, which is the most frequently used TSS position within the TSC, were used for calculating the GC% of the TSC). For each group of the TSCs,

the TSS-seq2 data were compared with the RNA-seq data. Similar Pearson's correlation coefficients (R= 0.68 and 0.70) were observed with a similar slope of the regression line (0.61 and 0.65) for the GC-rich and AT-rich TSCs, respectively. Also, we could not observe any obvious bias in the base contents surrounding the detected TSS, compared with CAGE (**Figure 3D**). Therefore, the effect of the NNNNNN sequence in the splint oligo, at least at this length and under this reaction condition, should not cause a significant bias (if any) on the representation of the mRNA at least in a practical manner.

Supplementary Figure 13. Reduction in misdetections of exonic TSCs by TSS-seq2
Typical view of the distribution of 5′ ends of TSS reads and TSCs for TSS-seq1 from 5 μg, 500, and 50 ng, and TSS-seq2 of replicates 1 from 5, 1 μg, 500, 50, and 5 ng. First exons and other exons of the EGFR and RPL11 genes are shown. Blue bars indicate expression values (cpm; Count Per Million) of TSS per position. TSS clusters (TSCs) detected by TSSr with ≥1 cpm and ≥2 reads are shown as black boxes.

Supplementary Figure 14. PCR duplicate rate in TSS-seq2 prepared from various input amounts
For TSS-seq2 dataset for A549, the average PCR duplicate rates among mapped reads of replicates are shown. Error bars represent standard error between replicates.

Supplementary Figure 15. Comparison of TSS-seq2 datasets with 5 ng and 5 μg input
(**A**) Overlap of TSCs detected by replicates 1 (R1) of TSS-seq2 prepared from 5 μg (left pink circles) and 5 ng (right dark cyan circles) total RNA of A549. Number of TSCs uniquely detected by 5 μg or 5 ng input dataset, and those of 5 μg input overlapping with those of 5 ng, respectively. Number of TSCs of 5 ng input overlapping with those of 5 μg input shown in parentheses. Each group of TSCs was extracted using the intersect function of bedtools(36). The total numbers of TSCs for each dataset are also shown above each Venn diagram. Overlap was defined as overlapping by ≥1 bp. Most TSCs span tens to hundreds of base pairs. Therefore, when the TSCs were compared between different datasets, a TSC of a given dataset occasionally includes several TSCs of other datasets. The numbers of the "common" TSCs between 5 μg and 5 ng input differ from each other. (**B**) Boxplots of expression pattern of TSCs for each group classified from the overlap patterns in **A**. Cpm value estimated by TSSr(30) used as expression value. Box plots were prepared using the boxplot function of R.

Supplementary Figure 16. Typical views of TSS-seq2 libraries from small inputs before and after additional size selection

Electropherograms of the libraries of TSS-seq2 prepared from 5 ng of total RNA extracted from A549 (identical with TSS-seq2 library from 5 ng, replicate 1) before and after additional size selection, respectively. For size selection, 8 μl of SPRI select (Beckman Coulter) was added to 10 μl of the library. After washing with 80% Ethanol twice, the library was eluted in 11 μl of nuclease-free water. Additional size selection was conducted also for TSS-seq library from 50 ng, replicate 1. For the 50 ng and 5 ng libraries, size-selection was repeated twice and thrice, respectively. The libraries were quantified with a High-Sensitivity DNA Kit and a 2100 Bioanalyzer (Agilent Technologies).

Supplementary Figure 17. Saturation curve of sequencing depth in 50 and 5 ng inputs after size selection

For 50 ng and 5 ng input libraries before and after size selection, 1, 2.5, 5, 10, 20, 30, and 40 M raw reads were randomly sampled twice. As for the 50 ng input before size selection, sampling of 40 M could not be conducted because the total number of those is <40 M. For each dataset, the number of valid reads was estimated. The average of the number of valid reads is shown in the plot (left). The numbers when using all raw reads were also included. An enlarged view of the squared area of the plot is also shown (right).

Supplementary Figure 18. Expression levels of TSCs that overlap and do not overlap with promoters

As for TSS-seq2 (5μg), CAGE, and Recappable-seq in A549, boxplots of expression levels by overlapping patterns with promoters are shown. TSCs with expression levels of ≥1 cpm used in **Figure 3C** were grouped into promoter and non-promoter (exon, intron, intergenic) TSCs. Using the expression levels of each TSC estimated by TSSr, boxplots were prepared using the boxplot function of R.

Supplementary Figure 19. RACE analysis of TSCs uniquely detected by TSS-seq2

5′ RACE regarding the TSCs of two genes (*THSD7A* and *UNC13D*) that are detected by only TSS-seq2, compared to CAGE and ReCappable-seq. TSS counts of RACE and cpm values and TSCs of replicates 1 for TSS-seq2 (5 μg), CAGE, and Recappable-seq are shown. Blue bars indicate TSS counts for RACE and expression values (cpm) of TSS per position for the other methods, and those of the minus strand are shown as a negative value. TSCs detected by TSSr with ≥1 cpm are shown as black boxes. Gencode transcript models are also shown at the bottom. The 5′ RACE libraries were prepared by amplifying

the 5′ end of each transcript from the TSS-seq1 library (5 µg input) using gene-specific primers and the primer hybridizing capping oligo sequence (**Supplementary Table 2;** (see also the **Materials and Methods** section for details).

Supplementary Figure 20. Sequence logos for ReCappable-seq and TSS-seq
Sequence consensus within ±10 bp from mapped positions of 5′ ends of TSS reads for ReCappable-seq of replicate 1 (28) and the original TSS-seq data (27). Sequence logos were generated using weblogo version 3.7.9 (62).

Supplementary Figure 21. Comparison of TSS-seq2 and Recappable-seq at a 1 bp resolution
(**A and B**) Breakdown of TSSs at a 1 bp resolution commonly detected by TSS-seq2 (TSS2; 5 µg, replicate 1) and Recappable-seq (Recap; 5 µg, replicate 1) and those uniquely detected by each method that were not detected by the other method with even 1 read. TSSs with ≥1 cpm were used for these analyses. Fractions of TSSs overlapping with promoter (±100 bp from Genocode TSS) (**A**) and regions from 5'-mapped positions of at least one CAGE read to 4 bp downstream (Replicate 1) (**B**) were shown. (**C**) The distribution of TSS reads of TSS2, Recap, and CAGE of replicates 1 around annotated the TSS of KRAS and MYC genes. Blue bars indicate expression values (cpm) of TSS per position, and cpm of the minus strand are shown as a negative value. (**D**) Sequence consensus within ±10 bp from TSSs commonly detected by TSS2 (5 µg, replicate 1) and Recap (5 µg, replicate 1) and those uniquely detected by each method. Sequence logos were generated by Weblogo version 3.7.9 (62).

Supplementary Figure 22. Evaluation of precision with various criteria
The precision of TSS (position) detection in single base resolution was evaluated by overlapping with open chromatin, promoter region, and CAGE reads. TSSs whose expression levels were ≥1 cpm in each method were extracted. The TSSs were classified as true positives (TPs) and false positives (FPs) by overlapping with open chromatin regions detected by ATAC-seq in a dataset downloaded from the ENCODE website (https://www.encodeproject.org/files/ENCFF876UEM/) (34), core promoter regions (±100 bp from Gencode TSS), or regions from 5′-mapped positions of at least one CAGE read to 4 bp downstream (Replicate 1), and estimated precision scores. Because the shift of the TSS position was caused by the addition of extra bases due to the terminal deoxynucleotidyl transferase activity of the RT enzyme in CAGE, up to four base pairs downstream of 5′-mapped positions of CAGE reads were considered when being used as

the reference for precision estimation. The bar plot shows the average of precision between replicates, except for the original TSS-seq without replicates. The error bars indicate the standard error between replicates of each dataset.

Supplementary Figure 23. Comparison between TSS-seq2 and CAGE replicates
Overlap of TSCs between TSS-seq2 (**A**) and CAGE(28) replicates (**B**). TSCs detected by replicates 1 (R1; left pink circles) and 2 (R2; right dark cyan circles). Numbers of TSCs uniquely detected in replicates 1 (R1) or 2 (R2), and those of R1 that overlapped with those of R2, respectively as well as the numbers of TSCs of R2 overlapping with those of R1. Those detected in both replicates were extracted and merged using the intersect and merge function of bedtools (36). The number of merged TSCs is also shown. TSCs were detected by TSSr(30) with the parameter showing the highest F1 score (**Supplementary Table 5**). The total numbers of TSCs for each dataset are also shown above each Venn diagram. Overlap was defined as overlapping by ≥1 bp. Most TSCs span tens to hundreds of base pairs. Therefore, when the TSCs were compared between different datasets, a TSC of a given dataset occasionally includes several TSCs of other datasets. The numbers of "common" TSCs among merged TSCs, R1, and R2 differ from one another.

Supplementary Figure 24. Comparison of TSCs between TSS-seq2 and CAGE filtered at a threshold of ≥1 cpm and ≥2 read per TSC
(**A**) Overlap of TSCs detected by each dataset of TSS-seq2 (left pink circles) and replicate 1 of CAGE (right dark cyan circles) with a threshold of ≥1 cpm and ≥2 read per TSC in A549. Numbers of TSCs uniquely detected by TSS-seq1 or CAGE, and those of TSS-seq2 input overlapping with those of CAGE, respectively. The numbers of TSCs of CAGE overlapping with those of TSS-seq2 are also shown in parentheses. Each group of TSCs was extracted using the intersect function of bedtools (36). The total numbers of TSCs for each dataset are also shown above each Venn diagram. Overlap was defined as overlapping by ≥1 bp. Most TSCs span tens to hundreds of base pairs. Therefore, when the TSCs were compared between different datasets, a TSC of a given dataset occasionally includes several TSCs of other datasets. The numbers of "common" TSCs between TSS-seq2 and CAGE differ from each other. (**B**) Boxplots of the expression pattern of TSCs for each group classified from the overlap patterns in **A**. Cpm value estimated by TSSr (30) used as expression value. Box plots were prepared using the boxplot function of R. The unique parts mostly correspond to genes with low expression levels in the same manner as overlap patterns between TSS-seq1 and 2 (**Supplementary Figure 7**).

Supplementary Figure 25. Expression levels of TSCs for each category of overlap patterns between TSS-seq2 and CAGE

Boxplots of the expression pattern of TSCs for each group classified from the overlap patterns in **Figure 3H**. After counting the TSS counts of each replicate for each TSC category using the intersect function of bedtools(36), the average cpm values of replicates were used as expression value. Box plots were prepared using the boxplot function of R.

Supplementary Figure 26. Workflow of TSS-seq2 library preparation

The step for split adapter preparation shown in the green box can be performed within the incubation time for dephosphorylation or mRNA decapping. The orange box indicates the optional procedure for improving the yield of valid reads, particularly for the low-input cases.

Supplementary Figure 27. Quality of plant total RNA

Electropherograms of total RNA for four plant species (*N. benthamiana*, *L. japonicus*, *A. halleri*, and *P. japonicum* [control and treatment of syringic acid]). RNA samples were quantified with an RNA 6000 Nano kit and a 2100 Bioanalyzer (Agilent Technologies). The RNA integrity Number (RIN) of each sample are shown in plots.

Supplementary Figure 28. Correlation of TSS counts between plant sample replicates

Scatterplots of TSS counts produced by TSS-seq2 for each plant species (*N. benthamiana*, *L. japonicus*, *A. halleri*, and control (Pja) and sample with treatment of syringic acid (Pja_SyA) of *P. japonicum*). For *P. japonicum*, all combinations among datasets of controls and treatment of syringic acid were compared. Pearson correlations are shown in the graphs. Plots were prepared using the plotCorrelation function of TSSr (30) (see the legend of **Figure 2F** for details).

Supplementary Figure 29. TSS consensus sequences for plant samples

Sequence consensus within ±10 bp from mapped positions of 5′ ends of reads of TSS-seq2 for four plant species (*N. benthamiana*, *L. japonicus*, *A. halleri*, and *P. japonicum*). Sequence logos were generated using weblogo version 3.7.9 (62).

Supplementary Figure 30. Expression estimates for RNA-seq and TSS-seq2 analysis of plant samples

Scatterplots of the gene expression levels between RNA-seq and TSS-seq2 for four plant species (*N. benthamiana*, *L. japonicus*, *A. halleri*, and *P. japonicum* (control and treatment of syringic acid)). To estimate gene expression levels, the reads of TSS-seq2 and RNA-seq mapped to each gene were counted using featureCounts version 1.6.4 using a merged transcript model (**Supplementary Data 1**). Tpm and cpm values were calculated from the counts of the RNA-seq and TSS-seq reads, respectively. Pearson correlation coefficients between log10 (tpm+1)) of RNA-seq and log10 (cpm+1)) of TSS-seq2 are shown in the plots.

Supplementary Figure 31. Overlapping TSCs between replicates in plants

Venn diagram of TSC overlap detected in replicates 1 (pink circles) and 2 (red circles) of *N. benthamiana*, *L. japonicus*, *A. halleri*, and *P. japonicum* (control and treatment of syringic acid). Number of TSCs uniquely detected in one replicate and commonly detected in both replicates. Using the intersect and the merge function of bedtools version 2.29.0(36) , each group of TSCs was extracted and common TSCs merged. The numbers of common TSCs after merging are shown. Overlap was defined as overlapping by ≥1 bp.

Supplementary Figure 32. Unannotated TSCs detected by TSS-seq2 in plants

(**A** and **B**) Typical views of unannotated TSCs newly detected by TSS-seq in plant samples (*N. benthamiana*, *L. japonicus*, *A. halleri*, and *P. japonicum*). TSCs overlapping with the TSS of assembled transcripts (**A**) and those more than 500 bp away from the TSSs of pre-existing annotations and assembled transcripts (**B**) are shown in the black boxes. Blue bar plots show cpm expression values of TSS per position, and the cpm of the minus strand was shown as a negative value. TSCs are shown as blue boxes. Pre-existing transcript annotations, the transcripts merged with the annotation, and transcripts assembled from RNA-seq data are shown, respectively.

Supplementary Figure 33. TSCs showed expression changes in response to induction of prehaustoria in *P. japonicum*

Blue bars show cpm expression values of TSS per position, and cpm values of the minus strand are shown as negative values. TSCs are shown as blue boxes. The respective merged assembled transcripts are shown. Expression levels of each TSCs for each sample are shown as cpm values in tracks.

Supplementary Data 1. GTF files of assembled transcripts from plant RNA-seq datasets.

Supplementary Data 2. BED files of TSCs in plants.

Supplementary Methods

Sample preparation of *Nicotiana benthamiana*

Here we used an *N. benthamiana* lab strain (22). *Nicotiana benthamiana* seeds were first surface-sterilized in 5% bleach for 5 min, washed three times with sterile water, incubated at 4°C for 3 d, then planted on a medium containing half-strength Murashige and Skoog medium, 0.5% sucrose, and 1% agar. The pH was adjusted to 5.8 with 1 M KOH. Seedlings were grown at 27°C under 100 μmol m^{-2} s^{-1} of continuous illumination conditions optimized for *N. benthamiana*. Fourteen days after germination, whole plant tissues were collected from three plants and RNA was extracted. Whole *N. benthamiana* plants were snap-frozen in liquid nitrogen then ground using a pestle. RNA was extracted using an RNeasy Plant Mini Kit (Qiagen) as per the manufacturer's instructions.

Sample preparation of *Lotus japonicus*

The Miyakojima MG-20 ecotype of *Lotus japonicus* was used for this study(64). Plants were grown with *Mesorhizobium loti* MAFF 303099 in autoclaved vermiculite supplemented with Broughton and Dilworth solution (65) without a nitrogen source. Plants were grown under a 16 h light/8 h dark cycle at 24°C in a growth cabinet.

Total RNA was isolated from whole roots of plants three days after rhizobial inoculation using the PureLink Plant RNA Reagent (Thermo Fisher Scientific). All procedures were performed as per the manufacturer's instructions.

Sample preparation of *Arabidopsis halleri*

Leaves of environmentally isolated two individuals of *A. halleri* subsp. *gemmifera* were collected on February 1, 2022 in a natural population(66). Harvested leaves were preserved in RNA*later* Stabilization Solution (Thermo Fisher Scientific), kept on ice during the transport from the field to the laboratory, kept at 4 °C overnight, and stored at −20 °C. After bead homogenization using a Multi-beads Shocker (Yasui Kikai), RNA was extracted from the leaves using TRIzol Reagent (Thermo Fisher Scientific) with all procedures following the manufacturer's instructions.

Sample preparation of *Phtheirospermum japonicum*

P. japonicum (Thunb.) Kanitz (ecotype Okayama) seeds were sterilized with 10% (v/v) commercial bleach solution (Kao) for 5 min after which they were washed at least five times with sterilized water. The seeds were then sown on one-half-strength Murashige

and Skoog medium (Wako Chemicals) with 1% (w/v) sucrose and 0.65% (w/v) agar. They were first stratified at 4°C in the dark for two days. Seeds germination was conducted in dark conditions for three days at 25°C after which plants were grown in long-day conditions (i.e., 16 h light with 100 µmol m^{-2} s^{-1}, 8 h dark) at 25°C. One-week-old *P. japonicum* seedlings were then transferred to a nutrient-free 0.8% (w/v) agar medium for a three-day starvation treatment. Subsequently, seedlings were transferred to 0.8% (w/v) agar medium supplemented with or without 10 µM syringic acid and grown vertically for 24 h. *P. japonicum* root tips (i.e., regions approximately 1-2 mm from the root tip) were harvested and total RNA was then extracted using an RNeasy Plant Mini Kit. All procedures were performed according to the manufacturer's instructions. Two biological replicates were prepared for this experiment.

Supplementary References

63. Ramírez,F., Ryan,D.P., Grüning,B., Bhardwaj,V., Kilpert,F., Richter,A.S., Heyne,S., Dündar,F. and Manke,T. (2016) deepTools2: a next generation web server for deep-sequencing data analysis. *Nucleic Acids Res.*, **44**, W160–W165.

64. Kawaguchi,M. (2000) Lotus japonicus ‘Miyakojima’ MG-20: An early-flowering accession suitable for indoor handling. *J. Plant Res.*, **113**, 507–509.

65. Broughton,W.J. and Dilworth,M.J. (1971) Control of leghaemoglobin synthesis in snake beans. *Biochem. J.*, **125**, 1075–1080.

66. Kudoh,H., Honjo,M.N., Nishio,H. and Sugisaka,J. (2018) The Long-Term ‘In Natura’ Study Sites of Arabidopsis halleri for Plant Transcription and Epigenetic Modification Analyses in Natural Environments. *Methods Mol. Biol.*, **1830**, 41–57.

Supplementary Table 1. Plant samples used in this study

Sample Name	Species	Parts	Treatment	Replicate
Nbe_R1	*Nicotiana benthamiana*	Whole	No	1
Nbe_R2				2
Aha_R1	*Arabidopsis halleri* subsp. *gemmifera* (two individuals growing in a natural population)	Leaves	No	1
Aha_R2				2
Lja_R1	*Lotus japonicus* (strain MG20)	Root	Rhizobial infection	1
Lja_R2				2
Pja_ctrl_R1	*Phtheirospermum japonicum* (ecotype Okayama)	Root	No	1
Pja_ctrl_R2				2
Pja_SyA_R1			Syringic acid	1
Pja_SyA_R2				2

Supplementary Table 2. Oligonucleotide sequences used in this study

5' Splint adapter with UMI (RNA oligos)

Oligo name	Oligo sequence
5’ UMI oligo	rCrUrArCrArCrGrArCrGrCrUrCrUrUrCrCrGrArUrCrUrNrNrNrNrNrNrNrNrGrArGrA
5’ UMI splint	mNmNmNmNmNmNrUrCrUrCrNrNrNrNrNrNrNrNrArGrArUrCrGrGrArArGrArGrCrGrUrCrGrUrGrUrArG/3’InvT/

*r is RNA, m is 2-O'-Methyl RNA, and 3'InvT is 3' Inverted dT modification.

5' Splint adapter without UMI (RNA oligos)

Oligo name	Oligo sequence
5’ oligo without UMI	rCrUrArCrArCrGrArCrGrCrUrCrUrUrCrCrGrArUrCrU
5’ splint without UMI	mNmNmNmNmNmNArGrArUrCrGrGrArArGrArGrCrGrUrCrGrUrGrUrArG/3’InvT/

*r is RNA, m is 2-O'-Methyl RNA, and 3'InvT is 3' Inverted dT modification.

RT primer (DNA oligo)

Primer name	Primer sequence
TSS-seq2 RT	GTGACTGGAGTTCAGACGTGTGCTCTTCCGATCTNNNNN

PCR primers with unique dual indexes (DNA oligos)

Primer name (i7)	Primer sequence (i7)	Primer name (i5)	Primer sequence (i5)
i7_UDP1	CAAGCAGAAGACGGCATACGAGATCGCTCAGTTCGTGACTGGAGTTCAGACGTGTGCTCTTCCGATCT	i5_UDP1	AATGATACGGCGACCACCGAGATCTACACTCGTGGAGCGACACTCTTTCCCTACACGACGCTC
i7_UDP2	CAAGCAGAAGACGGCATACGAGATTATCTGACCTGTGACTGGAGTTCAGACGTGTGCTCTTCCGATCT	i5_UDP2	AATGATACGGCGACCACCGAGATCTACACCTACAAGATAACACTCTTTCCCTACACGACGCTC
i7_UDP3	CAAGCAGAAGACGGCATACGAGATATATGAGACGGTGACTGGAGTTCAGACGTGTGCTCTTCCGATCT	i5_UDP3	AATGATACGGCGACCACCGAGATCTACACTATAGTAGCTACACTCTTTCCCTACACGACGCTC
i7_UDP4	CAAGCAGAAGACGGCATACGAGATCTTATGGAATGTGACTGGAGTTCAGACGTGTGCTCTTCCGATCT	i5_UDP4	AATGATACGGCGACCACCGAGATCTACACTGCCTGGTGGACACTCTTTCCCTACACGACGCTC
i7_UDP5	CAAGCAGAAGACGGCATACGAGATTAATCTCGTCGTGACTGGAGTTCAGACGTGTGCTCTTCCGATCT	i5_UDP5	AATGATACGGCGACCACCGAGATCTACACACATTATCCTACACTCTTTCCCTACACGACGCTC
i7_UDP6	CAAGCAGAAGACGGCATACGAGATGCGCGATGTTGTGACTGGAGTTCAGACGTGTGCTCTTCCGATCT	i5_UDP6	AATGATACGGCGACCACCGAGATCTACACGTCCACTTGTACACTCTTTCCCTACACGACGCTC
i7_UDP7	CAAGCAGAAGACGGCATACGAGATAGAGCACTAGGTGACTGGAGTTCAGACGTGTGCTCTTCCGATCT	i5_UDP7	AATGATACGGCGACCACCGAGATCTACACTGGAACAGTAACACTCTTTCCCTACACGACGCTC
i7_UDP8	CAAGCAGAAGACGGCATACGAGATTGCCTTGATCGTGACTGGAGTTCAGACGTGTGCTCTTCCGATCT	i5_UDP8	AATGATACGGCGACCACCGAGATCTACACCCTTGTTAATACACTCTTTCCCTACACGACGCTC
i7_UDP9	CAAGCAGAAGACGGCATACGAGATCTACTCAGTCGTGACTGGAGTTCAGACGTGTGCTCTTCCGATCT	i5_UDP9	AATGATACGGCGACCACCGAGATCTACACGTTGATAGTGACACTCTTTCCCTACACGACGCTC
i7_UDP10	CAAGCAGAAGACGGCATACGAGATTCGTCTGACTGTGACTGGAGTTCAGACGTGTGCTCTTCCGATCT	i5_UDP10	AATGATACGGCGACCACCGAGATCTACACACCAGCGACAACACTCTTTCCCTACACGACGCTC
i7_UDP11	CAAGCAGAAGACGGCATACGAGATGAACATACGGGTGACTGGAGTTCAGACGTGTGCTCTTCCGATCT	i5_UDP11	AATGATACGGCGACCACCGAGATCTACACCATACACTGTACACTCTTTCCCTACACGACGCTC
i7_UDP12	CAAGCAGAAGACGGCATACGAGATCCTATGACTCGTGACTGGAGTTCAGACGTGTGCTCTTCCGATCT	i5_UDP12	AATGATACGGCGACCACCGAGATCTACACGTGTGGCGCTACACTCTTTCCCTACACGACGCTC

Gene-specific primers for 5′ RACE (DNA oligos)

Primer name	Primer sequence
RACE for UNC13D	GTGACTGGAGTTCAGACGTGTGCTCTTCCGATCTCTTATCTTGATGGCCTGGC
RACE for THSD7A	GTGACTGGAGTTCAGACGTGTGCTCTTCCGATCTGACTGGAAGGTCATTGGAAG

Supplementary Table 3. A549 sequencing summary for TSS-seq1 and TSS-seq2

Method	UMI	Amount of input RNA	Quality of RNA	Replicate	Additional size-selection	Read length	Number of reads
TSS-seq1	with UMI	5 µg	RIN: ~10		-	100SR	44,467,513
TSS-seq1	with UMI	500 ng	RIN: ~10		-	100SR	60,959,915
TSS-seq1	with UMI	50 ng	RIN: ~10		-	100SR	51,812,816
TSS-seq2	with UMI	5 µg	RIN: ~10	1	-	100SR	26,688,539
TSS-seq2	with UMI	5 µg	RIN: ~10	2	-	100SR	24,172,048
TSS-seq2	with UMI	1 µg	RIN: ~10	1	-	100SR	26,551,239
TSS-seq2	with UMI	1 µg	RIN: ~10	2	-	100SR	27,788,221
TSS-seq2	with UMI	500 ng	RIN: ~10	1	-	100SR	27,332,202
TSS-seq2	with UMI	500 ng	RIN: ~10	2	-	100SR	33,509,221
TSS-seq2	with UMI	50 ng	RIN: ~10	1	-	100SR	33,355,285
TSS-seq2	with UMI	50 ng	RIN: ~10	2	-	100SR	35,852,924
TSS-seq2	with UMI	5 ng	RIN: ~10	1	-	100SR	83,705,632
TSS-seq2	with UMI	5 ng	RIN: ~10	2	-	100SR	71,097,269
TSS-seq2	with UMI	50 ng	RIN: ~10	1	Lower size removal	100SR	51,980,436
TSS-seq2	with UMI	5 ng	RIN: ~10	1	Lower size removal	100SR	65,272,849
TSS-seq2	with UMI	500 ng	RIN: ~10	1	Upper size removal	100SR	46,318,992
TSS-seq2	with UMI	500 ng	RIN: ~10	2	Upper size removal	100SR	18,213,573
TSS-seq2	with UMI	500 ng	DV200: 82%		-	100SR	40,034,587
TSS-seq2	with UMI	500 ng	DV200: 65%		-	100SR	14,355,734
TSS-seq2	without UMI	500 ng	RIN: ~10		-	100SR	30,512,646

Supplementary Table 4. Summary statistics for randomly sampled reads

Method	Replicate	Input RNA	Sampled reads	Ribosomal reads		Reads with UMI		Uniquely mapped reads		PCR duplicate		Valid reads		TSCs (≥ 1 cpm and ≥ 2 reads)
CAGE	1	Several µg	20,000,000	6,670,520	33%	N/A	N/A	11,734,154	59%	N/A	N/A	11,734,154	59%	25,417
CAGE	2	Several µg	20,000,000	6,551,915	33%	N/A	N/A	11,819,373	59%	N/A	N/A	11,819,373	59%	25,123
Recappable-seq (with CIP treatment)	1	5 µg	20,000,000	323,376	2%	N/A	N/A	10,765,992	54%	N/A	N/A	10,765,992	54%	25,891
Recappable-seq (with CIP treatment)	2	5 µg	20,000,000	287,060	1%	N/A	N/A	9,619,631	48%	N/A	N/A	9,619,631	48%	25,215
TSS-seq (Suzuki et al. 2014)		50 µg	20,000,000	135,627	1%	N/A	N/A	13,301,607	67%	N/A	N/A	13,301,607	67%	30,528
TSS-seq1		5 µg	20,000,000	3,869	0.02%	17,628,085	88%	16,651,995	83%	3,892,795	19%	12,759,200	64%	21,685
TSS-seq1		500 ng	20,000,000	6,328	0.03%	17,302,789	87%	15,322,819	77%	4,609,706	23%	10,713,113	54%	24,714
TSS-seq1		50 ng	20,000,000	6,449	0.03%	16,859,560	84%	13,988,811	70%	12,282,634	61%	1,706,177	8.5%	33,465
TSS-seq2	1	5 µg	20,000,000	41,761	0.2%	19,194,344	96%	18,002,004	90%	6,038,408	30%	11,963,596	60%	15,812
TSS-seq2	2	5 µg	20,000,000	36,227	0.2%	19,210,552	96%	18,065,749	90%	6,633,556	33%	11,432,193	57%	15,305
TSS-seq2	1	1 µg	20,000,000	103,533	0.5%	18,894,592	94%	16,851,787	84%	5,317,304	27%	11,534,483	58%	16,201
TSS-seq2	2	1 µg	20,000,000	98,058	0.5%	18,923,460	95%	16,791,619	84%	5,362,204	27%	11,429,415	57%	16,047
TSS-seq2	1	500 ng	20,000,000	124,051	0.6%	18,779,746	94%	16,451,931	82%	5,436,479	27%	11,015,452	55%	15,722
TSS-seq2	2	500 ng	20,000,000	116,968	0.6%	18,772,066	94%	16,247,074	81%	5,380,177	27%	10,866,897	54%	16,014
TSS-seq2	1	50 ng	20,000,000	123,848	0.6%	17,553,813	88%	10,718,949	54%	5,550,242	28%	5,168,707	26%	14,838
TSS-seq2	2	50 ng	20,000,000	95,806	0.5%	17,396,169	87%	9,707,450	49%	5,160,101	26%	4,547,349	23%	15,157
TSS-seq2	1	5 ng	20,000,000	56,611	0.3%	15,959,801	80%	2,254,696	11%	1,645,694	8.2%	609,002	3.0%	11,164
TSS-seq2	2	5 ng	20,000,000	26,155	0.1%	15,958,614	80%	1,284,316	6.4%	857,902	4.3%	426,414	2.1%	9,649
TSS-seq2 (lower size removal)	1	50 ng	20,000,000	180,696	0.9%	18,695,791	93%	16,687,972	83%	9,529,602	48%	7,158,370	36%	-
TSS-seq2 (lower size removal)	1	5 ng	20,000,000	249,146	1.2%	17,608,998	88%	10,999,744	55%	9,991,550	50%	1,008,194	5.0%	-

Supplementary Table 5. Performance evaluation for each method

	CPM threshold with the highest F1 score (>=)	Precision	Sensitivity	F1 score
CAGE_R1	1.87	0.86	0.65	0.74
CAGE_R2	1.52	0.84	0.66	0.74
Recap_R1	1.39	0.81	0.42	0.55
Recap_R2	1.25	0.81	0.44	0.57
original_TSS	2.18	0.65	0.40	0.50
TSS2_5ug_R1	0.42	0.89	0.63	0.74
TSS2_5ug_R2	0.44	0.90	0.61	0.72
TSS2_1ug_R1	0.52	0.89	0.63	0.74
TSS2_1ug_R2	0.44	0.88	0.63	0.74
TSS2_500ng_R1	0.54	0.90	0.62	0.73
TSS2_500ng_R2	0.55	0.90	0.62	0.73
TSS2_50ng_R1	0.77	0.91	0.54	0.68
TSS2_50ng_R2	0.66	0.88	0.54	0.67
TSS2_5ng_R1	3.28	0.92	0.32	0.48
TSS2_5ng_R2	4.69	0.94	0.28	0.43

Supplementary Table 6. Estimated cost for library preparation of TSS-seq2

Product names	Manufacturers	Catalog numbers	Unit Sizes	Price(USD)	requirements/reaction	Cost(USD)/reaction
Alkaline Phosphatase (*E. coli* C75)	Takara Bio	2120B	500 µl	431	2.5 µl	2.155
Rnasin Plus Ribonuclease Inhibitor	Promega	N2615	250 µl	394	5.4 µl	8.510
mRNA Decapping Enzyme	NEB	M0608S	20 µl	103	1 µl	5.150
T4 RNA Ligase2	NEB	M0239L	75 µl	345	2.5 µl	11.500
KAPA HiFi HotStart ReadyMix	Roche	KK2601	1250 µl	136	25 µl	2.720
10mM dNTP	NEB	N0447S	800 µl	69	2.5 µl	0.216
Maxima H Minus Reverse Transcriptase	Thermo Fisher Scientific	EP0752	50 µl	339	2 µl	13.540
Betaine (5 M)	Sigma-Aldrich	B0300-1VL	1500 µl	28.5	10 µl	0.190
DTT (1M)	FUJIFILM Wako Chemicals	040-33873	1000 µl	39	0.25 µl	0.010
RNA Clean XP	Beckman Coulter	A63987	40,000 µl	901.2	310 µl	6.984
SPRI Select	Beckman Coulter	B23318	60,000 µl	1,640	56 µl	1.531
D(-)-Sorbitol	FUJIFILM Wako Chemicals	194-03752	25 g	20	0.0045 g	0.004
Trehalose Dihydrate	FUJIFILM Wako Chemicals	202-18452	25 g	37	0.001875 g	0.003
Ethanol	FUJIFILM Wako Chemicals	057-00456	500 ml	25	1.76 ml	0.088
2-mercaptoethanol	FUJIFILM Wako Chemicals	131-14572	25000 ul	29	0.036 µl	0.000
1 M Tris-HCl (pH7)	NIPPON GENE	311-90411	100000 µl	64*	5 µl	0.003
1 M Tris-HCl (pH7.5)	NIPPON GENE	316-90221	100000 µl	64*	0.03 µl	0.000
0.5 M EDTA (pH8)	NIPPON GENE	311-90075	500000 µl	72*	0.0006 µl	0.000
5M NaCl	Thermo Fisher Scientific	AM9760G	100000 µl	52.5	0.03 µl	0.000
Nuclease-Free Water	Thermo Fisher Scientific	AM9930	500 ml	87	0.5 ml	0.087
PEG8000	Sigma-Aldrich	89510-250G-F	250 g	53.3	0.0025 g	0.001
Splint Oligo	FASMAC		7.6 nmol	230*	0.2 nmol	6.041
Capping oligo	FASMAC		9.3 nmol	154*	0.1 nmol	1.658
RT primer	FASMAC		10 nmol	9*	0.5 nmol	0.452
i7 primer	FASMAC		10 nmol	16*	0.015 nmol	0.024
i5 primer	FASMAC		10 nmol	15*	0.015 nmol	0.022
Agilent DNA 7500 Kit	Agilent Technologies	5067-1506	300 samples	1,545.83	1 sample	5.153

*The reagents with unknown U.S. prices were converted to Japanese prices using an exchange rate of 138 yen per U.S. dollar.
All prices were investigated on 19 July 2023

**Total cost per reaction**	**66.04 USD**
**Initial cost**	**6,897 USD**

Supplementary Table 7. Sequencing statistics for TSS−seq2 of plant samples

Sample Name	Read length	Total reads	Ribosomal reads		Reads with UMI		Uniquely mapped reads		PCR duplicates		Valid reads	
Nbe_R1	100SR	39,372,496	3,782	0.01%	37,190,515	94%	28,035,302	71%	7,706,278	20%	20,329,024	52%
Nbe_R2	100SR	36,226,958	4,421	0.01%	34,234,838	95%	25,374,883	70%	7,411,234	20%	17,963,649	50%
Aha_R1	100SR	43,733,460	8,689	0.02%	40,634,754	93%	29,113,462	67%	22,731,503	52%	6,381,959	15%
Aha_R2	100SR	50,529,851	6,923	0.01%	47,384,839	94%	36,498,491	72%	30,100,015	60%	6,398,476	13%
Lja_R1	100SR	141,978,816	74,878	0.05%	135,046,596	95%	90,003,055	63%	66,699,059	47%	23,303,996	16%
Lja_R2	100SR	165,194,728	99,024	0.06%	156,939,523	95%	100,129,213	61%	77,114,197	47%	23,015,016	14%
Pja_ctrl_R1	150PE*	43,726,797	3,466	0.01%	41,456,074	95%	32,359,933	74%	11,021,443	25%	21,338,490	49%
Pja_ctrl_R2	150PE*	37,914,895	3,499	0.01%	36,014,578	95%	28,663,229	76%	9,193,552	24%	19,469,677	51%
Pja_SyA_R1	150PE*	74,583,906	5,997	0.01%	70,674,507	95%	55,082,685	74%	21,490,503	29%	33,592,182	45%
Pja_SyA_R2	150PE*	63,684,193	5,534	0.01%	60,266,993	95%	46,559,546	73%	17,082,342	27%	29,477,204	46%

*For libraries of P. japonicum, only read1 was used in this study.

Supplementary Table 8. Sequencing statistics for RNA-seq of plant samples

Sample Name	Read length	Total number of read pairs	Uniquely mapped reads	
Nbe_R1	150PE	86,751,670	69,136,589	80%
Nbe_R2	150PE	89,926,346	70,753,851	79%
Aha_R1	150PE	81,942,807	61,254,911	75%
Aha_R2	150PE	74,213,114	55,875,882	75%
Lja_R1	150PE	53,328,098	37,685,375	71%
Lja_R2	150PE	71,520,418	48,422,302	68%
Pja_ctrl_R1	150PE	85,266,585	72,507,905	85%
Pja_ctrl_R2	150PE	81,898,345	66,769,275	82%
Pja_SyA_R1	150PE	82,949,650	70,459,447	85%
Pja_SyA_R2	150PE	80,773,970	65,444,046	81%

Supplementary Table 9. Assembled transcript statistics for plant RNA-seq data

Species	Transcripts	Know transcripts	Novel transcripts
Nicotiana benthamiana	62,425	17,532	44,893
Arabidopsis halleri	31,597	13,781	17,816
Lotus japonicus	36,929	17,523	19,406
Phtheirospermum japonicum	38,381	6,518	31,863

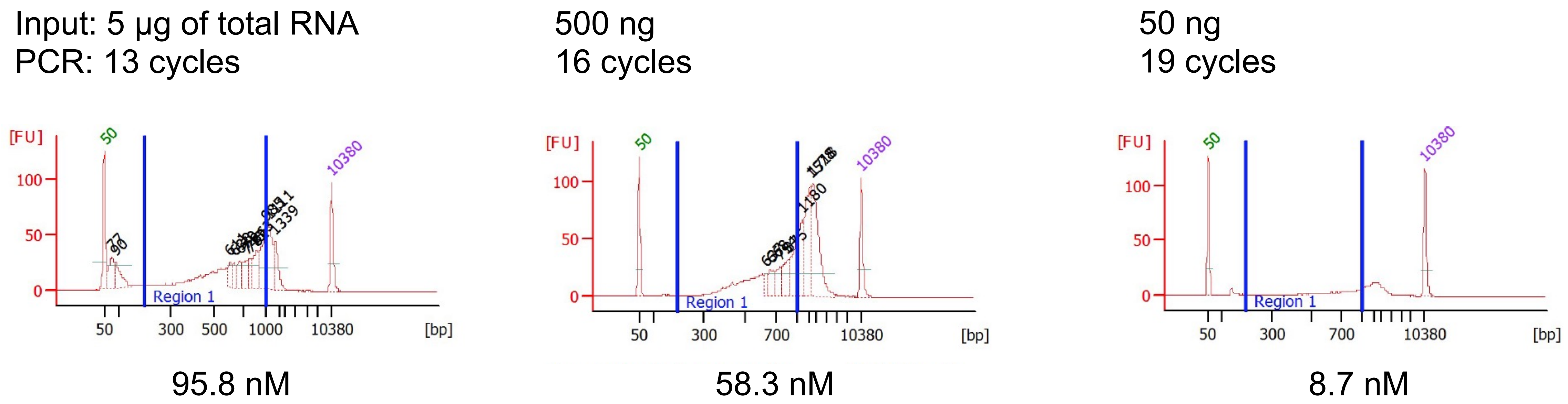

Supplementary Figure 1. TSS-seq1 libraries

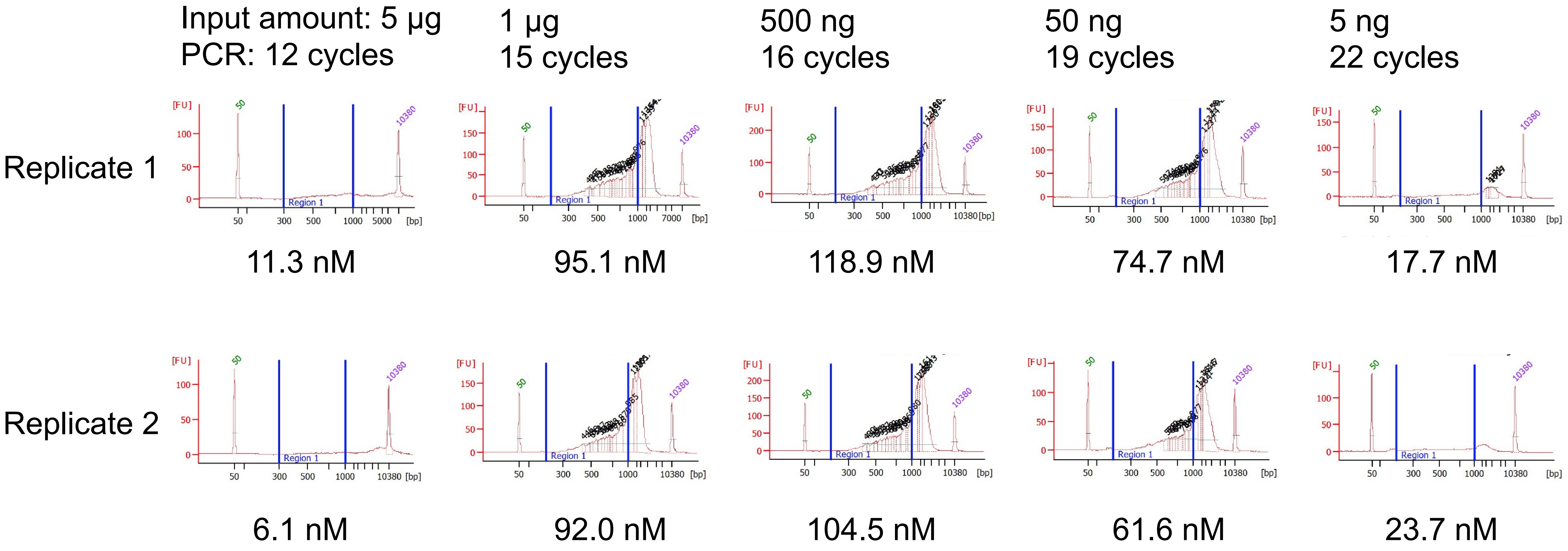

Supplementary Figure 2. TSS-seq2 libraries

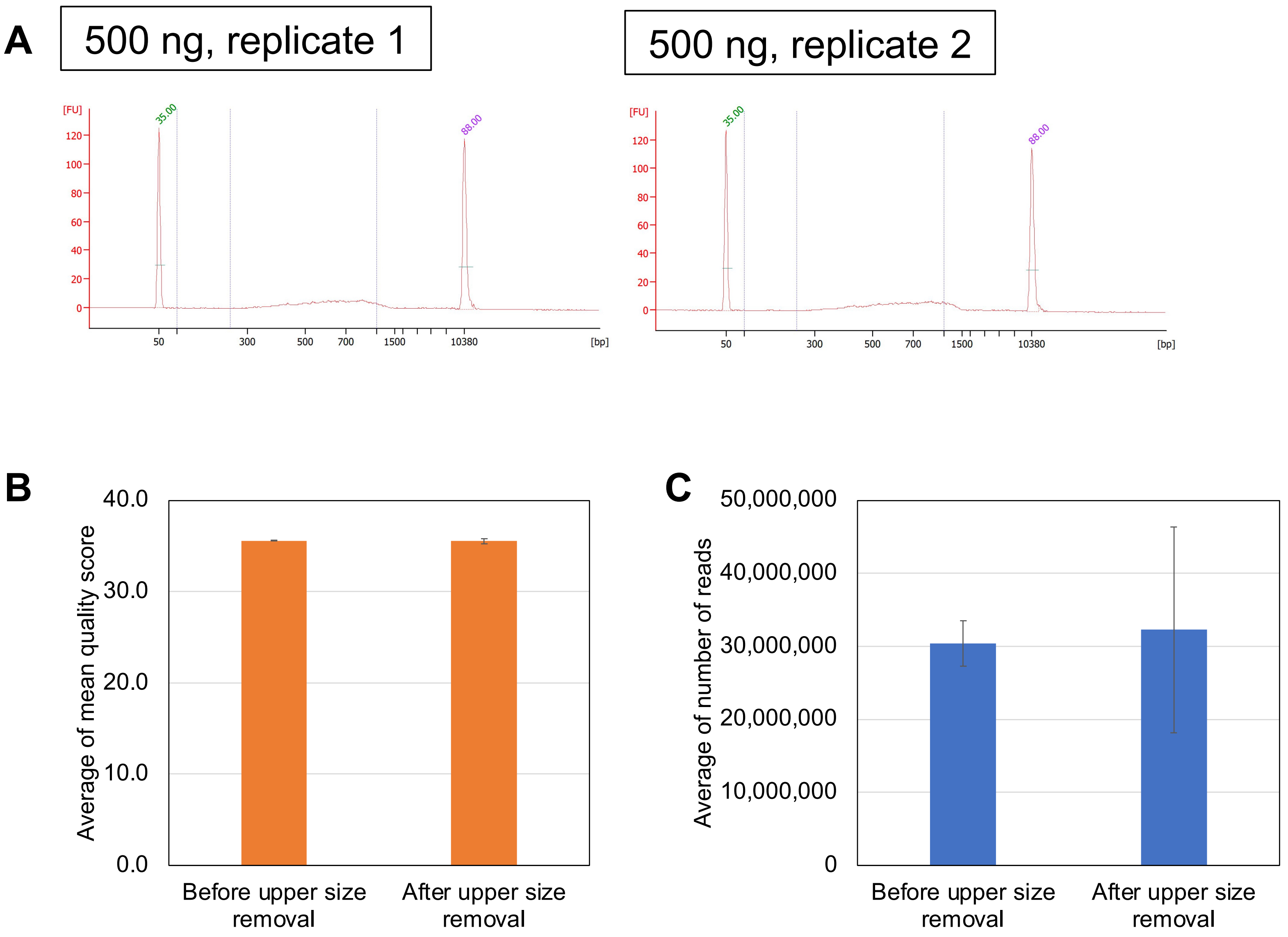

Supplementary Figure 3. Qualities and yields of sequencing before and after the upper size removal

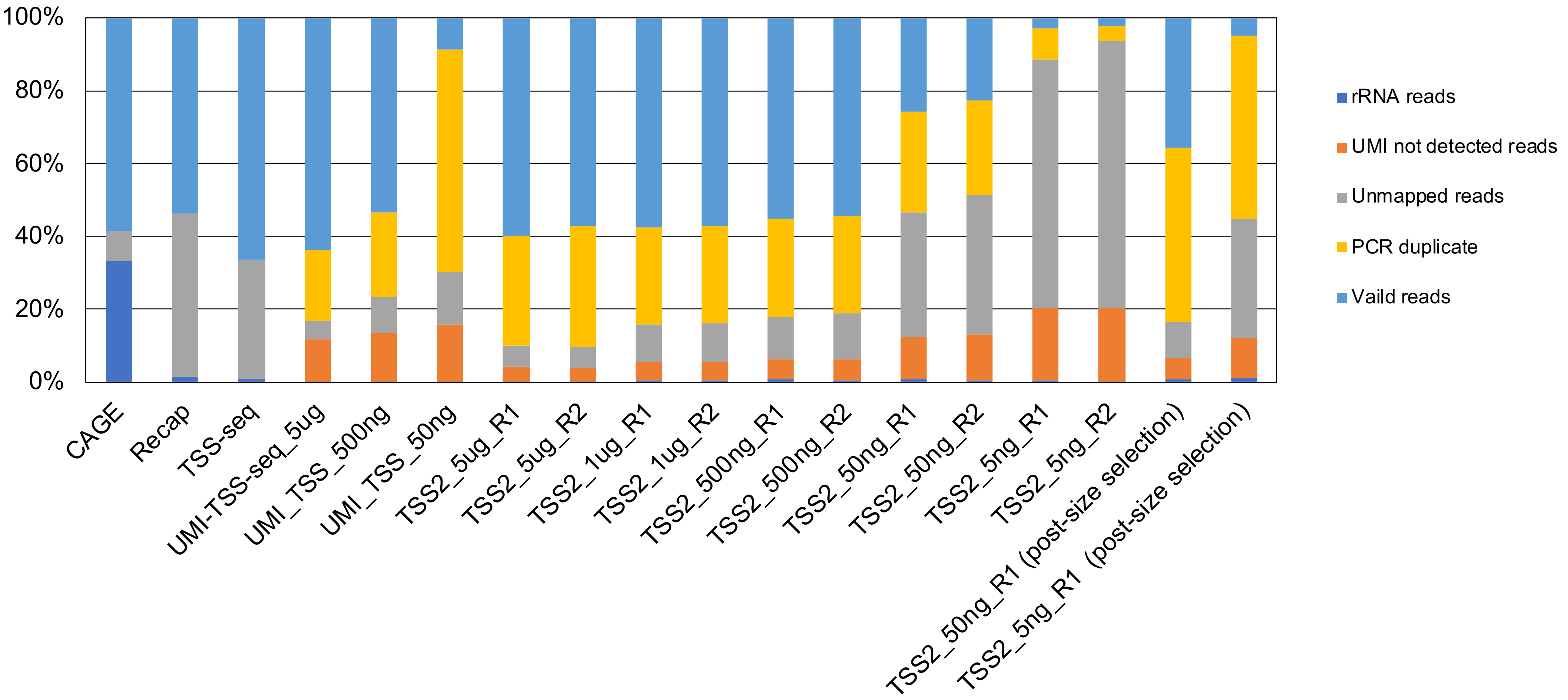

Supplementary Figure 4. Breakdown of reads of libraries prepared by each method

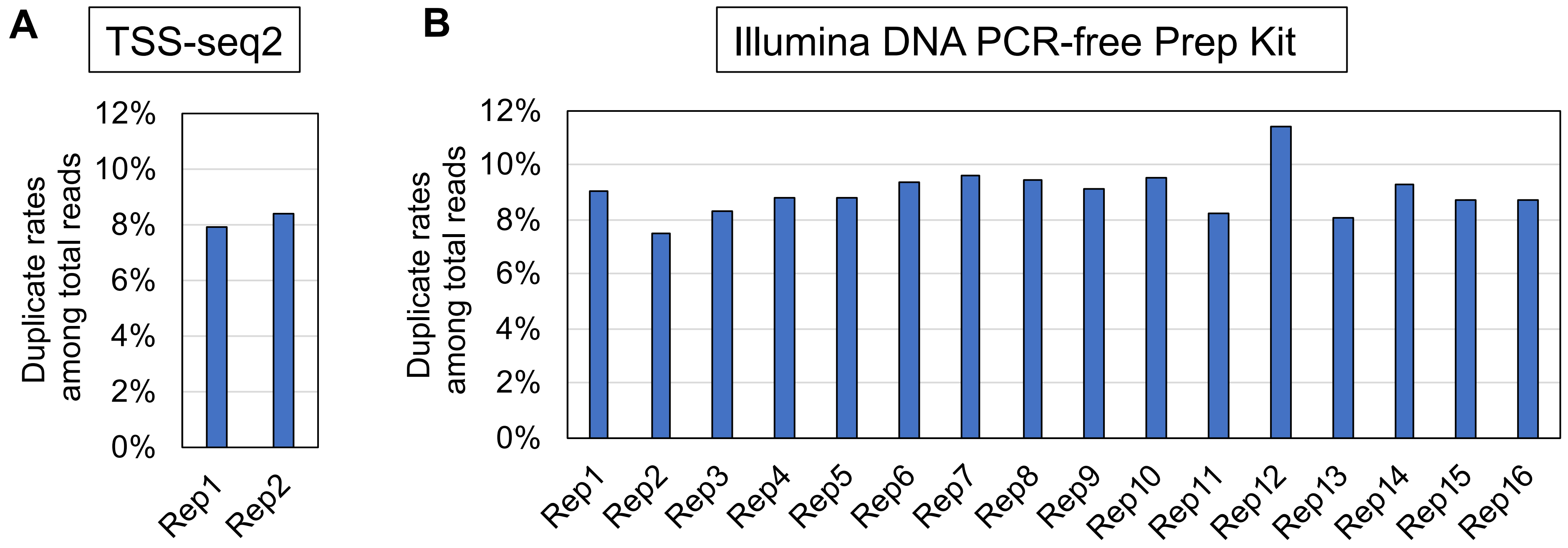

Supplementary Figure 5. Optical duplicate rates of TSS-seq2 and the PCR-free WGS kit

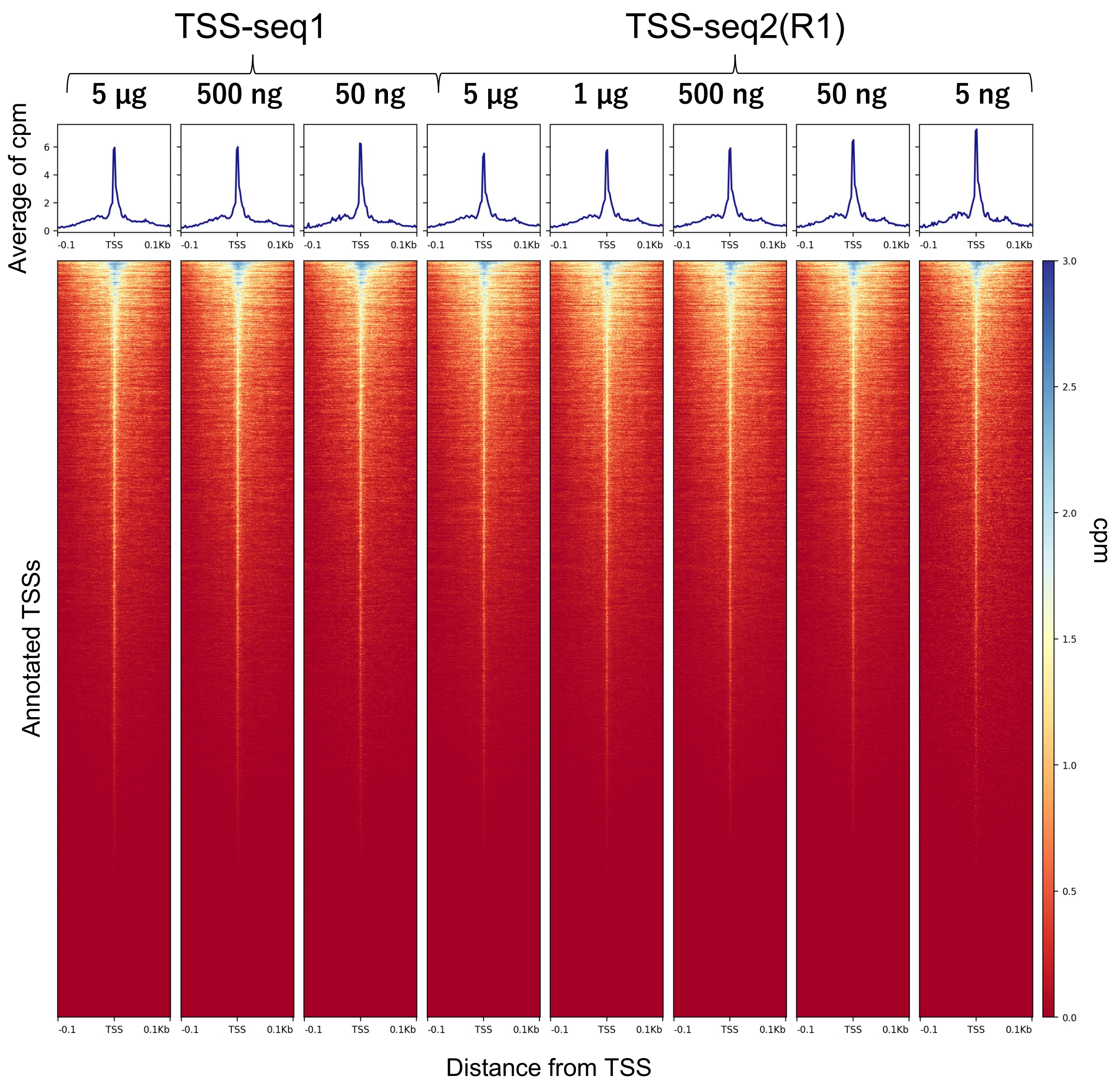

Supplementary Figure 6. Heatmap of expression levels of TSS measured by TSS-seq 1 and 2 around annotated TSSs

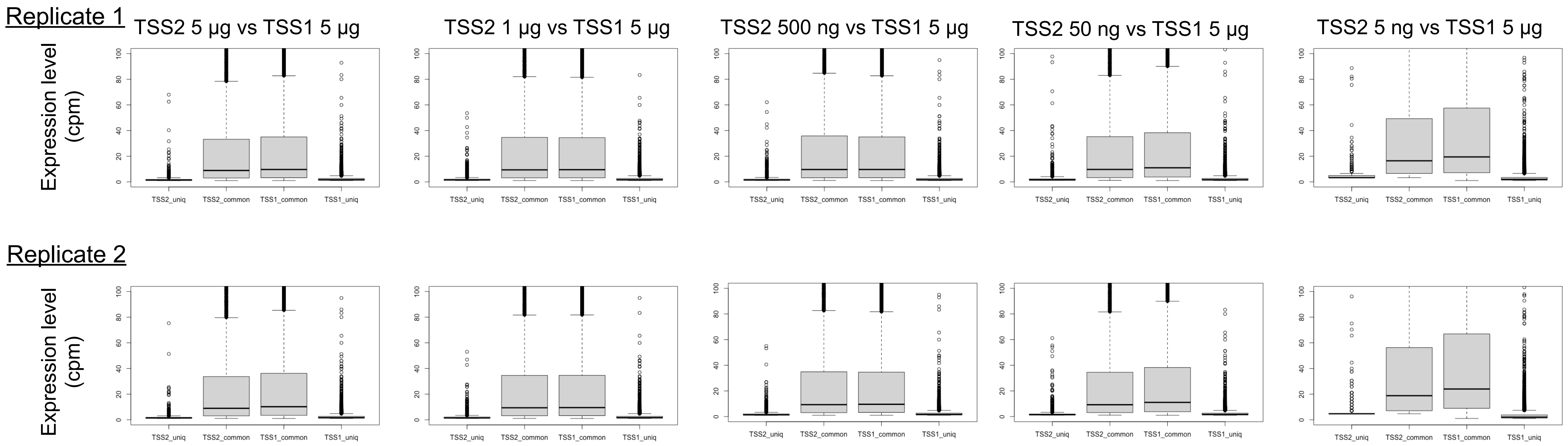

Supplementary Figure 7. Expression levels of TSCs for each category of overlap patterns between TSS-seq1 and 2

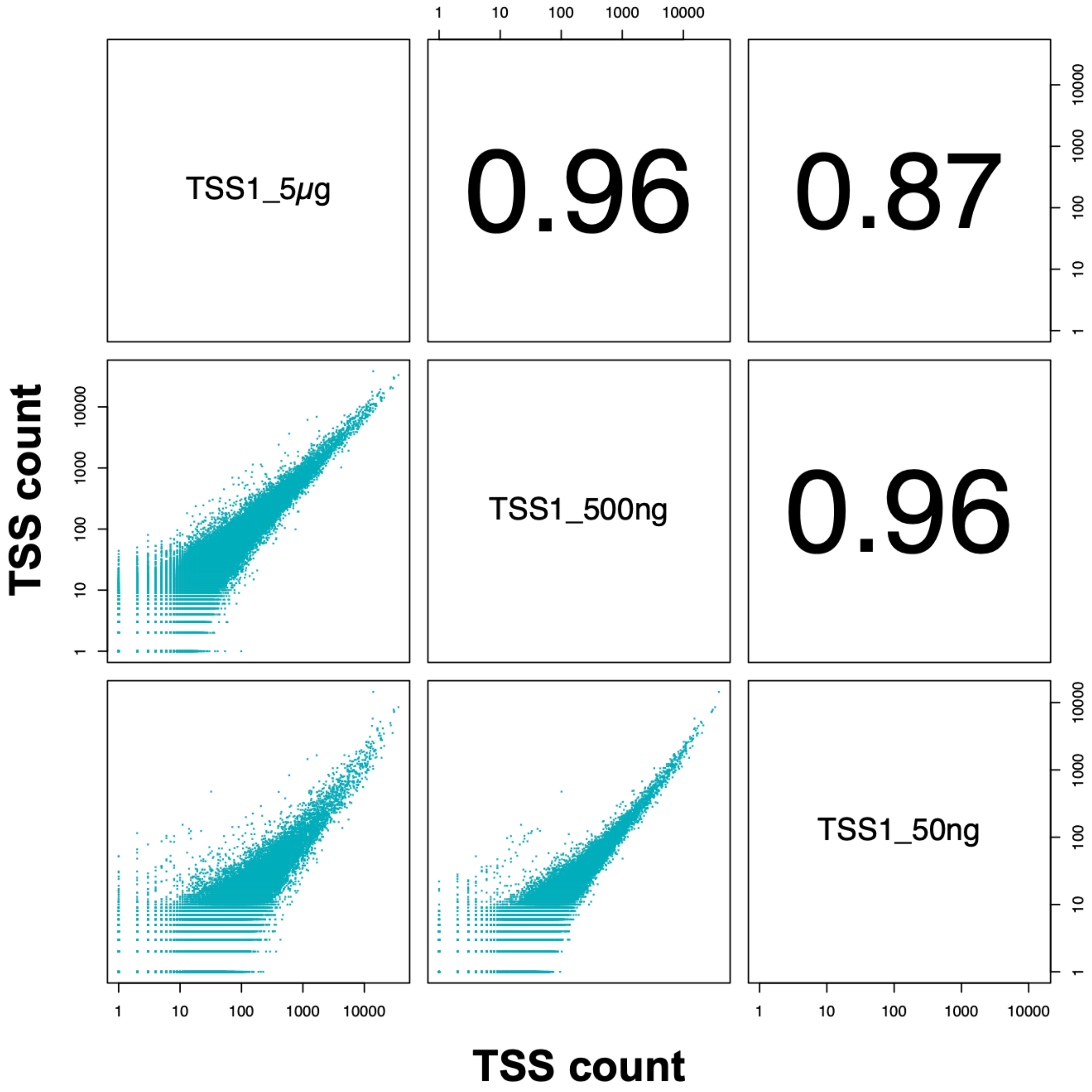

Supplementary Figure 8. Correlation of TSS counts among TSS-seq1 datasets

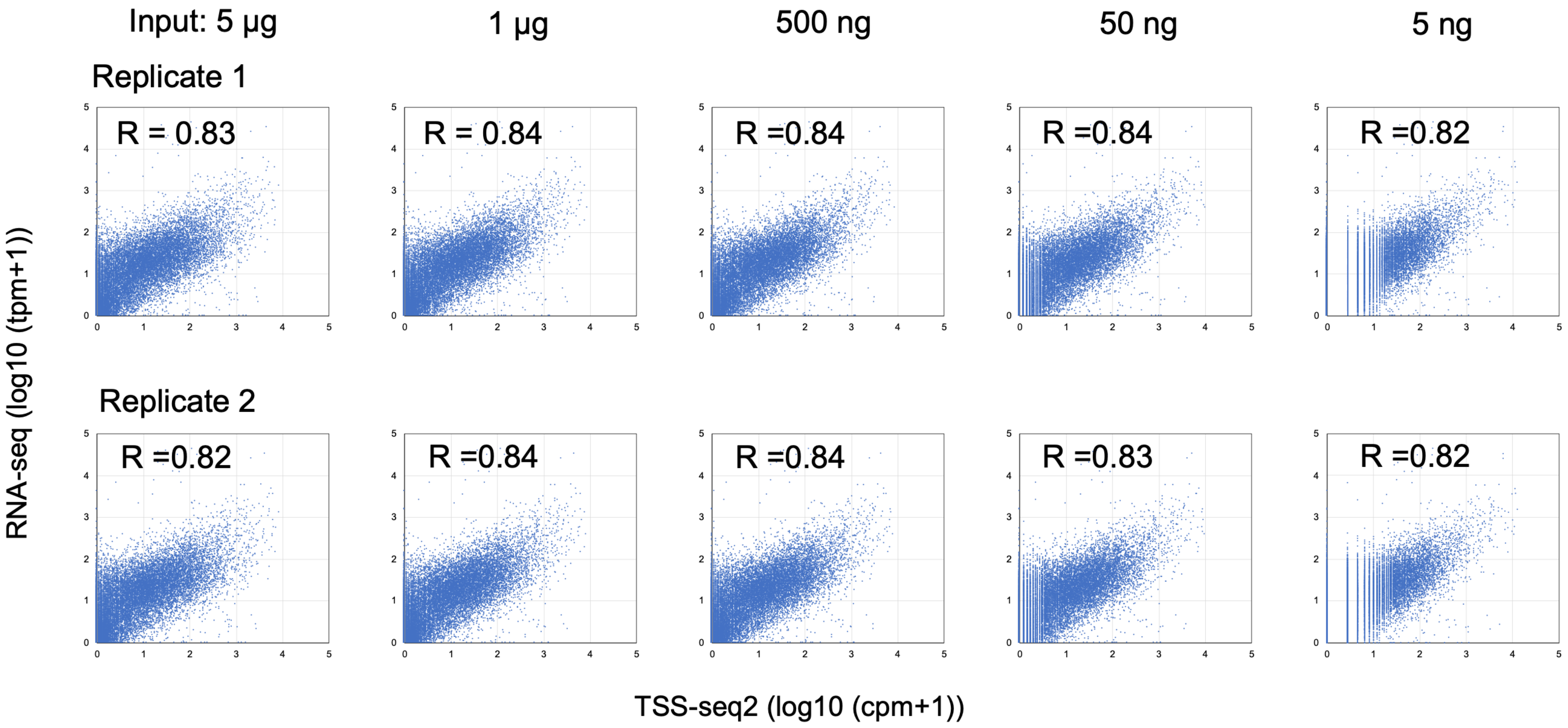

Supplementary Figure 9. Comparison of expression levels estimated by RNA-seq and TSS-seq2

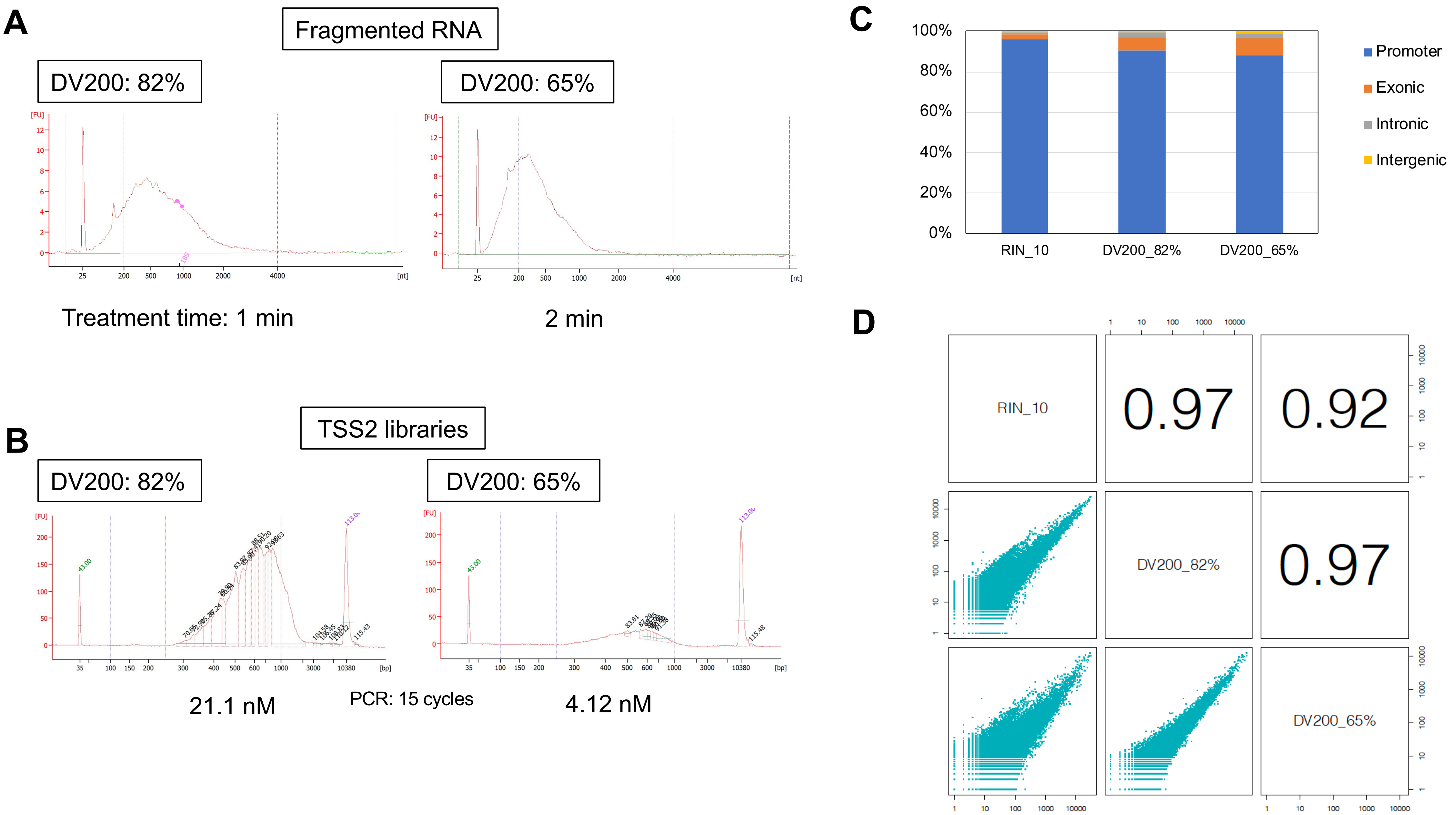

Supplementary Figure 10. TSS-seq2 from fragmented RNA

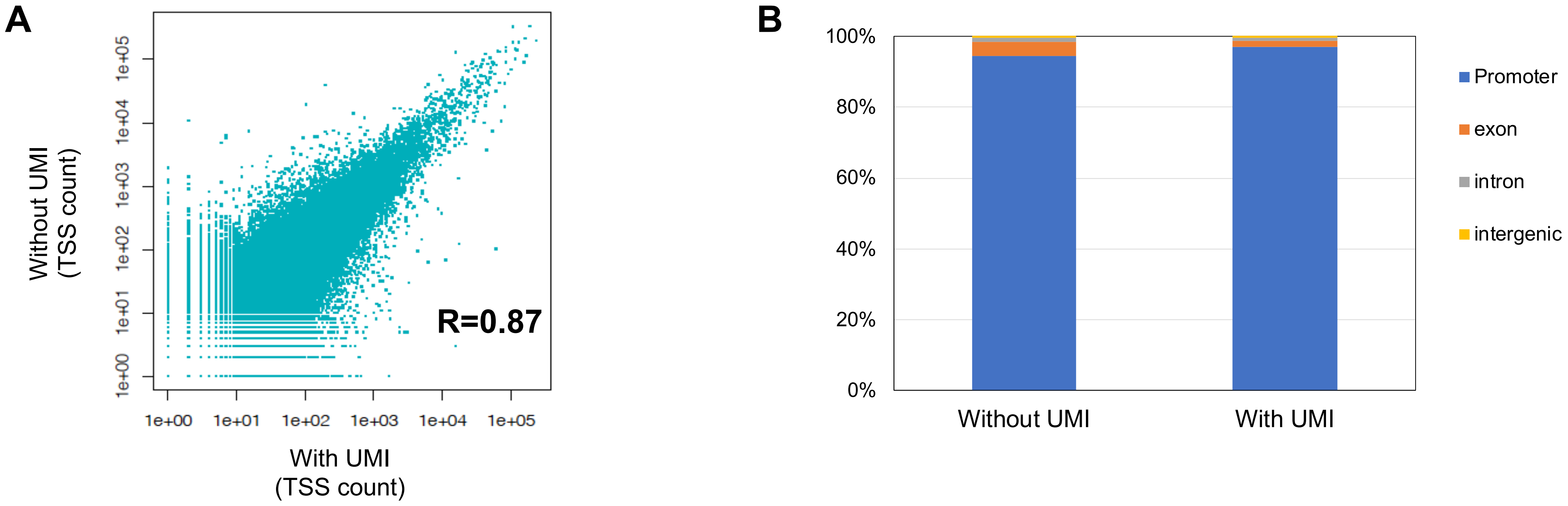

Supplementary Figure 11. Comparison of libraries prepared using splint adapters with/without UMI sequences

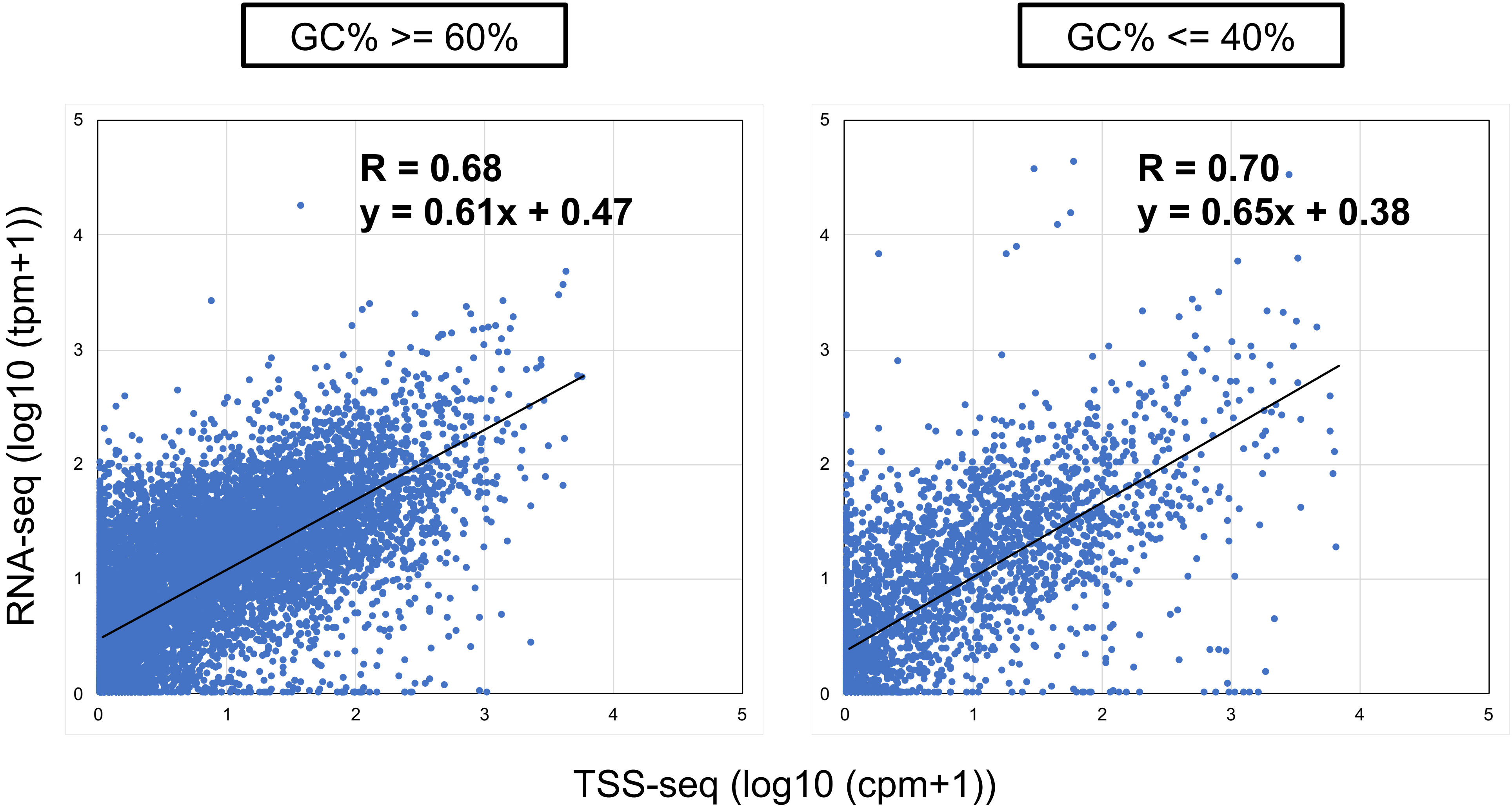

Supplementary Figure 12. Comparison of GC-rich and AT-rich TSCs

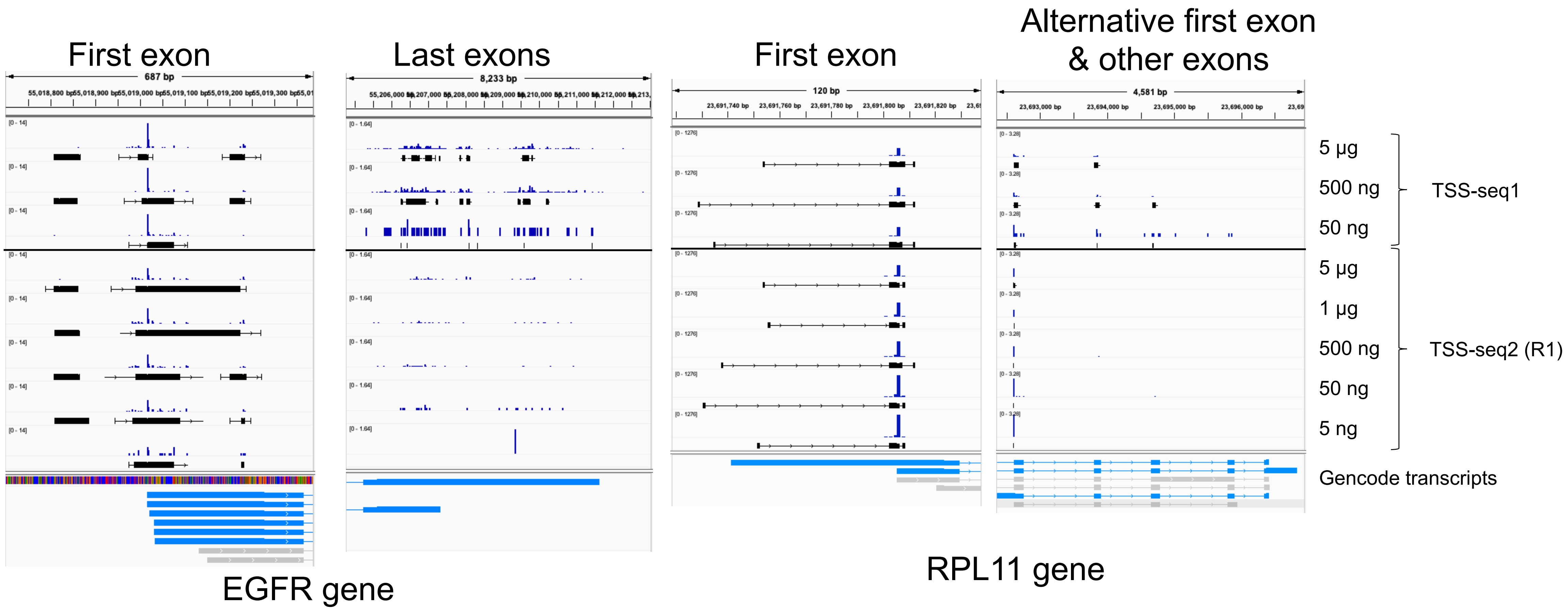

Supplementary Figure 13. Reduction in misdetections of exonic TSCs by TSS-seq2

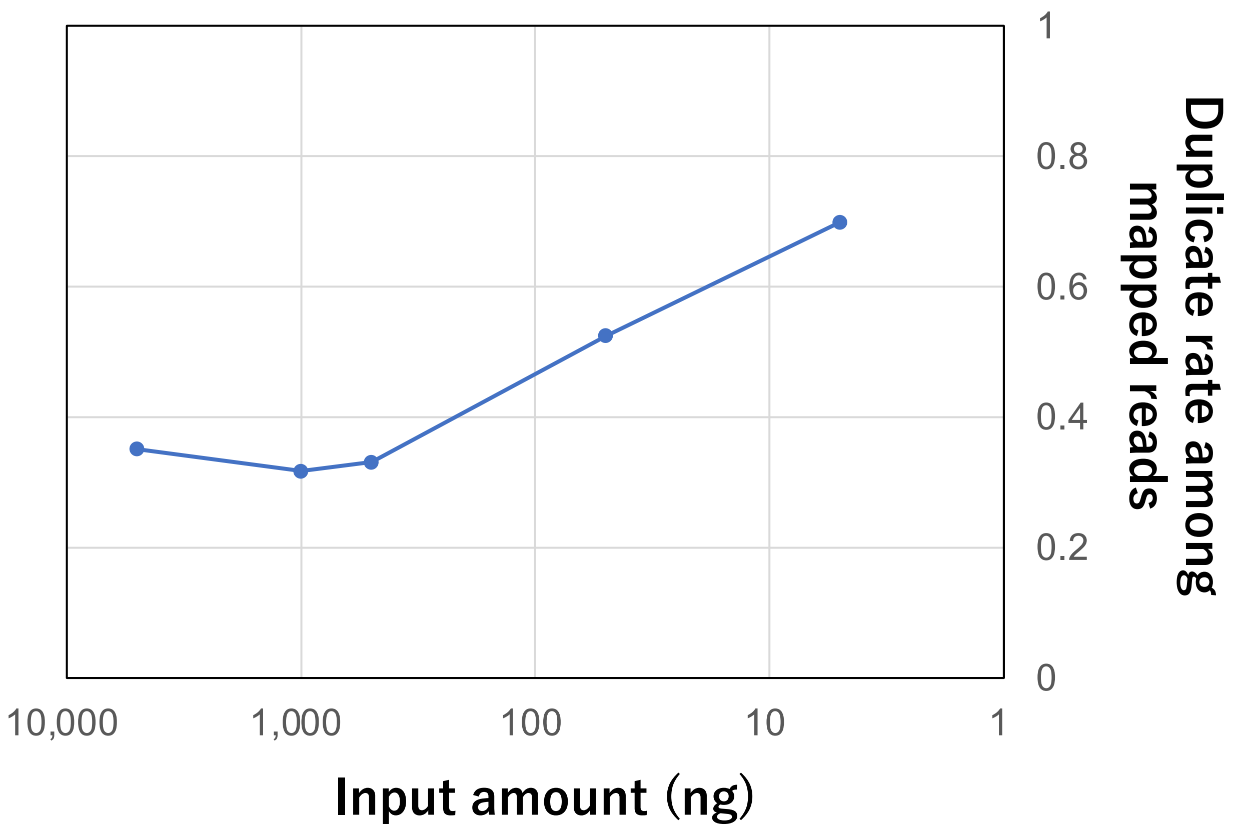

Supplementary Figure 14. PCR duplicate rate in TSS-seq2 prepared from various input amounts

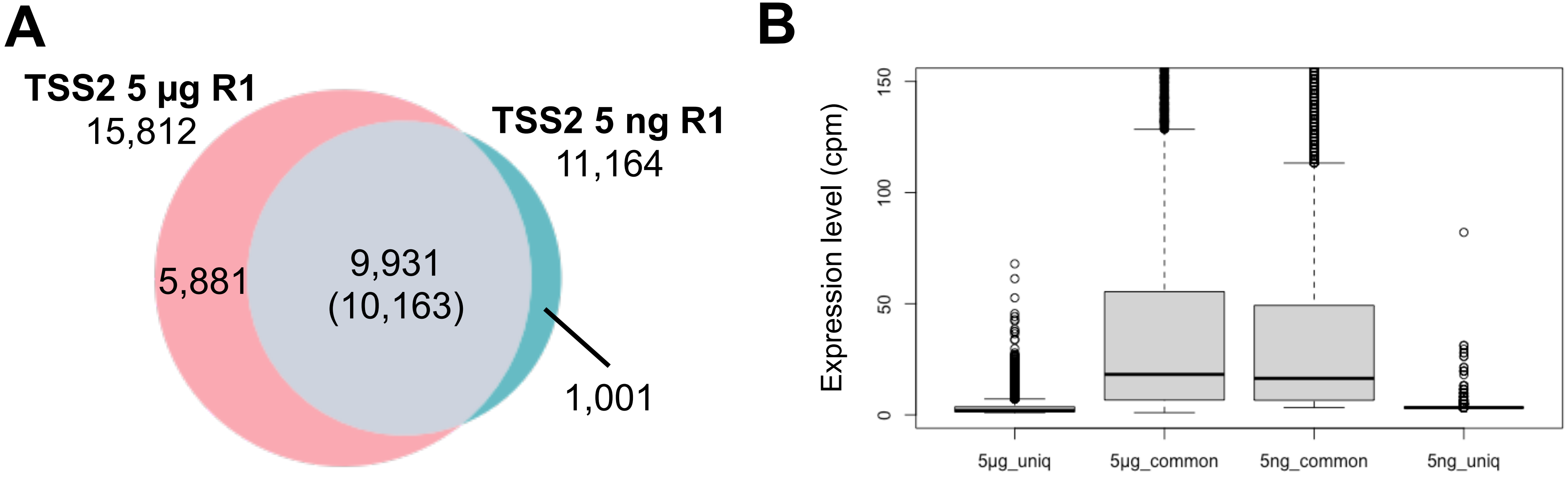

Supplementary Figure 15. Comparison of TSS-seq2 datasets with 5 ng and 5 µg input

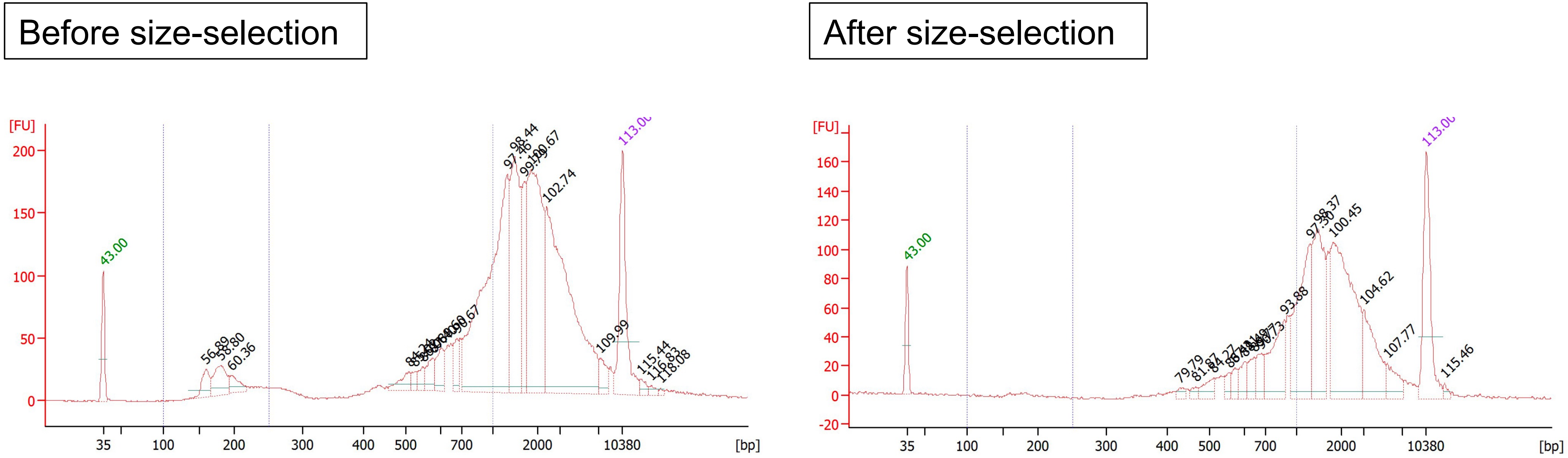

Supplementary Figure 16. Typical views of TSS-seq2 libraries from small inputs before and after additional size selection

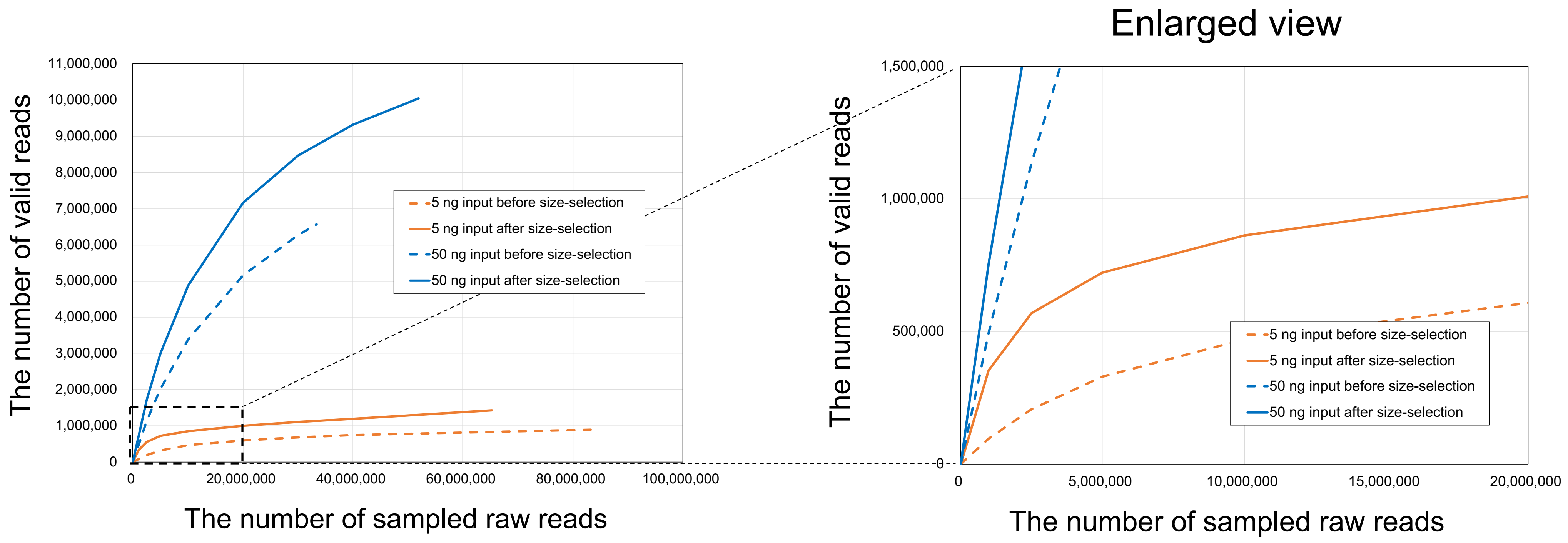

Supplementary Figure 17. Saturation curve of sequencing depth in 50 and 5 ng inputs after size selection

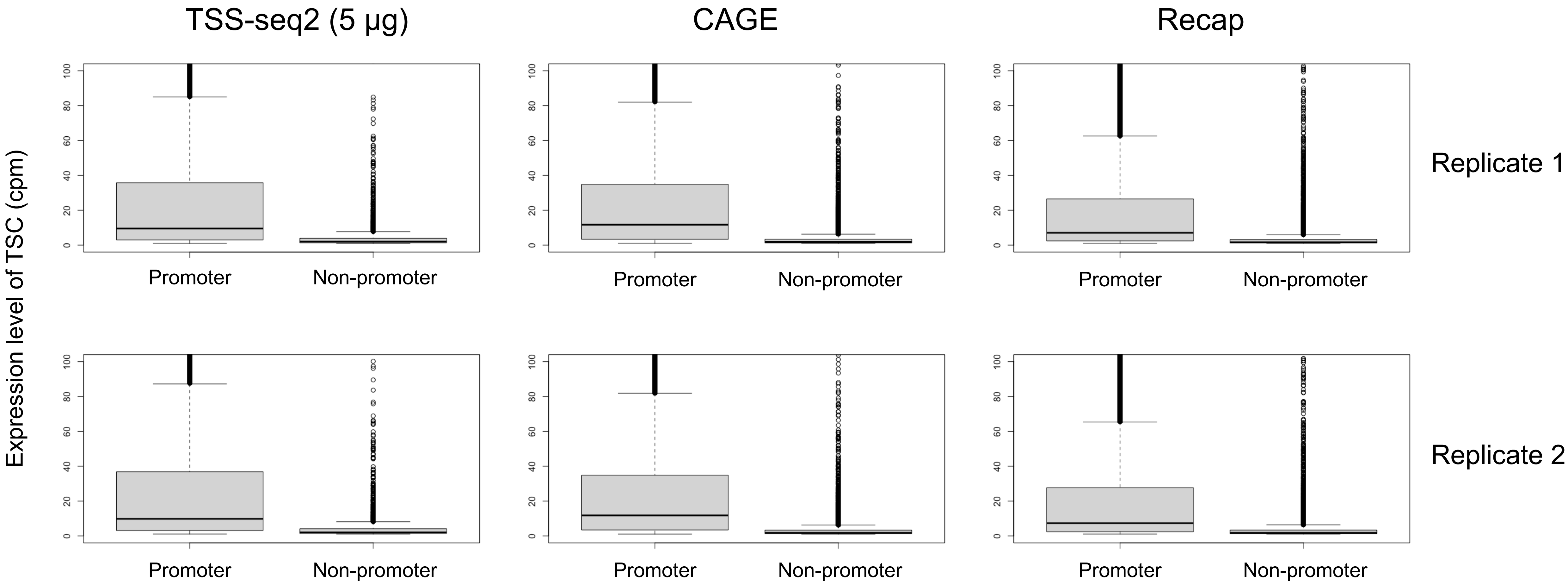

Supplementary Figure 18. Expression levels of TSCs that overlap and do not overlap with promoters

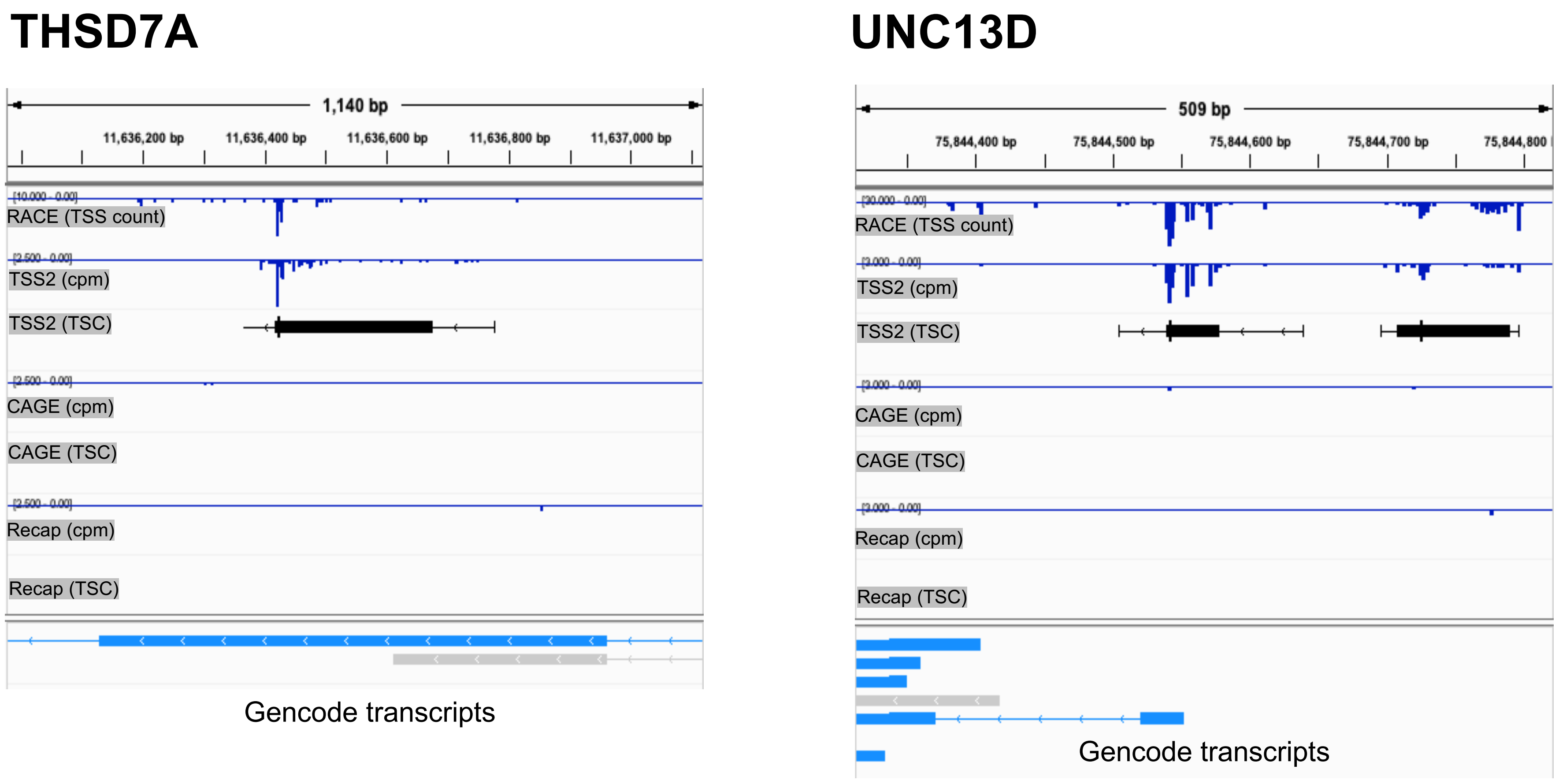

Supplementary Figure 19. RACE analysis of TSCs uniquely detected by TSS-seq2

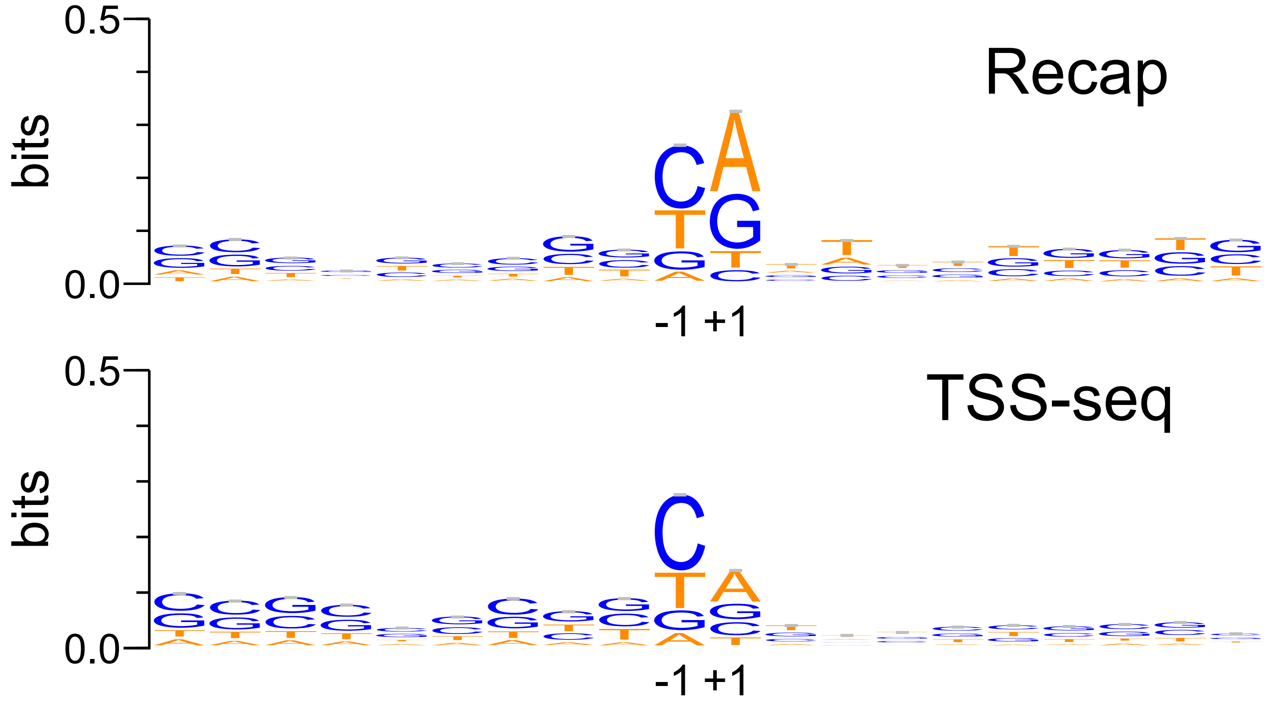

Supplementary Figure 20. Sequence logos for recappable-seq and TSS-seq

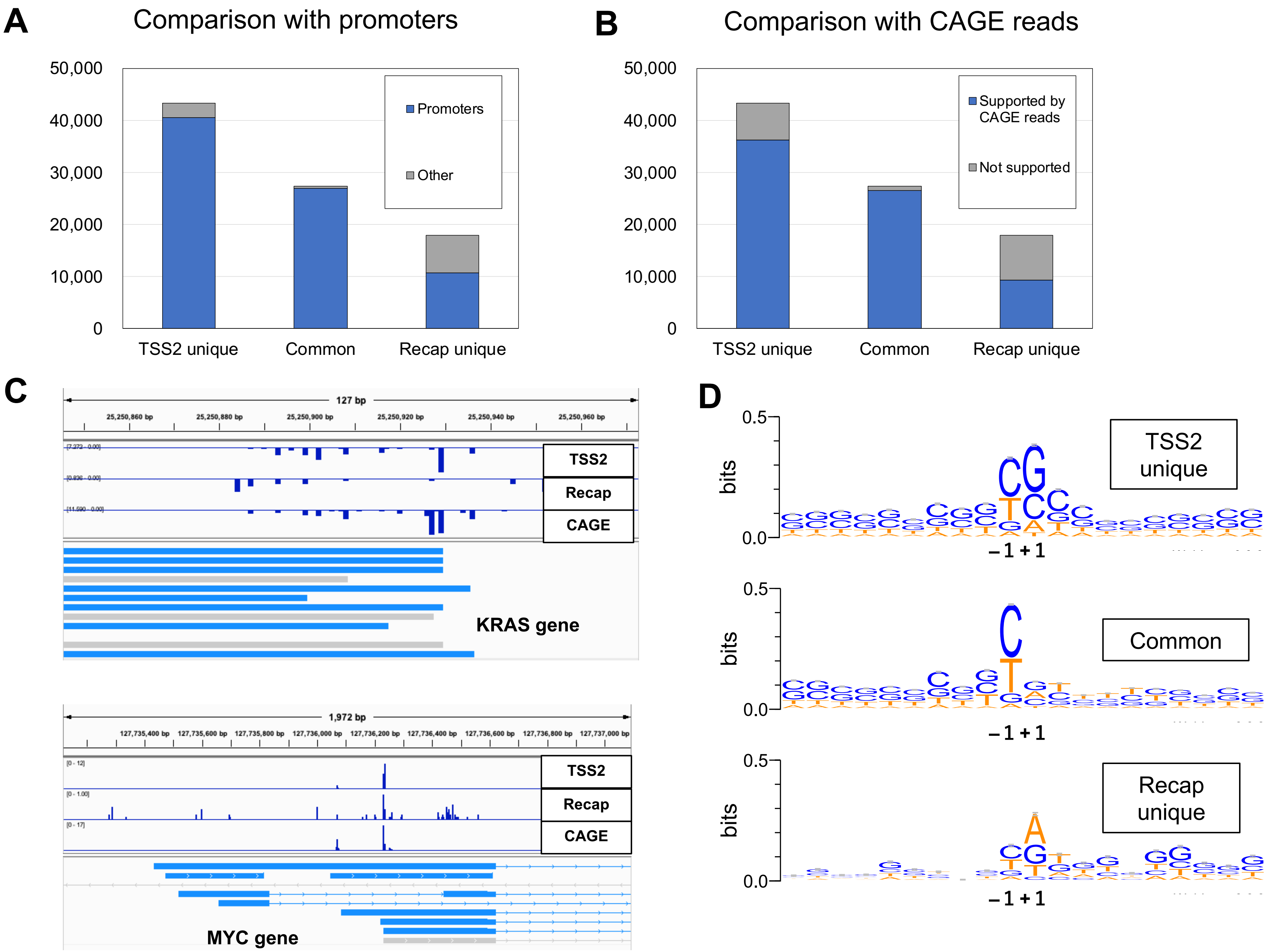

Supplementary Figure 21. Comparison of TSS-seq2 and Recappable-seq at a 1 bp resolution

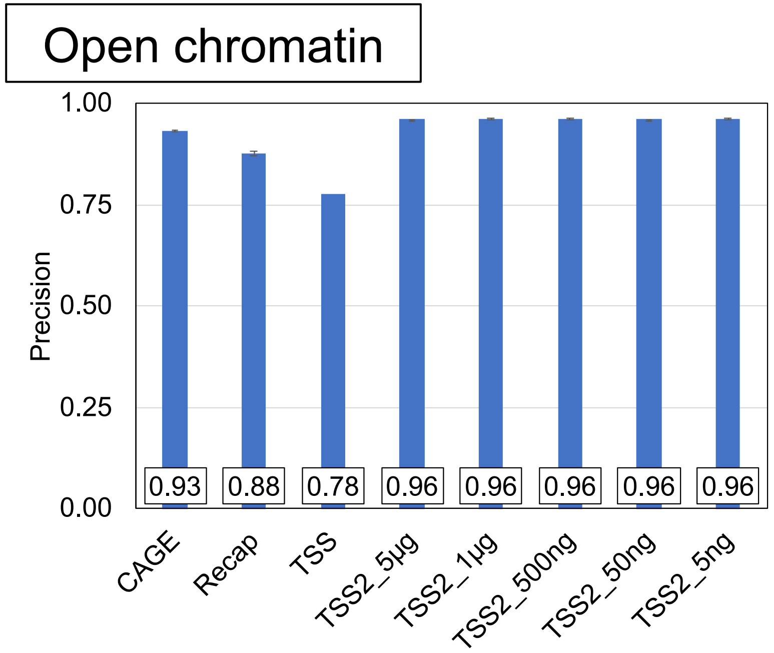

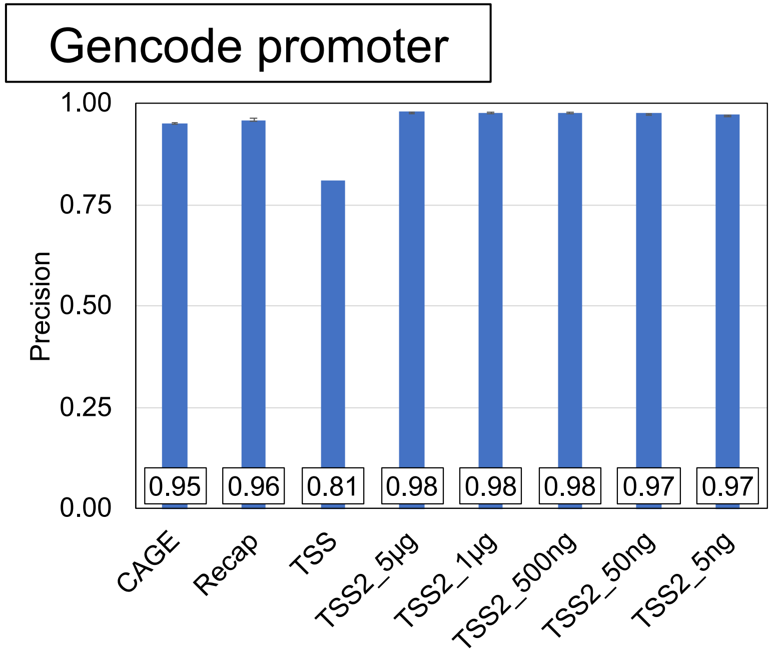

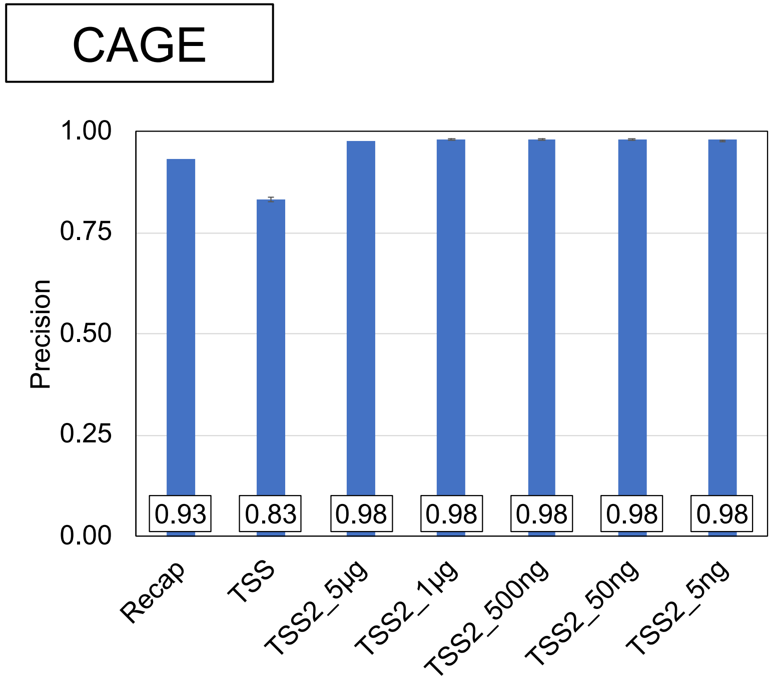

Supplementary Figure 22. Evaluation of precision with various criteria

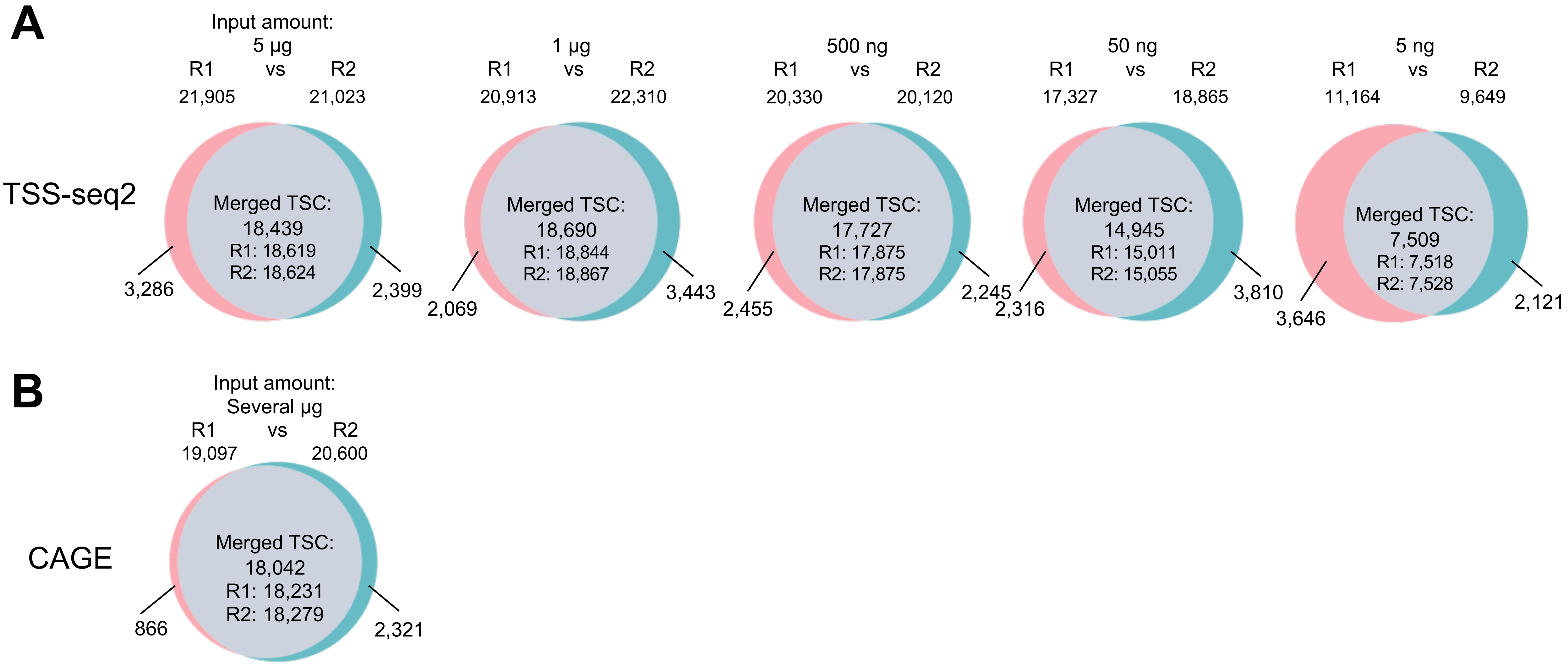

Supplementary Figure 23. Comparison between TSS-seq2 and CAGE replicates

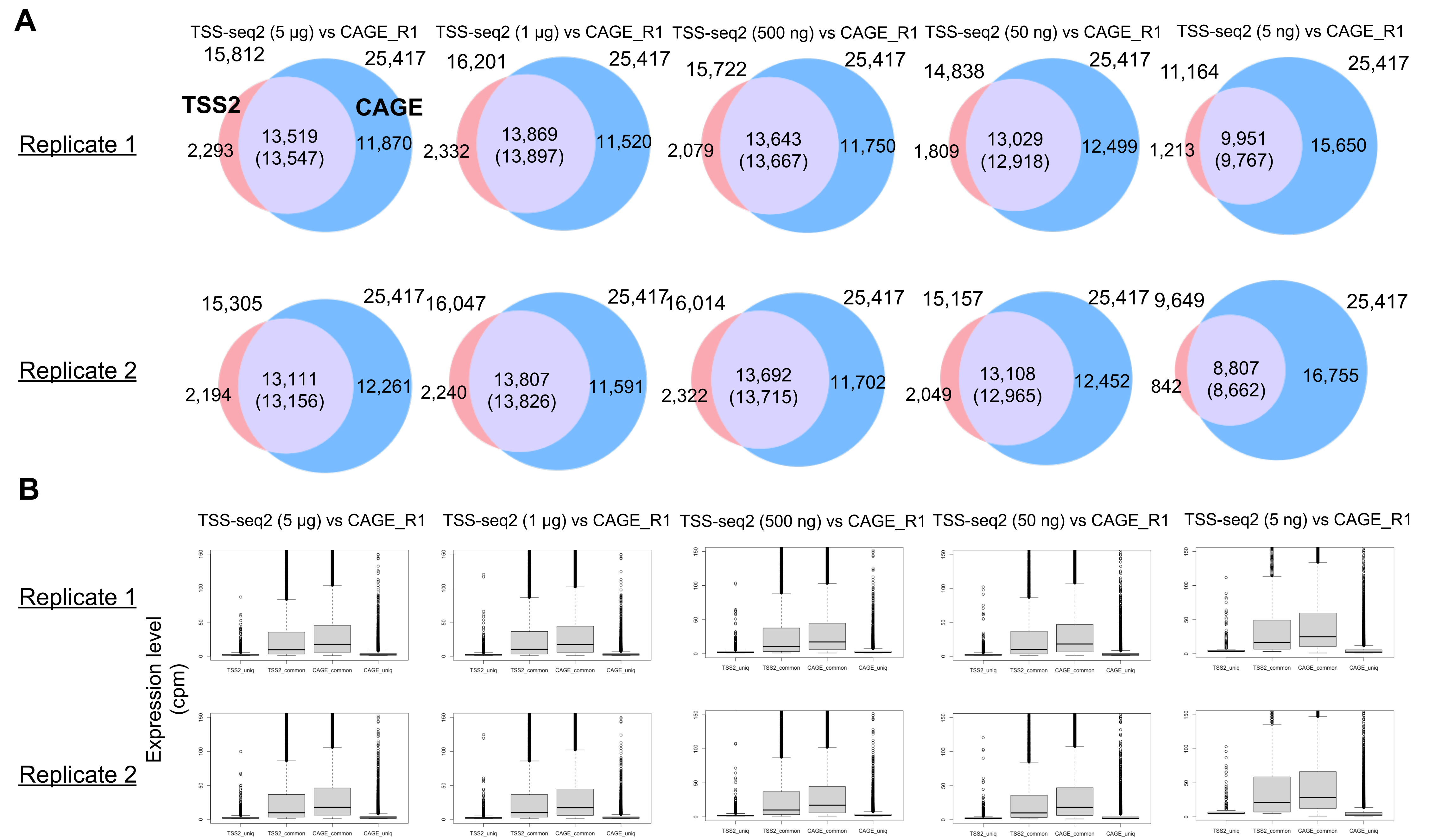

Supplementary Figure 24. Comparison of TSCs between TSS-seq2 and CAGE filtered at a threshold of ≥1 cpm and ≥2 read per TSC

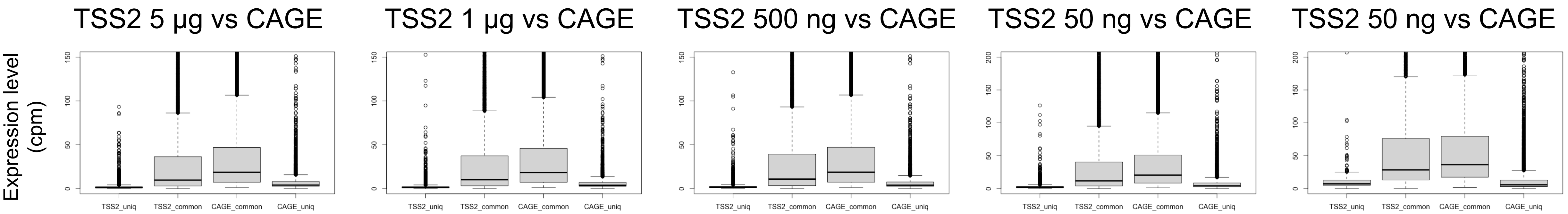

Supplementary Figure 25. Expression levels of TSCs for each category of overlap patterns between TSS-seq2 and CAGE

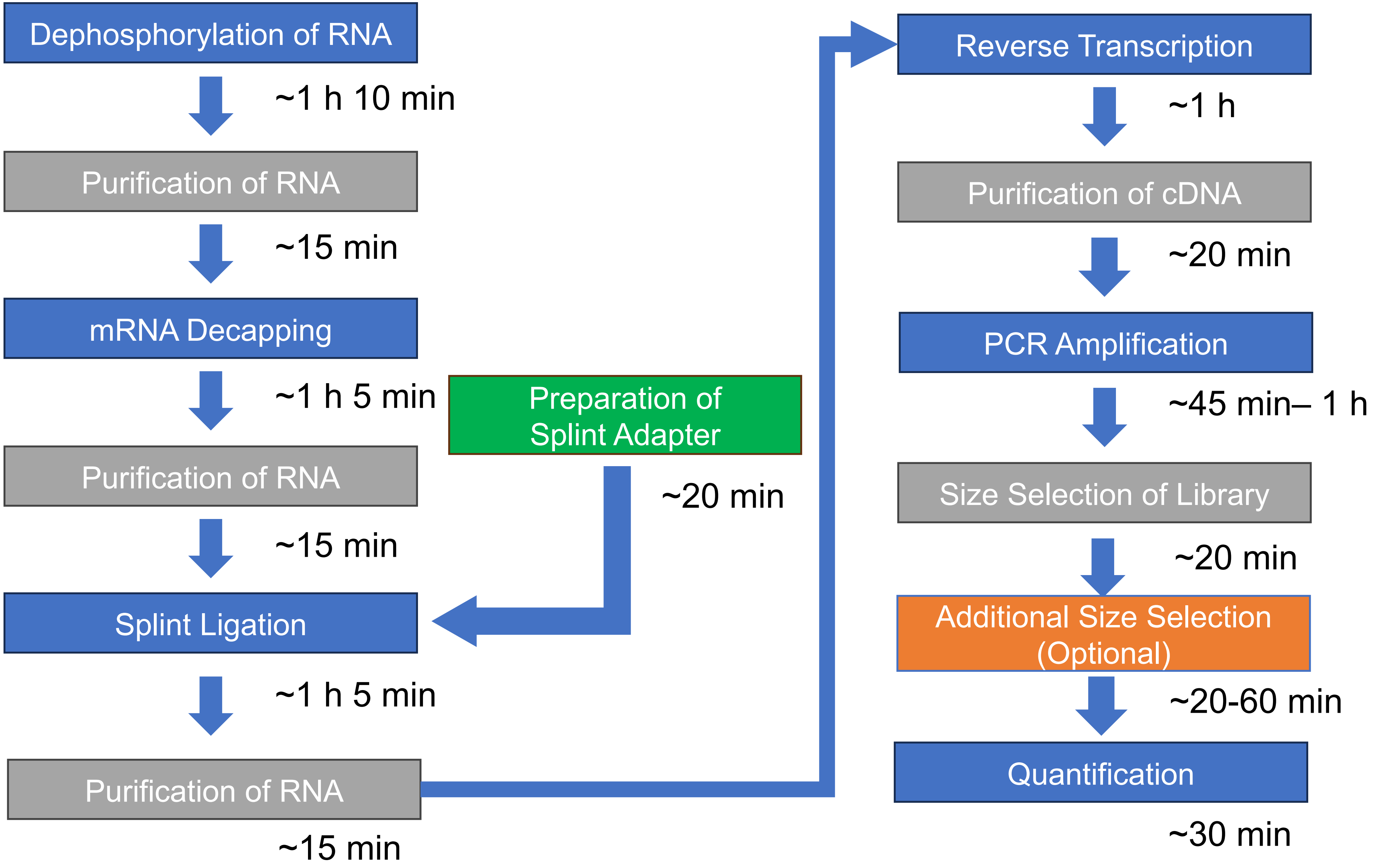

Supplementary Figure 26. Workflow of TSS-seq2 library preparation

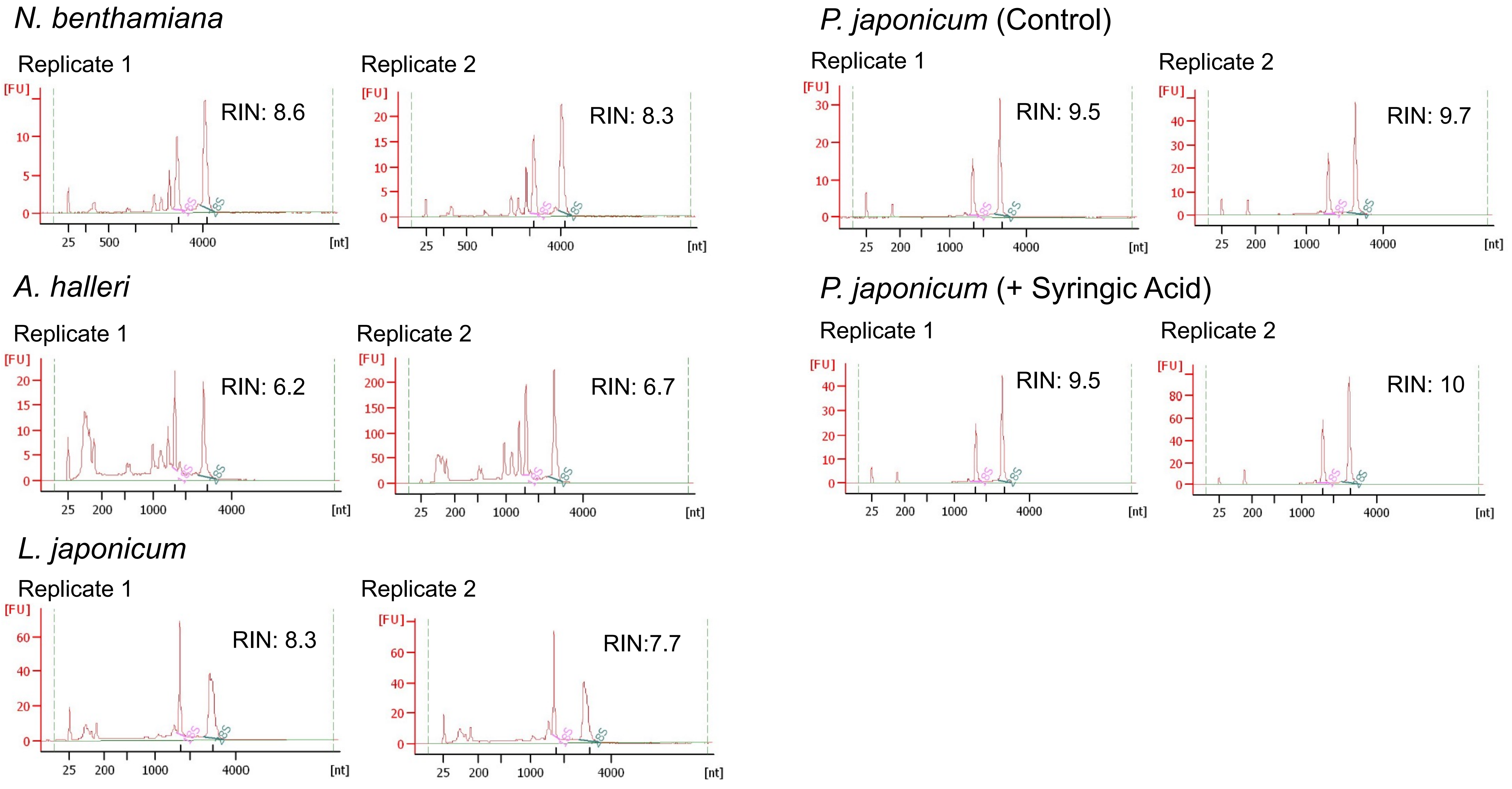

Supplementary Figure 27. Quality of plant total RNA

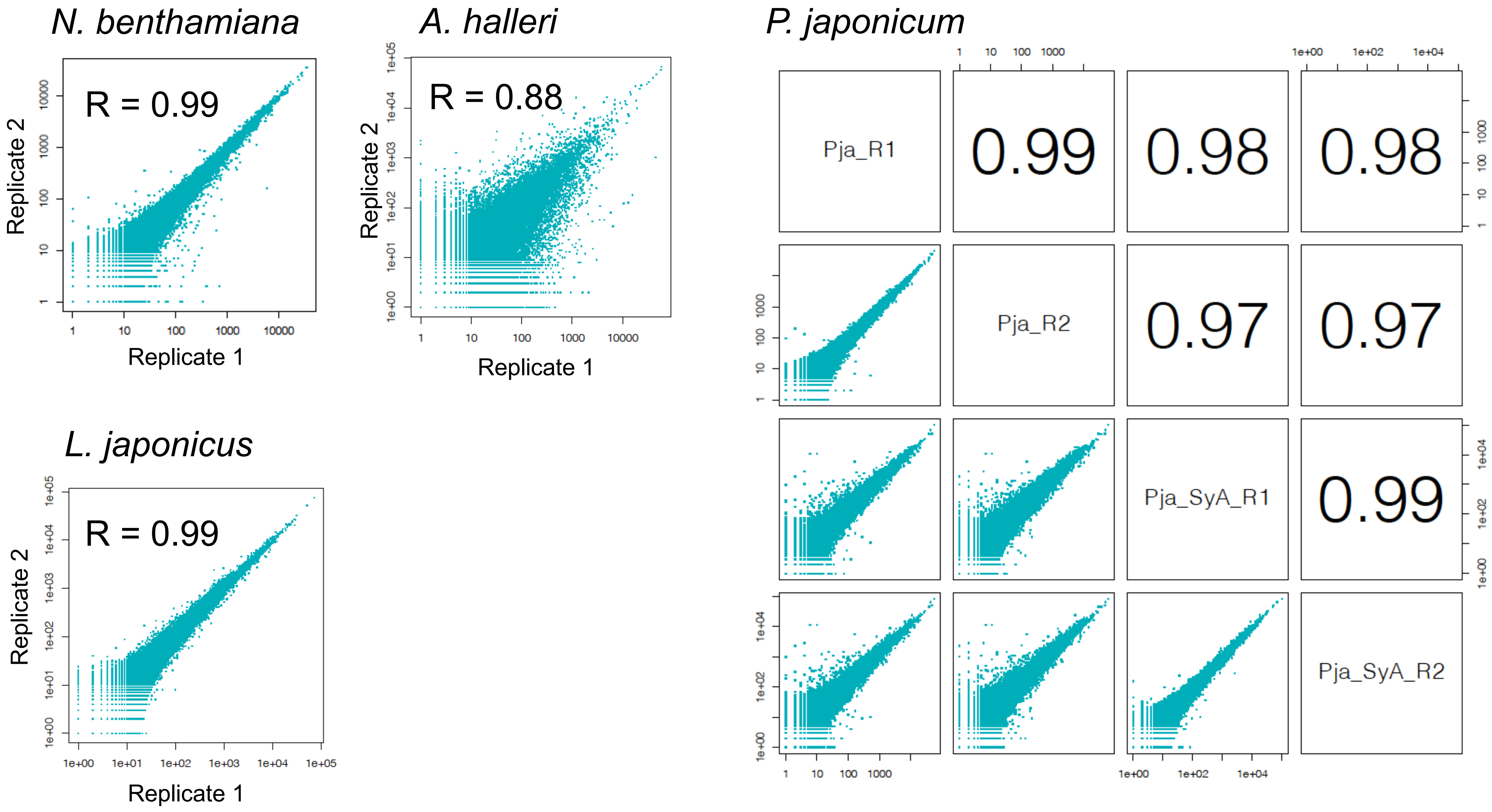

Supplementary Figure 28. Correlation of TSS counts between plant sample replicates

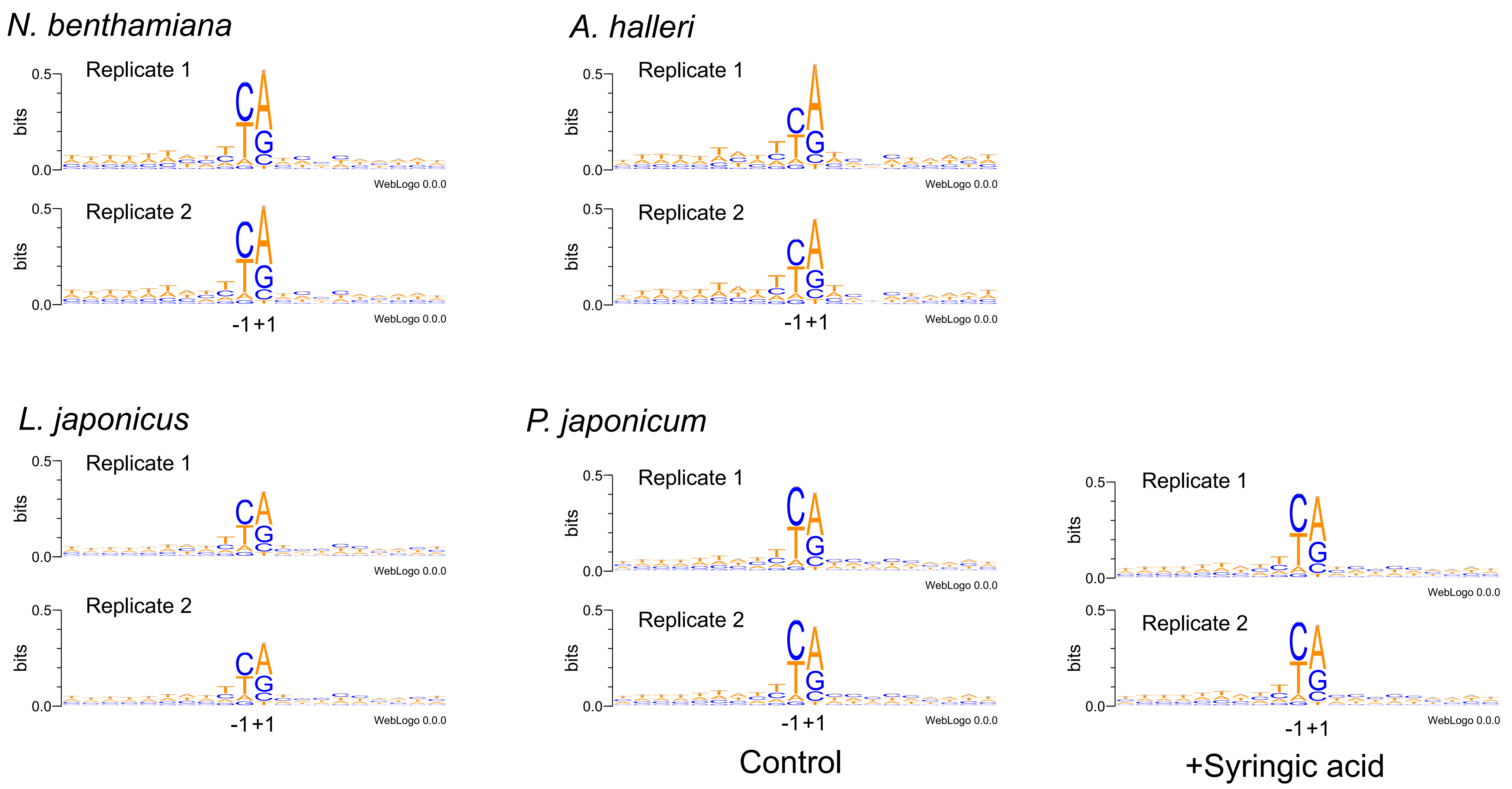

Supplementary Figure 29. TSS consensus sequences for plant samples

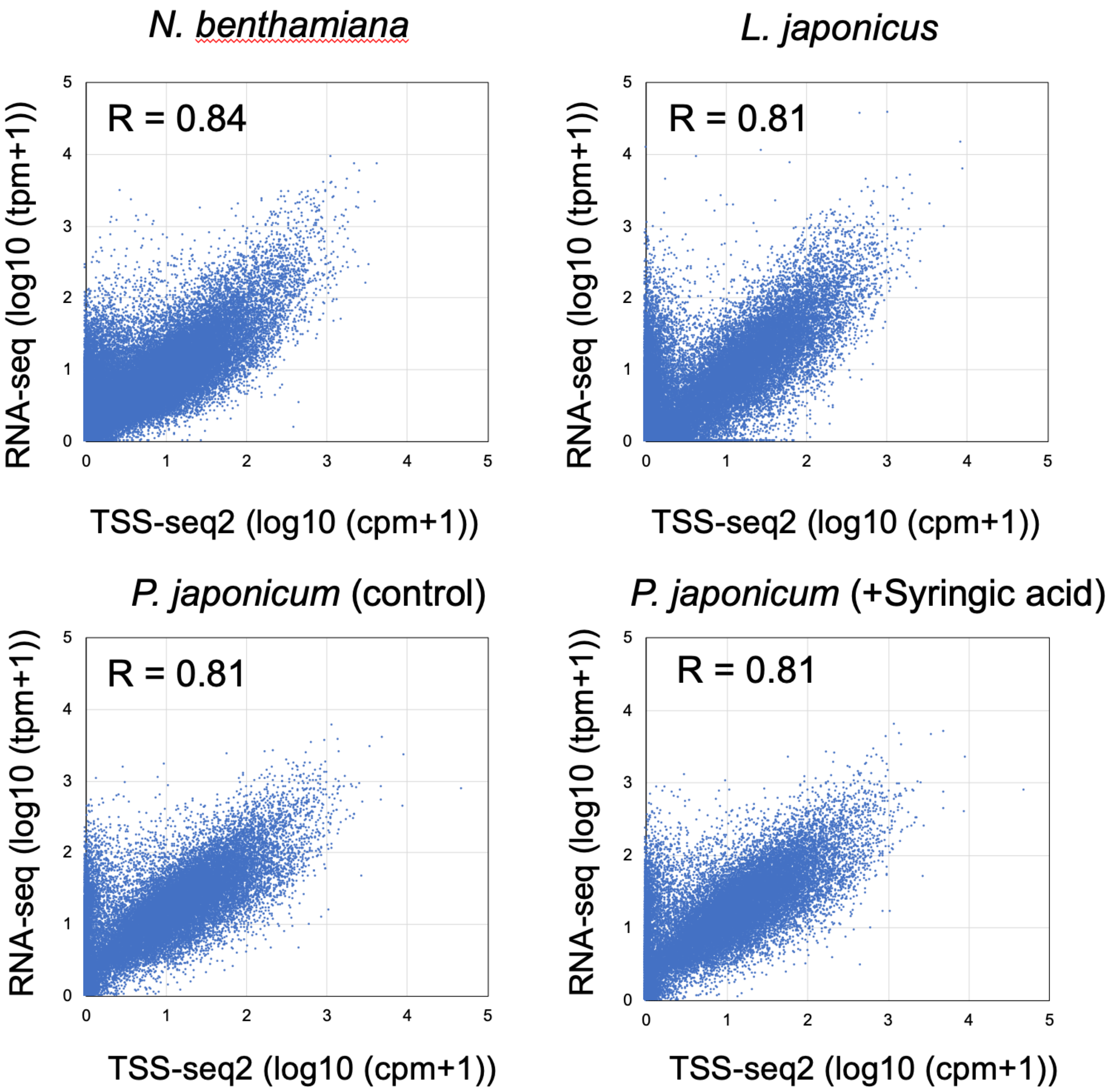

Supplementary Figure 30. Expression estimates for RNA-seq and TSS-seq2 analysis of plant samples

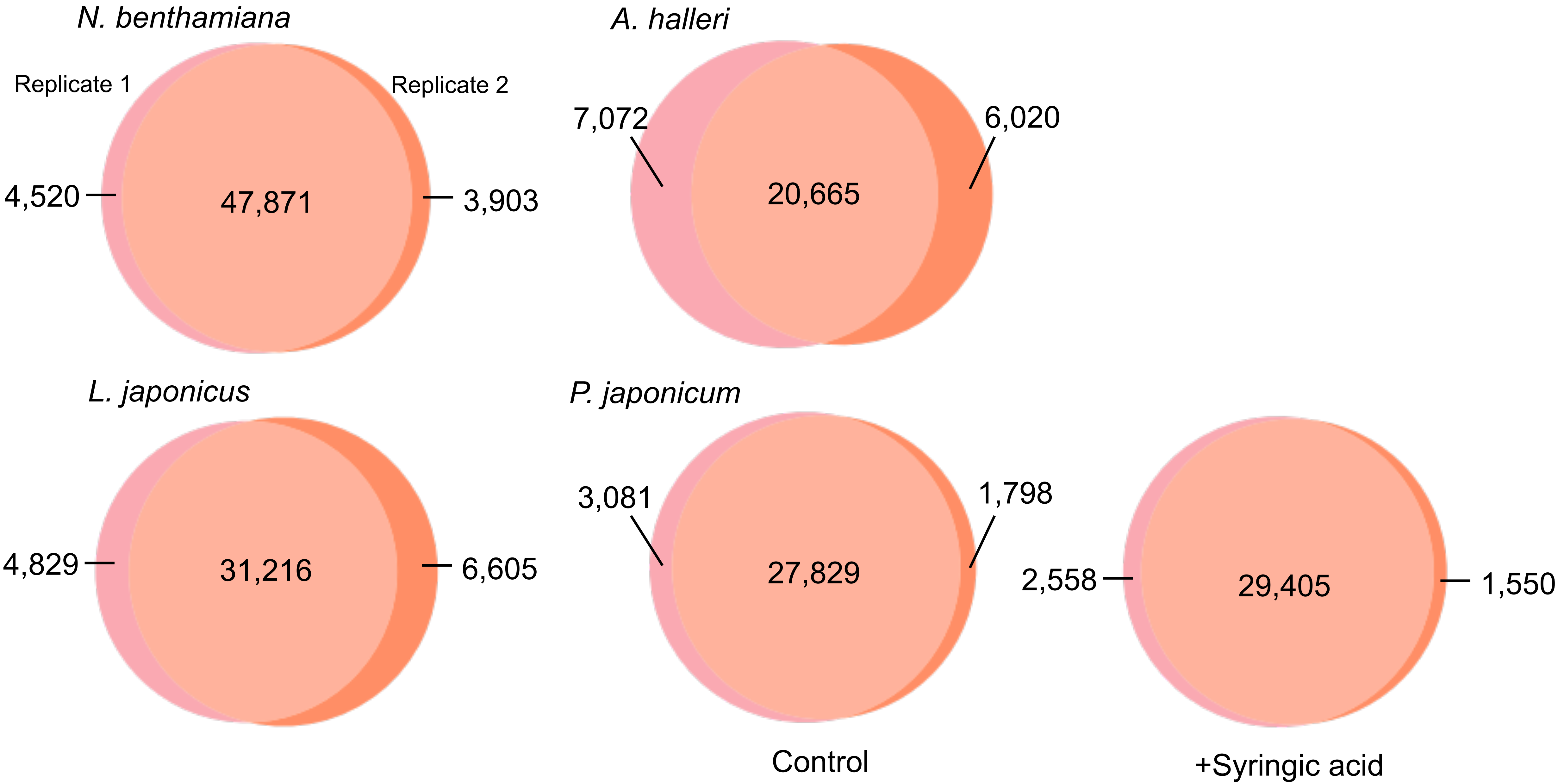

Supplementary Figure 31. Overlapping TSCs between replicates in plants

A

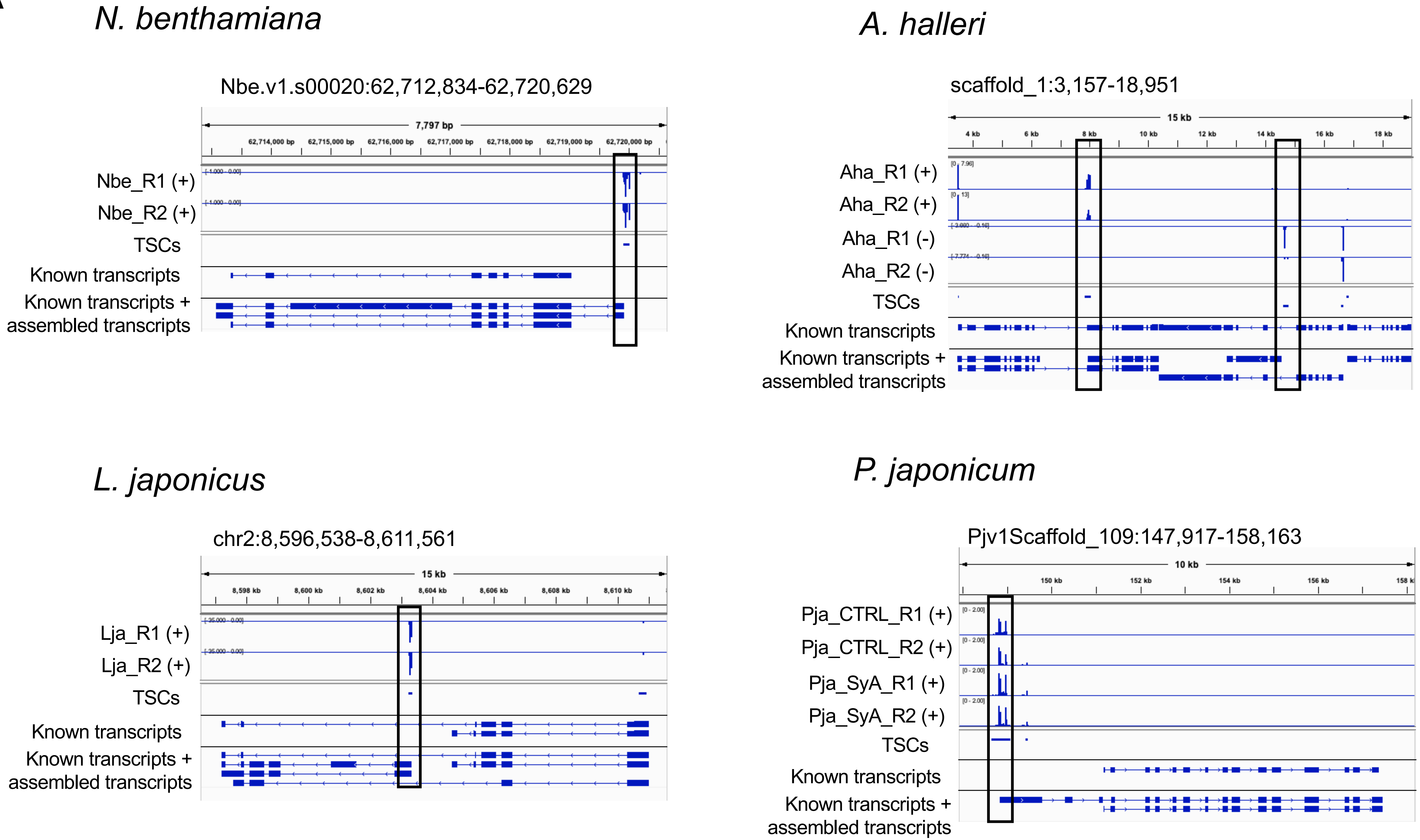

Supplementary Figure 32. Unannotated TSCs detected by TSS-seq2 in plants (1/2)

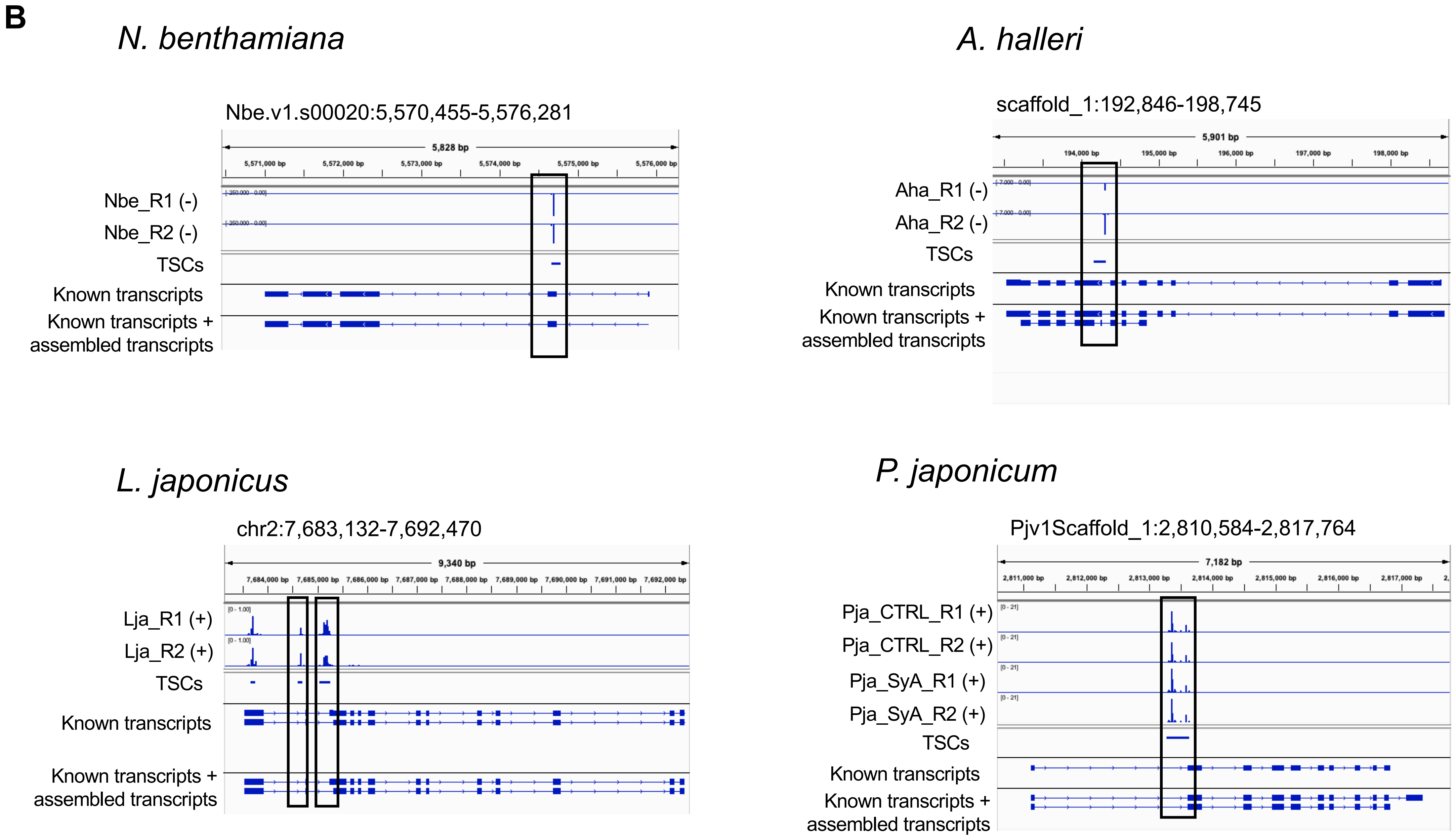

Supplementary Figure 32. Unannotated TSCs detected by TSS-seq2 in plants (2/2)

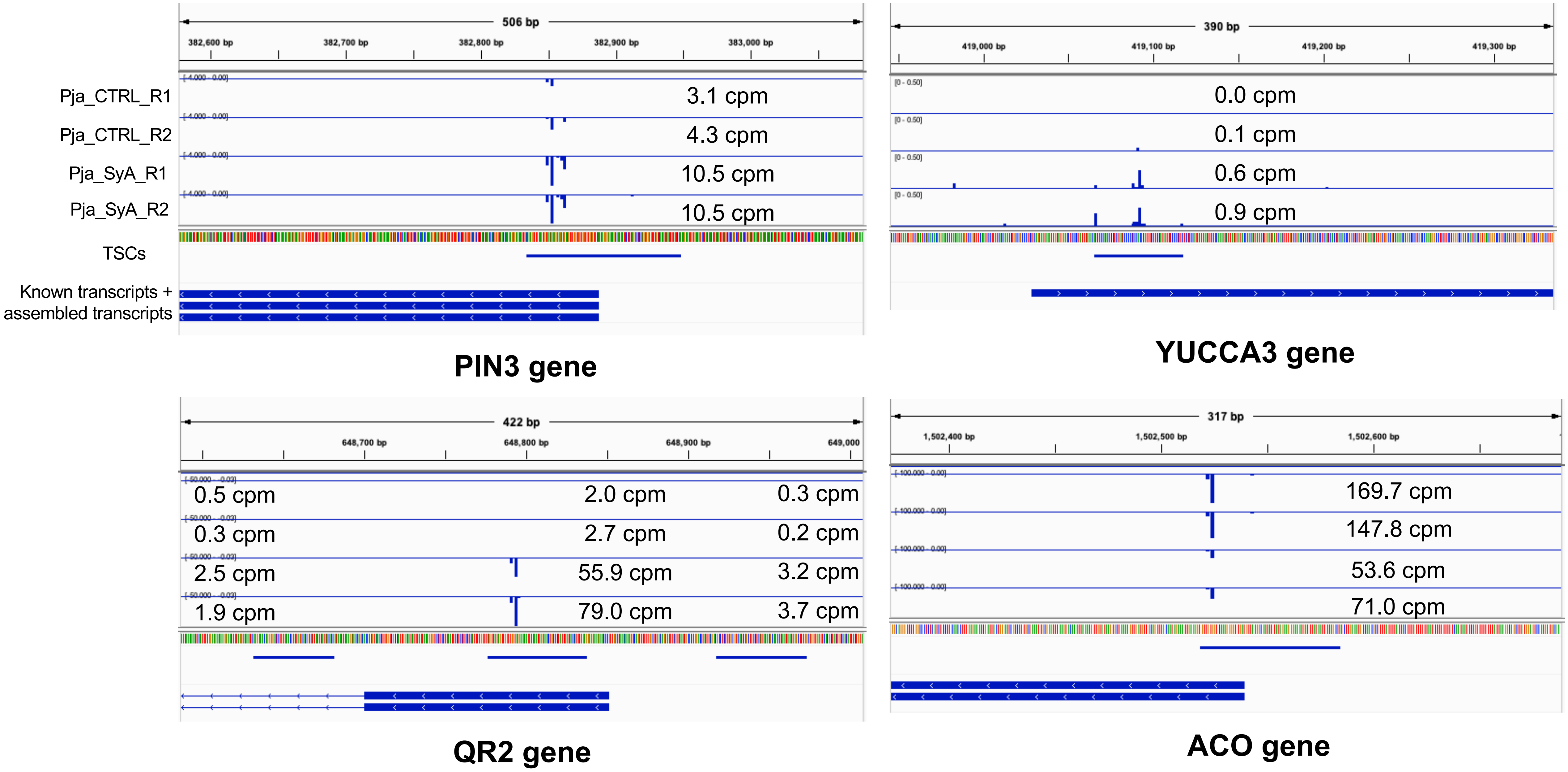

Supplementary Figure 33. TSCs showed expression changes in response to induction of prehaustoria in *P. japonicum*
